# Lifespans of passenger cars in Europe: empirical modelling of fleet turnover dynamics

**DOI:** 10.1186/s12544-020-00464-0

**Published:** 2021-01-25

**Authors:** Maximilian Held, Nicolas Rosat, Gil Georges, Hermann Pengg, Konstantinos Boulouchos

**Affiliations:** 1 ETH Zurich, Institute of Energy Technology, Laboratory for Aerothermochemistry and Combustion Systems, Sonneggstrasse 3, Zurich, 8092 Switzerland; 2 Managing Director Audi e-gas Betreibergesellschaft m.b.H., Audi AG, I/EG-X, Ingolstadt, 85045 Germany

**Keywords:** Passenger car, Vehicle, Lifespan, Lifetime, Survival rate, Scrapping, Export, Used car market, Spillover, Europe

## Abstract

Cars have a high share of global transport-related CO_2_ emissions. To model the market diffusion of new energy carriers and powertrains like electric vehicles, fleet turnover models are commonly used. A decisive influence factor for the substitution dynamics of such transformations is the survival rate of the national car fleet of a country. It represents the likelihood of a car reaching a certain lifespan. Due to a lack of data, current methods to estimate such survival probabilities neglect the imports and exports of used cars. Existing studies are limited to countries with a predominant market of new cars, compared to low numbers of imported and exported used cars. In this study, we resolve this marked simplification and propose a new method to estimate survival probabilities for countries with a high number of imported and exported used cars. Empirical data on the car stock, on inflows of new and used cars, and on outflows of exported and scrapped cars are gathered from 71 national statistics offices. Survival rates of the car fleets of 31 European countries are derived, for which we find a pronounced regional variability. Average lifespans of cars vary from 8.0 to 35.1 years, with a mean of 18.1 years in Western and 28.4 years in Eastern European countries, revealing the high impact of cross-border flows of cars. The study also shows that survival rate estimates can be improved significantly even in the absence of reliable data if a combination of a Weibull and a Gaussian distribution is used. It is likely that the predictive power of existing models (regarding the future environmental impact of car fleets) could be improved significantly if these findings were considered accordingly. The findings of this study can directly be included in fleet turnover and policy assessment models. They also enable the analysis of economic and environmental spillover effects from the imports and exports of used cars between countries.

**Supplementary Information:**

The online version contains supplementary material available at (10.1186/s12544-020-00464-0).

## Introduction

### Motivation

Passenger cars make up the bulk of transport CO_2_ emissions in almost all European countries [1]. Electrification might change this [2–4], but its effectiveness is limited by how quickly new vehicles enter the fleet and displace current combustion engine vehicles [5, 6]. The remaining lifetime of vehicles defines the amount of locked-in emissions from existing infrastructure [7, 8] and determines the market penetration of new technologies [9–12]. Accurate modeling of the substitution dynamics of the car fleet is thus key for climate policies [13].

In literature, cohort models are the instrument of choice [14–17]: they understand the car stock as the set of all cars registered in a country at a given time. New cars join, as people buy them from manufacturers and register them in the country (*new registrations*) or *import used cars* from foreign markets^1^. After a certain number of years in service, the vehicles leave either for foreign markets (*exports of used cars*) or for being dismantled (*scrapped cars*). National used car markets, i.e. the change of ownership within a country, are neglected since they do not alter the composition of the fleet. In such a framework, the substitution dynamics are governed by the number of cars entering the fleet and their cumulative survival probability (CSP), i.e. the likelihood of a car reaching a certain lifespan in a given fleet. The CSP is a property of the fleet and the car owners of its host country, not the vehicles, since cars are typically sold on long before they fail irreparably. Instead, with more fuel-efficient cars being introduced to the market of richer countries, a flow of old cars to poorer countries can be observed. [18] In Europe, used cars are on average sold from West to East, where cars live longer due to different economic circumstances. This results in higher average car ages in Eastern European countries [19].

### Existing literature

Multiple studies highlight the importance of the used car market and suggest further research on this topic [16, 20, 21]. Global trade of “used vehicles has been [...] valued at over USD 17.6 Billion in 2014” [22], and in the EU, the used car market volume is substantially larger than the new car market [18]. Imports and exports of used cars also couple car markets of multiple countries. The German scrappage scheme in 2009 revealed the spillover effects that a national policy can have on other countries. A “strong decrease of exports in 2009 [... reduced] the supply of used car significantly”, but was not compensated by “a shift from used to new car purchases” [18]. National policies can reduce the domestic transport CO_2_ emissions, but also have an effect on the markets and emission balances of other countries (see further discussion of the impact of scrappage schemes [23, 24] in Appendix A of theSupporting Information (SI)).

In multi-country analyses of future fleet compositions it is essential to model country-specific survival rates and cross-border flows of im-/exported cars. The two most prominent models to assess the impact of potential CO_2_ reduction policies for the European transport sector are PRIMES-TREMOVE [25–27] and TRIMODE [28, 29], which are used by the European Commission. However, even PRIMES-TREMOVE does not apply country-specific survival rates, but is “based on data provided for Germany, due to the lack of suitable information for the rest of the countries” [25]. It is also neither considering the import nor export flows of used cars between countries [30]. Particularly the imports of used cars, however, perturbs the CSP of a country. This has been largely neglected in literature so far and named “a future challenge for more comprehensive analysis.” [16]

Existing studies on car survival rates are focused on countries with a traditionally high importance of the domestic car industry and hence a predominant position of the *new* car market against the *used* car (import) market, like the United States [31, 32], Japan [21, 33], China [9, 34, 35], and Germany [36]. There is a trade-off between the depth of analysis and the geographic scope (number of countries covered): detailed studies of survival rates with a high data resolution are usually limited to individual regions (e.g. metropolitan areas [37, 38]) or single countries [9, 17, 21, 31–34]. On the contrary, broader studies covering multiple countries usually have to compromise in their data resolution since the required fleet data are cumbersome to obtain due to a lack of harmonized, comprehensive data sets for multiple countries. There are particularly two multi-country studies of interest: Huo & Wang [39] compare survival rates for 4 countries; Oguchi & Fuse [16] provide survival rates for 17 countries. Oguchi & Fuse [16] find that survival rates are changing substantially over time. However, their data stem from 2008 and there is no follow-up study covering a similarly broad analysis in terms of the number of countries studied.

### Contribution of this study

This study adds a new perspective to current modelling approaches of the substitution dynamics of a car fleet. In particular for highly interlinked European countries, the shortcoming of neglecting im-/exports of used cars poses a limitation to the significance of the results of multi-country analyses. We propose a new method to estimate car survival rates for countries for which the car market is dominated by the imports of used cars. The proposed method allows future studies on car lifespans to include countries with high cross-border flows of used cars and to analyse the effects of cross-border linkages of national car sectors [22].

We provide a comprehensive study on survival rates of the national passenger car fleets of 31 European countries. This work covers the European Union (EU-27, except Bulgaria which is excluded due to a lack of data), the United Kingdom (UK), and the European Free Trade Association (EFTA). The large-scale data collection of this study fills the research desideratum highlighted by Mehlhart et al. [30] to assess the differences in survival rates for European countries. Our results are suited to be directly fed to models like PRIMES-TREMOVE and TRIMODE.

## Materials and methods

**Fig. 1:**
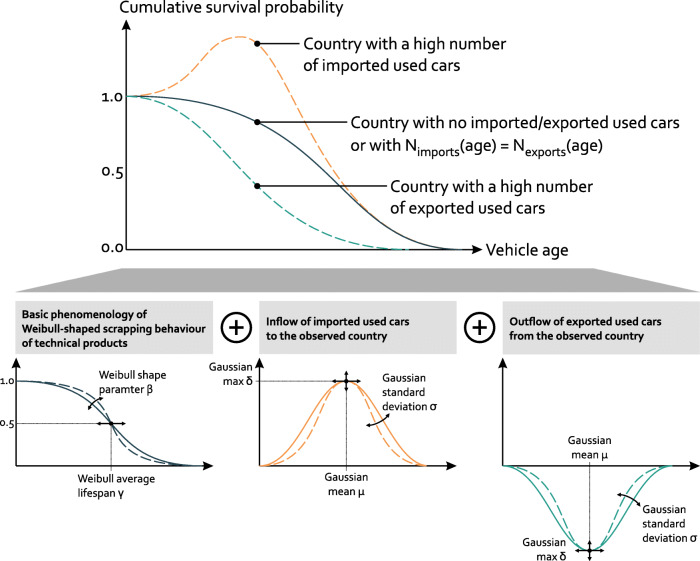
Characteristic cumulative survival probability (CSP) curves. Countries with no imports/exports (dark blue), high imports (orange), and high exports (turquoise) of used cars. The upper panel depicts the raw CSP data resulting from Eq. 1, while the lower panel illustrates how this raw data can be fitted phenomenologically. Fitting parameters *β*,*γ*,*δ*,*μ*, and *σ* refer to the Weibull and Gauss distributions (Eqs. 2 and 3)

New vehicles flow in from the market. Simultaneously, vehicles leave the stock as they are scrapped at a rate dependent on their age. We formalize this process by a Weibull function, which commonly describes aging-related failure modes in reliability engineering. As cars age, maintenance and operation become increasingly more expensive until it becomes more economical to replace or scrap them. When that point is reached depends on the local economic and social context. The CSP is thus not simply a technical property of the cars, but it depends on the host country of the fleet. Some countries have strong export markets: their car owners prefer selling their used cars to foreign markets, long before their “natural” retirement age in the national market. Conversely, some countries have strong import markets: instead of buying brand new cars from local distributors, buyers prefer imported used cars from foreign markets. In Fig. 1, we show the impact of such effects on the CSP of a country. These effects are discussed in the following sections.

### Current standard CSP estimation method

In countries with neither a strong import nor export market, we can directly infer the CSP from the annual stock data, by comparing the number of vehicles of a certain vintage still in the stock to the number of vehicles sold in the corresponding year of first registration [16, 34]:

1 $$ {CSP}_{t}(a) = \frac{N_{t}(a)}{R_{t-a}}   $$

with *N*_*t*_(*a*) as the number of cars of age *a* in the stock at time *t*, and *R*_*t*−*a*_ as the number of new car registrations at time *t*−*a*. In this study, we observe the age distribution of the car stock in *t*=2016.

For countries with a negligible number of imported used cars – and for those where the same number of used cars is imported and exported at identical age distributions – the CSP curve defined by Eq. 1 can be fitted by a Weibull function [16]:

2 $$ \mathcal{W}(\beta,\gamma, a) = exp\left(-\left(\frac{a}{\gamma}\right)^{\beta} \cdot \Gamma\left(1+\frac{1}{\beta}\right)^{\beta} \right)   $$

with *a* as the car age, *β* as the shape parameter, and *γ* as the average lifespan of the Weibull distribution. *Γ* denotes the Gamma distribution.

### New CSP estimation method

If this procedure is applied to countries with a strong import market of used cars, the outcome follows no longer a Weibull distribution: in any given year, the imported used cars add a significant volume of vehicles to the stock that were built before the current year, pushing the CSP beyond 100% (orange curve in upper panel of Fig. 1). While existing studies only consider countries without this effect, we explain it phenomenologically. Imported used cars add a Gaussian on top of the Weibull distribution (orange curve in lower panel of Fig. 1), expressing that there is a preferred age for imported used vehicles^2^:

3 $$ {}\begin{aligned} \mathcal{N}(\delta, \mu,\sigma, a) &= k\cdot\mathcal{N}(\mu,\sigma, a)\\ &= \delta \cdot exp\left(- 0.5 \left(\frac{a-\mu}{\sigma}\right)^{2} \right) \end{aligned}  $$

with $\mathcal {N}(\mu,\sigma, a)$ as the Normal Gaussian distribution with mean, *μ*, standard deviation, *σ*, and car age, *a*. *k* defines a stretch in y-direction which is lumped to the more intuitive maximum of the Gaussian curve, $\delta = \frac {k}{\sqrt {2\pi }\sigma }$.

Analogously, exported used cars add a negative Gaussian (turquoise curve in lower panel of Fig. 1) to the Weibull curve. While the positive Gaussian of imported used cars directly affects the shape of the CSP curve, a negative Gaussian from exported used cars is likely to still result in a Weibull-shaped CSP curve. Without additional data (see next section), we cannot derive the export-related negative Gaussian. Hence, we limit our data-fitting approach to a Weibull distribution or the sum of a Weibull and a import-related Gaussian:

4 $$\begin{array}{*{20}l} {}CSP(a) \longleftarrow \mathcal{W}(\beta,\gamma, a) &\ \text{for countries w./o.}\\[-4pt] &\ \ \text{considerable imports}  \end{array} $$

5 $$\begin{array}{*{20}l} {}CSP(a) \longleftarrow \mathcal{W}(\beta,\gamma, a) & + \mathcal{N}(\delta, \mu,\sigma, a)\\ &{} \text{for countries w./ cons. imports}  \end{array} $$

Since market diffusion models focus on the lifetime of cars in a specific country, where it is irrelevant whether used cars leave the national fleet because they are scrapped or exported, correcting only for imports is sufficient for such countries. Then, the CSP curve reflects a *fleet exit rate*.

Formalizing raw CSP data with a Weibull and a Gaussian distribution has two main advantages. First, it adds a phenomenological description of the imported used cars to the standard Weibull-shaped CSP. Second, it adds predictive power to the CSPs used in market diffusion models: the parametrized curves can be adjusted over time to reflect different market conditions, scrapping patterns, and policies like scrappage schemes.

The CSPs for all 31 countries covered in this study are computed for discrete ages using Eq. 1. Continuous parametrized curves are fitted to this CSP data using Eqs. 4 and 5. To make the fitting procedure computationally more efficient, lower and upper bounds are defined for the fitting parameters (see further information in Appendix F): *γ*∈ [5,40], *β*∈ [2,6], Gaussian stretch $k = \delta \cdot \sqrt {2\pi }\sigma \in $ [2,10], *μ*∈ [5,30], *σ*∈ [5,30]. The Weibull and Gaussian distributions are fitted sequentially, first maximizing the *R*^2^ of the Weibull fit and fixing the optimal Weibull parameters, then maximizing the *R*^2^ of the sum of the Weibull and Gaussian.

### Proof of concept of new CSP estimation method

The phenomenological reflection of im-/exported used cars in the CSP curve of a country in form of a Weibull-Gauss-shaped distribution can be tested empirically using additional data. We use age-resolved historical time series of imports and exports of used cars to correct the CSP definition of Oguchi & Fuse [16] (Eq. 1):

6 $$ CSP(a) = \frac{N_{t}(a)}{R_{t-a}^{new}+{Imp}_{t-a}^{used} - {Exp}_{t-a}^{used}}   $$

where *a* is the car age corresponding to the “manufacturing year” of the car, i.e. the first (initial) registration year (for used imports abroad, otw. domestically). ${Imp}_{t-a}^{used}$ is the number of imported used cars that are in the stock of the observed country at time *t* and that have been registered for the first time (abroad) in the year *t*−*a*. ${Exp}_{t-a}^{used}$ is the number of exported used cars that have been registered in the observed country in the year *t*−*a* but have been exported until year *t*.

In line with our theory outlined in Fig. 1, our hypothesis is that the CSP curve resulting from Eq. 6 follows a Weibull shape again since the number of new registrations per year is corrected by imported and exported cars, which cause Gaussian contributions when computing the CSP via Eq. 1.

For nine countries, we could gather age-resolved imports data of used cars; for two countries we could gather such data for exported used cars. This allows for a proof of concept for the definition of the fleet exit rate (correcting only for imports). In the results section, we will see that our hypothesis is verified when using this additional data. For exports-dominated used car markets we expect that the CSP correction using Gaussian distributions is valid as well. As soon as more data becomes available, future research should verify that. Additionally, the superposition of imports and exports effects (positive and negative Gaussians) should be analysed in the future.

### Used data to estimate CSPs via Eq. 6

National governments track their fleets. Organisations like the United Nations Economic Commission for Europe (UNECE), the European Statistical Office (Eurostat), or the European Automobile Manufacturers’ Association (ACEA) collect such data from multiple countries. For our CSP calculation procedure, their data often does not comprise enough detail – e.g. because they lump together historical data, or only have limited historical time series. Both cases add uncertainty to the shape of the CSP curve. Furthermore, UNECE and Eurostat sometimes lack data harmonization among countries [18], stemming from different data providers and different definitions of “new registrations”, which includes imported used cars for some countries, while it does not for others.

A more accurate CSP estimation requires highly resolved data, and hence, cumbersome data gathering and harmonization for multiple countries from statistical offices [30]. Within this study, data from 71 different national statistics (NatStat) sources were acquired from national statistics offices, ministries of interior or transport, national automotive associations, national automotive companies, and other national data collection institutions. Appendix C-E provide an overview on the data sources, their resolution, and the used pre-processing steps for the following data required to estimate CSPs (sorted from good to very limited availability): (1) historical time series of the total number of new registrations up to 2016, (2) the number of cars in the car stocks in 2016, resolved by car age, (3) historical time series of the imports and exports of used cars up to 2016, resolved by car age. For each country, we chose the data sources with the highest resolution in order to compute the CSP.

### The simplified CSP estimation method

Simplified estimation methods are useful to estimate the CSP of a country even if only limited data are available. The one parameter that is of most interest for market diffusion models is the average lifespan of cars, *γ*. To compute *γ*, Eq. 1 shows the need for both registration and stock data. Oguchi & Fuse [16] present a simplified estimation method with less data requirements for countries without considerable im-/exports. In this study, we validate the simplified estimation method of Oguchi & Fuse [16] and demonstrate how the Weibull and Gaussian curves can be approximated even for countries that feature a high number of im-/exported used cars and a dearth of data required for the more sophisticated approach of Eq. 6.

## Results and discussion

This section is organized as follows: First, the results of our new CSP estimation method are discussed. Second, we provide a proof of concept that imports and exports of used cars can be modelled by the sum of a Weibull and a Gaussian curve. Third, a simplified CSP estimation method is derived from our results, which allows for a first-order approximation of the CSP of countries with limited data availability.

### The new CSP estimation method

**Fig. 2:**
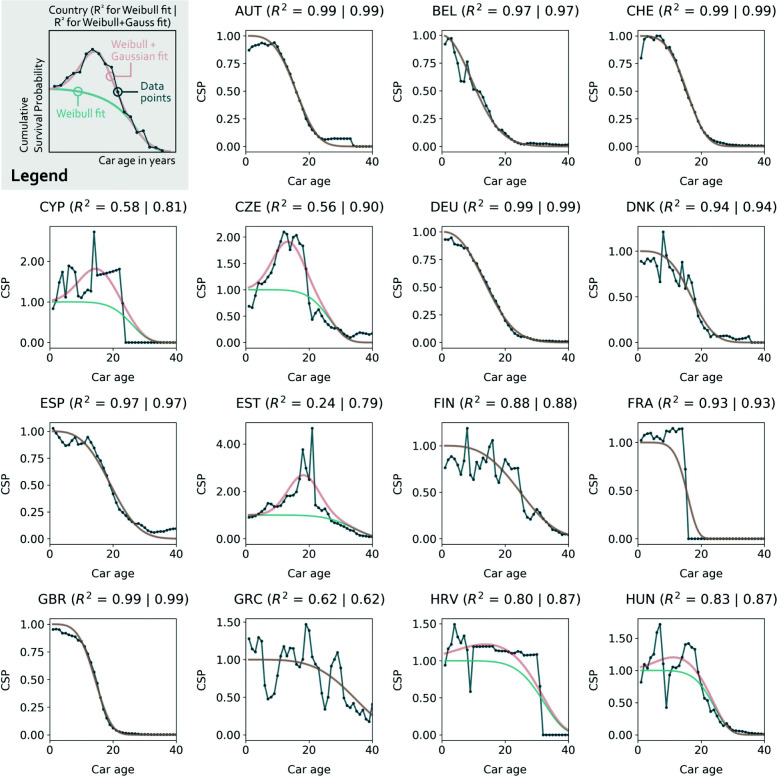
Cumulative survival probability (CSP) data for European countries (1/2). A Weibull curve (turquoise) and the sum of a Weibull and a Gaussian curve (red) are fitted to the data (dark blue)

**Fig. 3:**
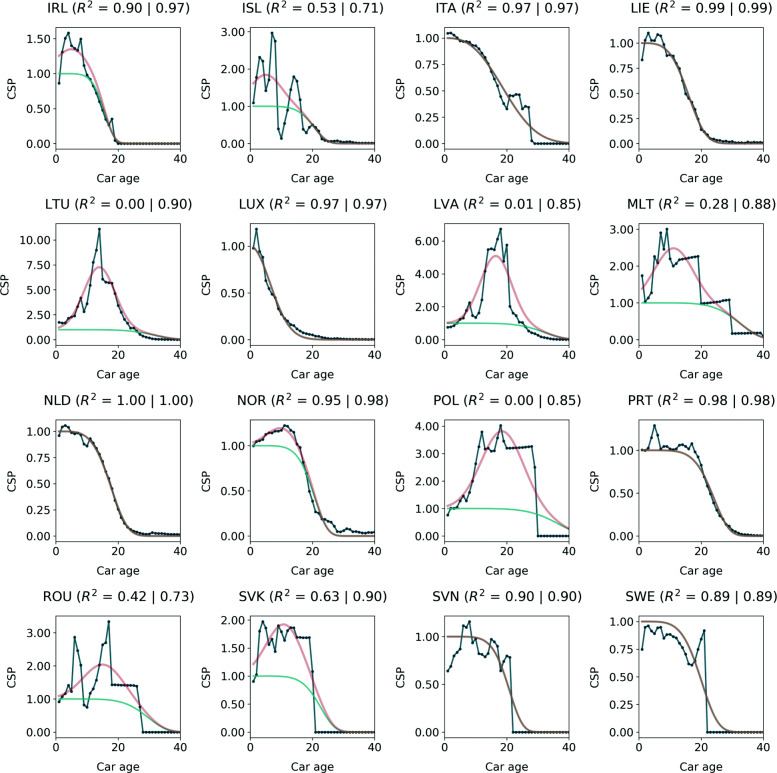
Cumulative survival probability (CSP) data for European countries (2/2). A Weibull curve (turquoise) and the sum of a Weibull and a Gaussian curve (red) are fitted to the data (dark blue)

The raw CSP data are fitted by Weibull and Weibull-Gaussian curves for all 31 European countries, see Figs. 2-3. The corresponding fitting parameters are documented in Table 1. Note that abrupt decreases of the CSP data points to values of zero result from a low resolution of the underlying data, foremost a discontinuous age resolution of the stock (see Appendix C and D). Fitting a Weibull curve to countries with a high number of imported used cars results in poor *R*^2^ values. Adding the Gaussian curve improves the goodness of fit drastically. The mean *R*^2^ value increases from 0.73 (standard deviation of 0.33) to 0.90 (std. dev. of 0.09). While the upper half of the *R*^2^ distribution (>50th percentile) is largely unaffected, the lower half is drastically improved: 5th (25th) *R*^2^ percentiles of 0.00 (0.57) for the sole Weibull fits compare to 0.72 (0.87) for the sum of the Weibull and Gaussian curves.

**Table 1:** Cumulative Survival Probability (CSP) fitting parameters

	Weibull			Import-Gaussian				*R*^2^ values	
Country	*γ*	*β*		*δ*	*μ*	*σ*		$R^{2}_{W}$	$R^{2}_{WG}$
AUT	15.9	3.4		—	—	—		0.99	0.99
BEL	11.7	2.0		—	—	—		0.97	0.97
CHE	15.4	3.6		—	—	—		0.99	0.99
CYP	25.0	6.0		0.8	14.8	5.7		0.58	0.81
CZE	25.5	6.0		0.9	13.4	5.0		0.56	0.90
DEU	14.8	2.4		—	—	—		0.99	0.99
DNK	16.9	3.4		—	—	—		0.94	0.94
ESP	19.4	3.2		—	—	—		0.97	0.97
EST	32.8	6.0		1.7	18.3	5.0		0.24	0.79
FIN	24.9	3.2		—	—	—		0.88	0.88
FRA	15.2	6.0		—	—	—		0.93	0.93
GBR	14.2	4.0		—	—	—		0.99	0.99
GRC	33.9	4.2		—	—	—		0.62	0.62
HRV	30.9	6.0		0.2	14.7	10.6		0.80	0.87
HUN	23.1	6.0		0.2	12.1	6.7		0.83	0.87
IRL	15.0	6.0		0.3	5.0	5.0		0.90	0.97
ISL	19.7	6.0		0.8	5.0	5.0		0.53	0.71
ITA	19.6	2.7		—	—	—		0.97	0.97
LIE	15.6	3.9		—	—	—		0.99	0.99
LTU	30.7	6.0		6.3	14.0	5.0		0.00	0.90
LUX	8.0	2.0		—	—	—		0.97	0.97
LVA	30.9	6.0		4.1	16.5	5.0		0.01	0.85
MLT	31.2	6.0		1.5	11.2	6.2		0.28	0.88
NLD	17.2	4.4		—	—	—		1.00	1.00
NOR	19.8	6.0		0.2	10.2	5.0		0.95	0.98
POL	35.1	6.0		2.8	18.5	6.7		0.00	0.85
PRT	23.1	6.0		—	—	—		0.98	0.98
ROU	28.4	6.0		1.1	15.2	6.4		0.42	0.73
SVK	21.7	6.0		0.9	10.9	5.8		0.63	0.90
SVN	20.0	6.0		—	—	—		0.90	0.90
SWE	19.4	4.9		—	—	—		0.89	0.89

**Legend:** Weibull average lifetime (*γ*), Weibull shape parameter (*β*), Gaussian max (*δ*), Gaussian mean (*μ*), Gaussian standard deviation (*σ*), $R^{2}_{W}$ of Weibull fit (current standard CSP estimation method), $R^{2}_{WG}$ of Weibull-Gaussian fit (new CSP estimation method)

**Fig. 4:**
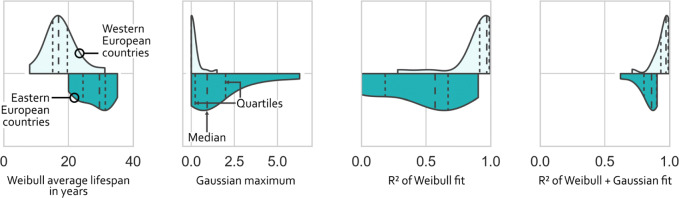
Difference in fitting parameters for Western and Eastern European countries. The group of Western European countries contains AUT, BEL, CHE, DEU, DNK, ESP, FIN, FRA, GBR, IRL, ISL, ITA, LIE, LUX, MLT, NLD, NOR, PRT, and SWE. The group of Eastern European countries contains CYP, CZE, EST, GRC, HRV, HUN, LTU, LVA, POL, ROU, SVK, and SVN

The Weibull average lifespans for the countries that are also covered in Oguchi & Fuse [16] increased in all cases from 2008 (their study) to 2016 (this study), see Appendix F. The increase of 0.2 up to 5.5 years is in line with their finding of increasing lifespans over the last years. The comparison of Luxembourg (LUX) and Lithuania (LTU) shows the diversity of CSPs for European countries. While LUX has the lowest average lifespan of 8.0 years and no Gaussian contribution, LTU comprises of a high average lifespan of 30.7 years and the highest Gaussian contribution of all countries with a Gaussian maximum of 6.3 – compared to the normal Weibull curve which does not exceed values of 1. 14 of the 31 countries have a non-zero Gaussian curve. Those are predominantly Eastern European countries with relatively low *R*^2^ values for the initial Weibull-only fit. Averaged over all countries, the Weibull average lifespan shows a mean of 21.8 years (standard deviation: 7.1 years), with a clear West-East divide that has already been indicated by Vanherle & Vergeer [18] and which is depicted in Fig. 4. A direct comparison of countries’ CSP fits and graphs of the underlying CSP raw data are provided in Appendix F.

Neglecting such a high variability of survival rates in multi-country market diffusion models, like PRIMES-TREMOVE currently does, is a marked simplification of reality. This shortcoming can be overcome using the country-resolved survival rates provided in Table 1.

### Proof of concept of new CSP estimation method

**Fig. 5:**
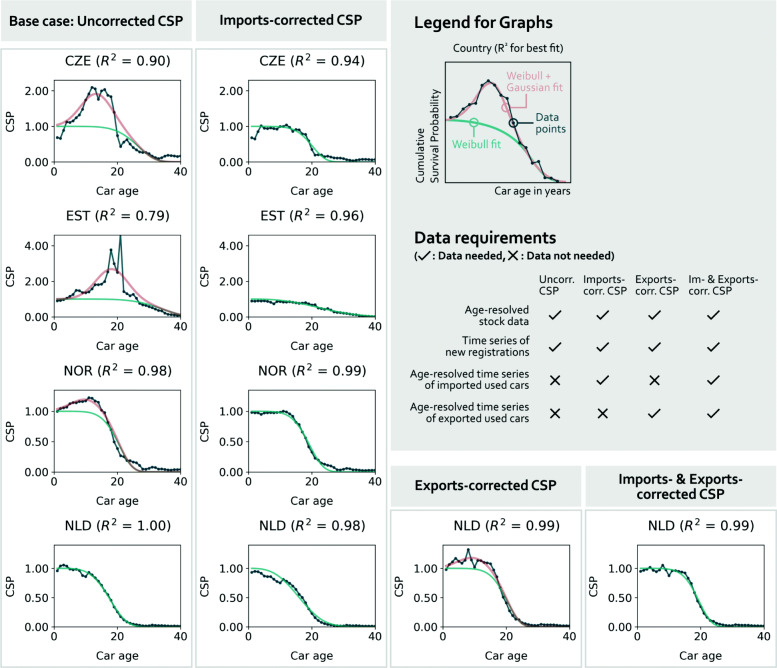
Proof of concept for new CSP estimation method. The uncorrected CSP curve (left column) is corrected by imported and exported cars (two middle columns) and by both (right column), using Eq. 6. Below the legend, the required data to compute the CSP of each column is listed

The vast improvement of the *R*^2^ values of the CSP fits already indicated the capability of our new CSP estimation method to reflect high imports of used cars in the survival rate. This phenomenological description of the survival probability patterns across European countries is further validated in a data-driven proof of concept. We validate whether a correction of the new car market by the import and export market of used cars (Eq. 6) reproduces the results that we obtained by fitting the sum of a Weibull and a Gaussian distribution to the CSP data computed by Eq. 1 (see left column in Fig. 5). We find that both approaches lead to similar results. Out of the nine (two) countries for which age-resolved imports (exports) time series were available, the ones with the highest data availability were chosen for Fig. 5 to illustrate the proof of concept of this new CSP estimation method. All other countries can be found in Appendix G.

The imports correction fully removes the Gaussian contribution of the uncorrected CSP, as suggested by our theory. In the shown cases, the *R*^2^ values stay constant or are even improved. For the Netherlands (NLD), one of the two countries with available exports data, correcting only for exports would add a Gaussian contribution. Correcting for both imports and exports leads to a pure Weibull curve again, as suggested by our theory.

### The simplified CSP estimation method

The simplified CSP estimation method of Oguchi & Fuse [16] shows good results for countries with a predominant position of the new car market, but performs badly for others – see detailed analysis in Appendix H. Our proposed new simplified CSP estimation method is based on correlations from the fitting parameters of the data-fitting approach. *β* and *σ* can be fixed to constant values without a significant loss in accuracy, see Appendix H. Furthermore, we found correlations among fitting parameters and between fitting parameters and GDP per capita [40], the ratio of annual new registrations in a country compared to its stock size, and the cross-border balance, which is defined as the ratio of the net flow of used cars over a country’s border to the total registrations of new and used cars:

7 $$ \text{cross-border balance} = \frac{Imp-Exp}{Imp+Reg}   $$

In our analysis, we use the mean number of new registrations, *Reg*, of imports, *Imp*, and of exports of used cars, *Exp*, over the last ten years to average out outliers. However, using only the most recent data leads to similar results with almost the same goodness of fit.

**Fig. 6:**
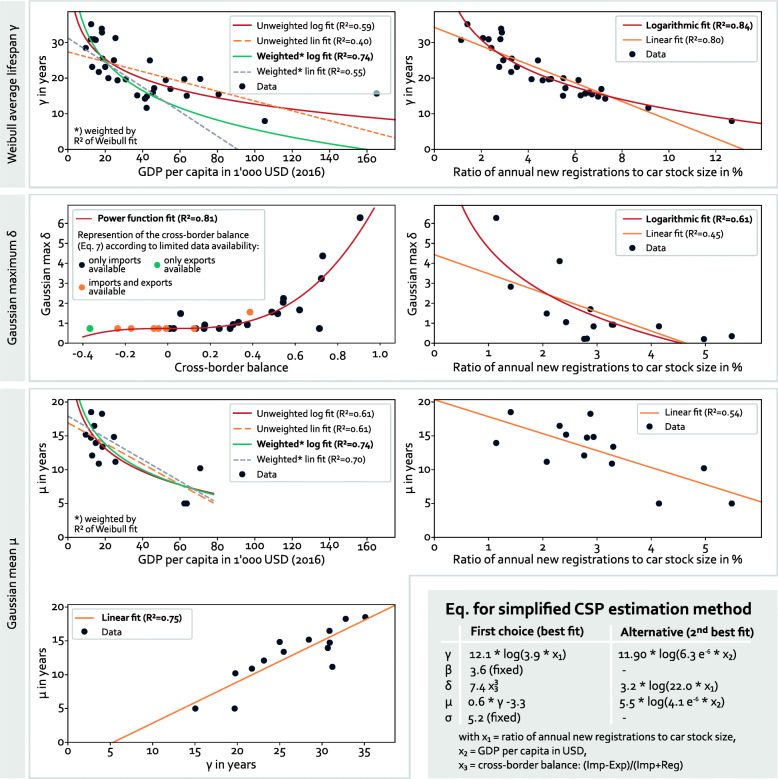
Simplified CSP estimation method. Approximation of fitting parameters by aggregate indicators of a country – equations are given for best fits which are marked in bold in the corresponding plots. Fitting parameters refer to Eqs. 2 and 3

Figure 6 displays the correlations that showed the best fits. Other correlations only showed inferior suitability to predict the CSP curve parameters. For example, we found that neither Weibull nor Gaussian fitting parameters are correlated to countries’ car stock size or their population size. Note that the correlation between the cross-border balance and the maximum of the Gaussian curve, *δ*, in Fig. 6 is based on an incomplete data set. For those countries where only imports data was available (i.e. Exp = 0 in Eq. 7), a shift of the points to lower cross-border balance values would be expected if exports would be considered as well. The opposite holds for the exports-only country (Imp = 0 in Eq. 7, turquoise point).

Figure 6 also provides the set of equations that we suggest to use in order to derive first-order approximations of CSPs in countries with a dearth of data, which impedes the determination of CSPs using our new Weibull-Gauss-based estimation method. The simplified CSP estimation method requires only knowledge about aggregate data. Without the need for any age-resolved data sets, the equations in Fig. 6 may serve as the basis for a first-order approximation of CSPs in countries with a high share of imported used cars.

## Conclusions

This paper resolves two marked simplifications of the substitution dynamics of existing car fleet turnover models. They concern the survival probability of cars in national fleets.

First, accurate information about the survival rates of cars is only known for few countries due to a lack of data. There is no comprehensive analysis for all European countries. Therefore, multi-country fleet turnover models like PRIMES-TREMOVE usually implement a generic survival rate that is assumed to be identical for all countries modelled. In reality, however, there are large differences in survival rates between European countries. This study provides the first comprehensive analysis of survival rates for all European countries (incl. the EU-27, the UK, and EFTA, except of Bulgaria which is excluded due to a lack of data). They are based on a large-scale data collection of stock, new registration, and import/export data from 71 national statistics offices. Our resulting cumulative survival probabilities (CSPs) show that the average lifespans of national car fleets in European countries vary strongly from 8.0 up to 35.1 years.

Second, existing studies on survival rates of cars are focusing on countries with a predominant new car market and excluding those with a high share of imported used cars. The imports and exports of used cars, however, perturb the standard Weibull-shaped survival probability distribution. This is not reflected in current multi-country fleet turnover models since a methodology to describe survival rates for countries with a considerable share of imported/exported used cars is currently missing due to a lack of data. The large-scale data collection of our study reveals that the CSP curves of the car fleets of European countries show wide variations. While survival rates of countries with a negligible number of imported/exported used cars are Weibull-shaped, we show that imported (exported) used cars perturb the Weibull shape, but can be modelled as positive (negative) Gaussian curves on top of the Weibull distribution. This means that the import/export contribution can be approximated using this approach even if data are lacking. Furthermore, we find a strong West-East divide with a flow of used cars from Western to Eastern European countries, resulting in older cars in Eastern European countries, where the average lifespan is approximately 10 years higher than in Western European countries.

The empirical findings of this study are likely to have severe impacts on the results of market diffusion models like PRIMES-TREMOVE. The consideration of the presented country-specific CSP curves in models like PRIMES-TREMOVE would be a value added for an effective design of climate policies in Europe.

For further studies, we identify four research desiderata.

First, the survival rate of the car fleet of a country changes over time [15, 16] – see our exemplary study on CSP curves for 20 consecutive years for Latvia in Appendix I. CSP fitting parameters thus have to be updated regularly, or a function describing the changing fitting parameters over time dependent on aggregate indicators like GDP, motorisation rate, etc. needs to be explored. Such a parametrization could leverage the full predictive power that is inherent to the phenomenological description of a CSP by a Weibull and a Gaussian curve. Short-time market shocks of only few months or years, like the COVID-19 pandemic, are not likely to affect the shape of CSP curves in the long run. This is due to the fact that sudden drops in new registrations are usually followed by subsequent rebounds. Fitting a Weibull (and Gaussian) distribution to the data will smoothen out both effects. However, if such market shocks deteriorated the economic situation of customers persistently, a trend to higher average lifespans could be observed in the subsequent years.

Second, technological disruptions might change the shape of cars’ survival rates in the future. Electric vehicles might be scrapped later due to reduced operational costs and longer component lifetimes (resulting from less extreme operation temperatures, less moving parts, and in general less components compared to an internal combustion engine vehicle). Limited battery lifetimes might be a counteracting effect. Mobility as a service concepts, i.e. cars being shared among many customers, might reduce the average car lifetime since its operational hours per day could increase substantially.

Third, the lack of comprehensive data sets required to compute CSPs has been pointed out in multiple studies. Regarding the need for high-resolution and harmonized statistics, we refer to the recommendations of two reports prepared for the European Commission [30, 41]. In Appendix J, we highlight which data sources show the largest impact on the accuracy of the CSP estimation.

Fourth, the economies of European countries and in particular their automotive industries are highly interlinked. Cross-border flows of used cars induce economic spillovers from exporting to importing countries and alter national fleet compositions and their environmental performance. However, existing multi-country market diffusion and policy assessment models do not take into account cross-border flows of used cars due to a dearth of data. Including imports and exports of used cars in the substitution dynamics of fleet turnover models, this study provides a good foundation to explore such spillover effects. Subsidies for electric vehicles in one country might lead to an increased flow of older cars into importing countries. This, in turn, could result in an undesired increase in CO_2_ emissions in the car sector of the respective country and thwart its national CO_2_ reduction plans. It remains to be explored whether this would entail only a local shift of emissions or whether it would still reduce overall emissions within Europe. Future studies should provide scientific evidence for “the implications of used vehicle flows both for local pollutants and health and global emissions that contribute to atmospheric warming” [22].

## Supplementary Information

**Additional file 1** Supporting PDF. 52 pages comprising a detailed description of the methodology used, the assumptions made, the data used, and additional analyses performed.

**Additional file 2** Supporting Excel sheets. Input data (stock, new registrations, imports and exports of used cars) and resulting survival rates for 31 European countries.

## Data Availability

All data generated or analysed during this study are included in this published article and its supplementary information files.

## Abbreviations

ACEA: European Automobile Manufacturers’Association
CSP: Cumulative Survival Probability
EFTA: European Free Trade Association
EU: European Union
Eurostat: European Statistical Office
NatStat: National Statistics
UK: United Kingdom
UNECE: United Nations Economic Commission for Europe

## Footnotes

1 Note that we refer to “new registrations” as the new registrations of *new* cars, while we refer to “imports of used cars” as the new registrations of *used* cars in the respective country.

2 See discussion in Appendix B why a Gaussian distribution is suited to model the impact of car imports and exports on the CSP curve.

## References

[1] European Environment Agency (2018). National emissions reported to the UNFCCC and to the EU Greenhouse Gas Monitoring Mechanism. https://www.eea.europa.eu/data-and-maps/data/national-emissions-reported-to-the-unfccc-and-to-the-eu-greenhouse-gas-monitoring-mechanism-14#tab-additional-information.

[2] Sims, R., Schaeffer, R., Creutzig, F., Cruz-Núñez, X., D’Agosto, M., Dimitriu, D., Meza, M.J.F., Fulton, L., Kobayashi, S., Lah, O., McKinnon, A., Newman, P., Ouyang, M., Schauer, J.J., Sperling, D., Tiwari, G. (2014). Transport. In: Edenhofer, O., Pichs-Madruga, R., Sokona, Y., Farahani, E., Kadner, S., Seyboth, K., Adler, A., Baum, I., Brunner, S., Eickemeier, P., Kriemann, B., Savolainen, J., Schlömer, S., von Stechow, C., Zwickel, T., Minx, J.C. (Eds.) In *Climate Change 2014: Mitigation of Climate Change. Contribution of Working Group III to the Fifth Assessment Report of the Intergovernmental Panel on Climate Change*. Cambridge University Press, Cambridge, United Kingdom and New York, NY, USA, (p. 614 ff).

[3] Leurent, F., & Windisch, E. (2011). Triggering the development of electric mobility: a review of public policies. *European Transport Research Review*, *3*(4), 221–235. 10.1007/s12544-011-0064-3.

[4] IPCC (2018). In *Summary for Policymakers. In: Global Warming of 1.5*^∘^*C. An IPCC Special Report on the impacts of global warming of 1.5*^∘^*C above pre-industrial levels and related global greenhouse gas emission pathways, in the context of strengthening the global response to the threat of climate change, sustainable development, and efforts to eradicate poverty [Masson-Delmotte, V., P. Zhai, H.-O. Pörtner, D. Roberts, J. Skea, P.R. Shukla, A. Pirani, W. Moufouma-Okia, C. Péan, R. Pidcock, S. Connors, J.B.R. Matthews, Y. Chen, X. Zhou, M.I. Gomis, E. Lonnoy, T. Maycock, M. Tignor, and T. Waterfield (eds.)]*

[5] Figenbaum, E., Fearnley, N., Pfaffenbichler, P., Hjorthol, R., Kolbenstvedt, M., Jellinek, R., Emmerling, B., Bonnema, G.M., Ramjerdi, F., Vågane, L., Iversen, L.M. (2015). Increasing the competitiveness of e-vehicles in Europe. *European Transport Research Review*, *7*(3), 28. 10.1007/s12544-015-0177-1.

[6] Chatzikomis, C.I., Spentzas, K.N., Mamalis, A.G. (2014). Environmental and economic effects of widespread introduction of electric vehicles in Greece. *European Transport Research Review*, *6*(4), 365–376. 10.1007/s12544-014-0137-1.

[7] Tong, D., Zhang, Q., Zheng, Y., Caldeira, K., Shearer, C., Hong, C., Qin, Y., Davis, S.J. (2019). Committed emissions from existing energy infrastructure jeopardize 1.5C climate target. *Nature*, *572*(7769), 373–377. 10.1038/s41586-019-1364-3. 31261374 10.1038/s41586-019-1364-3 PMC6697221

[8] Bullock, S., Mason, J., Broderick, J., Larkin, A. (2020). Shipping and the Paris climate agreement: a focus on committed emissions. *BMC Energy*, *2*(1), 5. 10.1186/s42500-020-00015-2.

[9] He, R., Zhao, Z., Liu, P., Li, Z. (2018). Transport Fuel Supply and Demand of the Passenger Car Sector in China up to 2030: A Modeling Approach. *ACS Sustainable Chemistry & Engineering*, *6*(4), 4633–4647. 10.1021/acssuschemeng.7b03649.

[10] Siskos, P., Capros, P., De Vita, A. (2015). CO2 and energy efficiency car standards in the EU in the context of a decarbonisation strategy: A model-based policy assessment. *Energy Policy*, *84*, 22–34. 10.1016/j.enpol.2015.04.024.

[11] Murakami, S., Oguchi, M., Tasaki, T., Daigo, I., Hashimoto, S. (2010). Lifespan of commodities, part I: The creation of a database and its review. *Journal of Industrial Ecology*, *14*(4), 598–612. https://onlinelibrary.wiley.com/doi/full/10.1111/j.1530-9290.2010.00250.x.

[12] Oguchi, M., Murakami, S., Tasaki, T., Daigo, I., Hashimoto, S. (2010). Lifespan of commodities, part II: Methodologies for estimating lifespan distribution of commodities. *Journal of Industrial Ecology*, *14*(4), 613–626. https://onlinelibrary.wiley.com/doi/full/10.1111/j.1530-9290.2010.00251.x.

[13] Pauliuk, S., Dhaniati, N.M.A., Müller, D.B. (2012). Reconciling sectoral abatement strategies with global climate targets: The case of the Chinese passenger vehicle fleet. *Environmental Science & Technology*, *46*(1), 140–147. 10.1021/es201799k. 22074174 10.1021/es201799k

[14] Watari, T., Nansai, K., Nakajima, K., McLellan, B.C., Dominish, E., Giurco, D. (2019). Integrating Circular Economy Strategies with Low-Carbon Scenarios: Lithium Use in Electric Vehicles. *Environmental Science & Technology*, *53*(20), 11657–11665. 10.1021/acs.est.9b02872. 31577427 10.1021/acs.est.9b02872

[15] Cabrera Serrenho, A., & Allwood, J.M. (2016). Material stock demographics: cars in Great Britain. *Environmental Science & Technology*, *50*(6), 3002–3009. 10.1021/acs.est.5b05012. 26871002 10.1021/acs.est.5b05012

[16] Oguchi, M., & Fuse, M. (2015). Regional and longitudinal estimation of product lifespan distribution: a case study for automobiles and a simplified estimation method. *Environmental Science & Technology*, *49*(3), 1738–1743. 10.1021/es505245q. 25549538 10.1021/es505245q

[17] Fridstrøm, L., Østli, V., Johansen, K.W. (2016). A stock-flow cohort model of the national car fleet. *European Transport Research Review*, *8*(3), 22. 10.1007/s12544-016-0210-z.

[18] Vanherle, K., & Vergeer, R. (2016). Data gathering and analysis to improve the understanding of 2nd hand car and LDV markets and implications for the cost effectiveness and social equity of LDV CO2 regulations - Final Report for: DG Climate Action, Brussels. Technical report. http://www.tmleuven.be.

[19] ACEA (European Automobile Manufacturers Association) (2018). ACEA Report: Vehicles in use: Europe 2018. Technical report, Brussels. https://www.acea.be/statistics/article/report-vehicles-in-use-europe-2018.

[20] Fuse, M., Nakajima, K., Yagita, H. (2009). Global flow of metal resources in the used automobile trade. *Materials Transactions*, *50*(4), 703–710. 10.2320/matertrans.MBW200818.

[21] Nakamoto, Y. (2017). CO2 reduction potentials through the market expansion and lifetime extension of used cars. *Journal of Economic Structures*, *6*(1), 17. 10.1186/s40008-017-0080-0.

[22] UNECE (United Nations Economic Commission for Europe) (2017). Used Vehicles: A Global Overview. Technical report, UNECE2017. https://www.unece.org/fileadmin/DAM/trans/doc/2017/itc/UNEP-ITC_Background_Paper-Used_Vehicle_Global_Overview.pdf.

[23] Van Wee, B., De Jong, G., Nijland, H. (2011). Accelerating car scrappage: A review of research into the environmental impacts. *Transport Reviews*, *31*(5), 549–569. 10.1080/01441647.2011.564331.

[24] Luoma, J., & Sivak, M. (2012). Interactions of environmental and safety measures for sustainable road transportation. *European Transport Research Review*, *4*(4), 189–199. 10.1007/s12544-012-0078-5.

[25] Ntziachristos, L., Mellios, G., Kouridis, C., Papageorgiou, T., Theodosopoulou, M., Samaras, Z., Zierock, K.-H., Kouvaritakis, N., Panos, E., Karkatsoulis, P., Schilling, S., Merétei, T., Aladár Bodor, P., Damjanovic, S., Alain, P. (2008). European Database of Vehicle Stock for the Calculation and Forecast of Pollutant and Greenhouse Gases Emissions with TREMOVE and COPERT - Final report. Technical report, LAT/AUTh, Thessaloniki. http://lat.eng.auth.gr/copert.

[26] E, 3Mlab (2014). PRIMES-TREMOVE Transport Model 2013-2014: Detailed model description - E3MLab/ICCS at National Technical University of Athens. Technical report. http://www.e3mlab.eu.

[27] Gómez Vilchez, J.J., Julea, A., Peduzzi, E., Pisoni, E., Krause, J., Siskos, P., Thiel, C. (2019). Modelling the impacts of EU countries’ electric car deployment plans on atmospheric emissions and concentrations. *European Transport Research Review*, *11*(1), 40. 10.1186/s12544-019-0377-1.

[28] Siskos, P., Capros, P., Zazias, G., Fiorello, D., Noekel, K. (2019). Energy and fleet modelling within the TRIMODE integrated transport model framework for Europe. *Transportation Research Procedia*, *37*, 369–376. 10.1016/j.trpro.2018.12.205.

[29] Martino, A., Williams, I., Fiorello, D., Noekel, K., Capros, P., Siskos, P., Zazias, G., Charalampidis, I., Panagiotis, K., Schade, W. (2018). TRIMODE: integrated transport model for Europe. In *Proceedings of 7th Transport Research Arena TRA 2018, April 16-19, 2018, Vienna, Austria*. http://www.trt.it/wp/wp-content/uploads/2018/05/5-TRA2018_TRIMODE.pdf, Vienna.

[30] Mehlhart, G., Merz, C., Akkermans, L., Jordal-Jorgensen, J. (2011). European second-hand car market analysis: Final Report for DG Climate Action at the European Commission (Contract No. 07.0307/2009/549021/SER/C5, Öko-Institut e.V., Transport & Mobility Leuven, COWI). Technical report. https://www.oeko.de/oekodoc/1114/2011-005-en.pdf.

[31] Greenspan, A., & Cohen, D. (1999). Motor vehicle stocks, scrappage, and sales. *The Review of Economics and Statistics*, *81*(3), 369–383.

[32] Bento, A., Roth, K., Zuo, Y. (2018). Vehicle lifetime and scrappage behavior: Trends in the U.S. used car market. *Energy Journal*, *39*(1), 159–183. 10.5547/01956574.39.1.aben.

[33] Kagawa, S., Nansai, K., Kondo, Y., Hubacek, K., Suh, S., Minx, J., Kudoh, Y., Tasaki, T., Nakamura, S. (2011). Role of motor vehicle lifetime extension in climate change policy. *Environmental Science & Technology*, *45*(4), 1184–1191. 10.1021/es1034552. 21265568 10.1021/es1034552

[34] Zheng, J., Zhou, Y., Yu, R., Zhao, D., Lu, Z., Zhang, P. (2019). Survival rate of China passenger vehicles: A data-driven approach. *Energy Policy*, *129*, 587–597. 10.1016/j.enpol.2019.02.037.

[35] Han, H., HeWu, W., MingGao, O., Fei, C. (2011). Vehicle survival patterns in China. *Science China Technological Sciences*, *54*(3), 625–629. 10.1007/s11431-010-4256-1.

[36] Zachariadis, T., Samaras, Z., Zierock, K.H. (1995). Dynamic modeling of vehicle populations: An engineering approach for emissions calculations. *Technological Forecasting and Social Change*, *50*(2), 135–149. 10.1016/0040-1625(95)00057-H.

[37] Rith, M., Soliman, J., Fillone, A., Biona, J.B.M., Lopez, N.S. (2018). Analysis of Vehicle Survival Rates for Metro-Manila. In *2018 IEEE 10th International Conference on Humanoid, Nanotechnology, Information Technology, Communication and Control, Environment and Management (HNICEM)*. 10.1109/HNICEM.2018.8666408. IEEE, (pp. 1–4).

[38] Goel, R., Guttikunda, S.K., Mohan, D., Tiwari, G. (2015). Benchmarking vehicle and passenger travel characteristics in Delhi for on-road emissions analysis. *Travel Behaviour and Society*, *2*(2), 88–101. 10.1016/j.tbs.2014.10.001.

[39] Huo, H., & Wang, M. (2012). Modeling future vehicle sales and stock in China. *Energy Policy*, *43*, 17–29. 10.1016/j.enpol.2011.09.063.

[40] World Bank (2019). GDP time series 1960-2019 (Constant 2010 USD, Constant LUC, Current Prices in USD, Current Prices in LUC). https://data.worldbank.org/. Accessed 14 Feb 2020.

[41] Mehlhart, G., Kosińska, I., Baron, Y., Hermann, A. (2017). Assessment of the implementation of the ELV Directive with emphasis on ELVs unknown whereabouts: Report for DG Environment of the European Commission (Oeko-Institut e.V.). Technical report, Oeko-Institut e.V., Freiburg. 10.2779/446025.

