# Supplementary material for: Lifespans of passenger cars in Europe: empirical modelling of fleet turnover dynamics
Source: Eur Transp Res Rev. 2021 Jan 25;13(1):9. doi: 10.1186/s12544-020-00464-0 (PMC7829067; doi:10.1186/s12544-020-00464-0)
Supplement: Supplementary file 1 — Additional file 1 Supporting PDF. 52 pages comprising a detailed description of the methodology used, the assumptions made, the data used, and additional analyses performed. [file 12544_2020_464_MOESM1_ESM.pdf]

RESEARCH

Lifespans of passenger cars in Europe: empirical modelling of fleet turnover dynamics

Maximilian Held*, Nicolas Rosat, Gil Georges, Hermann Pengg, and Konstantinos Boulouchos

*) *Corresponding author:* Maximilian Held, held@lav.mavt.ethz.ch

Abstract

This Supporting Information provides background information to the publication *Lifespans of passenger cars in Europe: empirical modelling of fleet turnover dynamics*.

It contains 32 supporting figures, 5 supporting tables, and additional analyses on 52 pages.

The following sections are structured as follows:

- Appendix A: Background information on the effects of scrappage schemes
- Appendix B: Background information on the choice of a Gaussian distribution to model the imports and exports of used car
- Appendix C: Vehicle stock data
- Appendix D: New registration data
- Appendix E: Imports and exports data
- Appendix F: Cumulative survival probabilities (CSPs)
- Appendix G: Proof of concept of new CSP estimation method
- Appendix H: The simplified CSP estimation method
- Appendix I: Time variability of CSP fitting parameters: Case study for Latvia
- Appendix J: The influence of data resolution
- Appendix K: Overview of all gathered and pre-processed data which are attached as Excel sheets.

Appendix A: Background information on the effects of scrappage schemes.

A scrappage scheme is a monetary renumeration of scrapping an old car, sometimes tied to incentives to buy a new car. The following summary of environmental, economic, and other effects of scrappage schemes is based on a review by Van Wee et al. [1], supplemented by other research findings:

- **Environmental effects:** Due to steady technology advancements, newer cars are usually more fuel-efficient (less CO_2 per vehicle km) and emit less pollutants (non-CO_2 emissions like particulate matter, NO_x, etc.). However, there are also negative emission effects related to scrappage schemes. First, the annual mileage of cars decreases with increasing car age [2, 3, 4]. Newer cars drive on average more per year (higher CO_2 emissions). Second, the 'depreciation period' of the manufacturing emissions of the vehicle is reduced. This is particularly undesirable for vehicles with high manufacturing emissions compared to operational emissions.

  While early scrappage almost always constitutes a benefit with regards to non-CO_2 pollutants, the CO_2 reduction potential of scrappage schemes is disputed [5, 6]. In general, the higher the emissions during manufacturing, the less CO_2 emission reductions can be obtained by replacing an old car with a new one, and thus the older the car should be when scrapped to gain a benefit. Studies about end-of-life vehicle management discuss the corresponding environmental challenges [7, 8].
- **Economic effects:** Scrappage premia were historically widely considered as a policy instrument to boost the economy after recessions [9]. The latest wave of such stimulus programs was established during the aftermath of the financial crisis in 2009, notably in 10 countries of the European Union (Germany, United Kingdom, France, Italy, Spain, Portugal, Austria, the Netherlands, Romania, Spain), in the United States, in Japan, and in Egypt [10], with the German *Abwrackprämie* being the most prominent example [11].

  While (part of) the economic stimulus of scrappage schemes might be due to a shift of sales from future months to the period the scrappage program is put in place [12], there is a positive economic impact in the short-term that is not limited to the issuing country. Economic spillover effects to other countries have been observed in 2009. Maleček & Melcher [12] report direct "effects of the German car scrapping scheme to the Czech real GDP growth [...] between 0.4 and 0.5 percentage points." Since international trade linkages are especially relevant within the European Union, this effect could be observed for other countries as well [12].

  Contrary to the positive economic impacts, the cost-efficiency of such programs as a CO_2 reduction measure are discussed controversially in literature [13, 14, 15]. Li et al. [13] find a rather high CO_2 abatement cost in the range of 100-300 USD per tCO_2 resulting from the United States' Cash-for-Clunkers program in 2009.
- **Other effects:** Since newer cars usually feature a broader range of safety and driver assistance systems, early scrappage of cars has a potentially positive impact on road safety and congestion levels [16].

For scrappage schemes, two criteria can optimize the environmental benefit: (1) the eligibility of old cars being scrapped, and (2) the eligibility of new car models replacing the scrapped cars.

- **Eligibility of old cars for scrapping:** Scrappage schemes should be addressing primarily the oldest part of the fleet [17]. This can be implemented by a threshold for the eligibility of the early retirement program, e.g. by car age or by pollutant emission standards [18]. Accurately setting that age limit (influencing "how much earlier the vehicle was retired because of the program" [19]) is crucial for the design of scrappage schemes, since it has a high impact on its environmental benefit [3, 18]. Estimating the remaining lifespan of a car is an essential, but challenging task to provide guidance for policy-making [17].
- **Eligibility of new cars as a replacement of scrapped cars:** Scrappage premia should be linked to emission standards of new car purchases [14, 15], e.g. using staggered premia aligned with the emission standards of the new cars replacing scrapped ones [19]. As Hahn [17] already pointed out in Hahn1995,

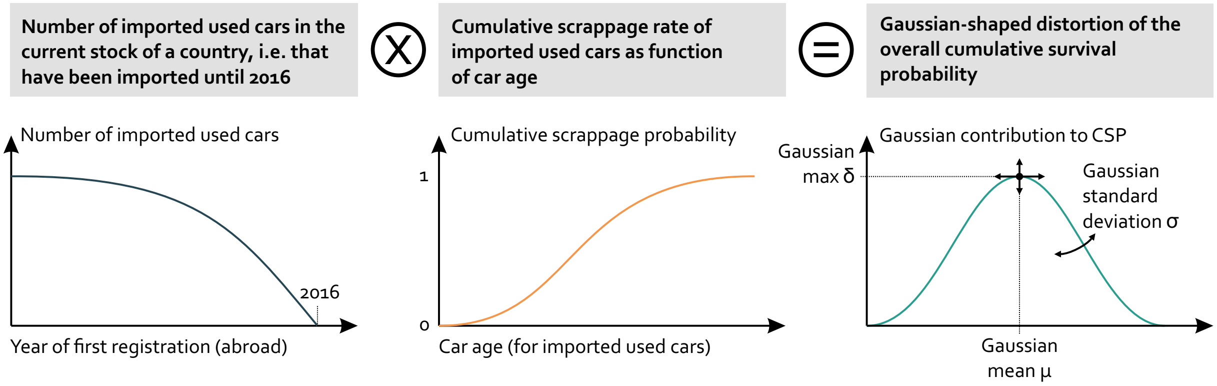

Figure S1 Schematic explanation why a Gaussian distribution is suited to model the impact of imports of used cars for CSPs. Applying the Weibull-shaped scrappage rate to the usually observed number of imported used cars, results in a Gaussian curve.

scrappage schemes are "best viewed as a transitional strategy. Once the relatively dirty vehicles are removed from the fleet, the gains from scrappage are significantly diminished" [17]. In the light of EV-favouring policies, scrappage schemes could be used to 'jump ahead in time' and introduce a new electric era which would have been phased in slowly otherwise. Therefore, Transport & Environment [20] suggests that "scrappage schemes and other support measures should be focused on electric cars; [...] and company's [sic] could enjoy temporary incentives if they go electric in the coming year."

Besides this transformation in car propulsion technology (from combustion to battery-electric), early retirement programs may also have an influence on the share of car categories (e.g. a trend to smaller cars [12]) or even trigger systemic changes in mode split, and therefore lead to further emission reductions [21].

Scrappage schemes are a well-known policy to stimulate the economy after recessions. After the last wave of such programs after the financial crisis in 2009, we are currently amidst the next economy shock triggered by COVID-19. Ideally, *green* economic stimuli of well thought out scrappage schemes could contribute to an economic recovery and to achieve a 1.5 or 2.0°C target at the same time [22, 23].

Appendix B: Background information on the choice of a Gaussian distribution to model the imports and exports of used cars.

Figure S1 shows exemplarily why a Gaussian distribution is suited to model the impact of used cars for CSPs – here for the case of car imports. The age distribution of used cars that have been imported to a country until the year of observation usually follows the left curve in Figure S1. For example, the initial registration year 2014 (cars registered abroad in 2014) contains the (0-)1-yr. old cars imported in 2014, the (1-)2-yr. old cars imported in 2015, and the (2-)3-yr. old cars imported in 2016. These three numbers are summed up for 2014 and likewise for all other years. Due to the high number of imports of 5-15 year-old cars, there is a steep slope of the curve going back from 2016. The shape of this curve was found for most of the countries within our analysis of used car imports data, see Appendix E. Applying the Weibull-shaped scrappage rate (middle curve in Figure S1) to the imported used cars (left curve in Figure S1) results in a Gaussian curve (right curve in Figure S1).

Appendix C: Vehicle stock data.

To compute the survival probability of cars, age-resolved stock data is required. Three different data sources have been considered in this study: the European Automobile Manufacturers' Association, *ACEA*, the United Nations

Economic Commission for Europe, *UNECE*, and national statistics (*NatStat*). The overall data availability is summarized in Table S1. Orange circles indicate the data source that has been chosen to compute the CSPs due to its highest data resolution. Individually gathered data from national statistics (NatStat) clearly provides the highest data resolution. For 17 of 32 countries, NatStat even provides a full resolution of the national stock for cars up to 40 years and older. This is important since the availability of high-resolution stock age distributions was found to be a critical bottleneck for an accurate estimation of the CSPs of the observed countries.

Table S1 Age-resolved stock data: ACEA [24] and UNECE [25] data are for 2016. Eurostat [26] provides the same data as UNECE. Data from national statistics (NatStat) stem from national statistics offices, ministries of interior or transport, national automotive associations, national automotive companies, etc. For each country, orange circles indicate the data source that has been chosen for further analyses due to its highest resolution. Age bins with aggregate numbers for multiple years are indicated by squared brackets. *Abbreviations:* ACEA = European Automobile Manufacturers' Association, UNECE = United Nations Economic Commission for Europe, NatStat = National Statistics.

	ACEA [24]	UNECE [25]	NatStat (age bins)	NatStat (year)	NatStat (ref)
AUT	●	●	● (orange) (1-24, 25+)	2016	[27]
BEL	●	●	● (orange) (1-121)	2018	[28]
BGR	○	○	○	—	—
CHE	●	●	● (orange) (1-121)	2016	[29]
CYP	○	● (orange)	○	—	—
CZE	●	◐	● (orange) (1-70, 70+)	2014	[30]
DEU	●	●	● (orange) (1-37, 38+)	2016	[31]
DNK	●	◐	● (orange) (1-25, 26+)	2016	[32]
ESP	●	●	● (orange) (1-116)	2016	[33]
EST	●	●	● (orange) (1-121)	2016	[34]
FIN	●	●	● (orange) (1-121)	2018	[35]
FRA	● (orange)	◐	○	—	—
GBR	●	●	● (orange) (1-47, 48+)	2016	[36]
GRC	●	○	● (orange) (1-43, 44+)	2018	[37]
HRV	●	● (orange)	◐ ([1-5], [6-10], 11+)	2017	[38]
HUN	●	●	● (orange) (1-121)	2017	[39]
IRL	●	◐	● (orange) (1-17, 18+)	2016	[40]
ISL	○	○	● (orange) (1-121)	2018	[41]
ITA	●	◐	● (orange) (1-20, 20+)	2016	[42]
LIE	○	●	● (orange) (1-121)	2016	[29]
LTU	●	●	● (orange) (1-121)	2020	[43]
LUX	●	◐	● (orange) (1-121)	2017	[44]
LVA	●	●	● (orange) (1-114)	2016	[45]
MLT	○	●	◐ (orange) (1, [2-4], [5-9], [10-19], [20-29], [30-39], [40-49], [50-59], [60-69], [70-79], 80+)	2016	[46]
NLD	●	●	● (orange) (1-121)	2016	[47]
NOR	●	●	● (orange) (1-121)	2016	[48]
POL	●	●	● (orange) (1-20, 21+)	2016	[49]
PRT	●	●	● (orange) (1-70, 70+)	2016	[50]
ROU	● (orange)	○	◐ ([1-2], [3-5], [6-10], 11+)	2016	[51]
SVK	● (orange)	○	◐ ([1-2], [3-5], [6-10], 11+)	2016	[52]
SVN	●	● (orange)	○	—	—
SWE	●	●	● (orange) (1-18, 19+)	2016	[53]

Legend (Definition of data availability-circles above):

	●	◐	○
ACEA	1-10, 11+	-	N/A
UNECE	1-2, 3-5, 6-10, 11-20, 21+	1-2, 3-5, 6-10, 11+	N/A
NatStat	continuous data	discontinuous data in age bins	N/A

ACEA annually reports age-resolved stock data for 27 countries of the European Economic Area, EEA (all EEA countries except BGR, CYP, ISL, LIE, and MLT). [24] However, only the first ten years are resolved continuously. All cars older than 10 years are lumped in an 'overflow bin' (11+).

UNECE and Eurostat provide the same age-resolved stock data for 27 countries (all countries except BGR, GRC, ISL, ROU, and SVK) [25, 26]. The data is reported in coarser age bins than the data from ACEA, but covers a wider range of car ages for many countries (age bins: 1-2, 3-5, 6-10, 11-20, 21+). Note that "GBR"

refers to Great Britain (Northern Ireland not included), while for the rest of this study GBR refers to the United Kingdom. The data for Finland does not include vehicles in Åland.

National statistics have been gathered individually for 28 countries (all countries except BGR, CYP, FRA, and SVN) from national statistics offices, ministries of interior or transport, national automotive associations, national automotive companies, and others. If no age-resolved stock data was available for 2016, we chose the closest year with available data. To account for different total stock sizes, we corrected the total stock numbers by the ratio of total stock in the respective year and 2016, while keeping the shares of cars per age bin constant. Where the respective NatStat source did not provide the total stock size in 2016 (BEL, CZE, LUX), we applied an analogous correction to the 2016 totals of UNECE. We assume that the age distribution usually does not change drastically within one or two years (maximum deviation is two years for 2014 and 2018 data). This has been confirmed by a case study of Latvia in Appendix I and the study of Oguchi & Fuse [54]. The following list states other notes on the data and assumptions for processing it:

- EST: Data for Estonia contains vehicles with suspended registration.
- FIN: Data for Finland does not include vehicles from Åland's separate register.
- GRC: The data contains the "year of first circulation internationally" and the "year of first circulation in Greece". Where the "year of first circulation internationally" is "without indication" in the original data set, we assume that it is similar to the "year of first circulation in Greece". Hence, we assume that these cars are new. Where the "year of first circulation internationally" is *before* a certain year in the original data set, we set it to this year. We do this for simplicity, since these cases represent considerably less than 1% of the total cars registered in Greece in the respective year.

In the following, we document the pre-processing procedure of vehicle stock data according to the available data resolution for NatStat data:

- Stock data with continuous age distribution (single years) and no overflow bin (full resolution)
- Stock data with continuous age distribution and overflow bin
- Stock data with discontinuous age distribution (multiple years lumped together in one age bracket) and overflow bin

The depicted graphs in Figures S2-S6 refer to the chosen data sources with the highest resolution (orange circles in Figure S1).

Stock data with continuous age distribution and no overflow bin (full resolution).
Figures S2 and S3 show the age distribution for all 13 countries with a full resolution (no overflow bin). This raw data already shows the inhomogeneity between countries. While a first group (Figure S2) covers countries from Northern and Central Europe, a second group (Figure S3) consists of Eastern and Southern European countries, with the exception of Iceland, which has high fluctuations due to its relatively small car market. For the first group, on average only 39.4% of all cars are equal to or older than 10 years. For the second group, on average 66.1% of all cars are equal to or older than 10 years.

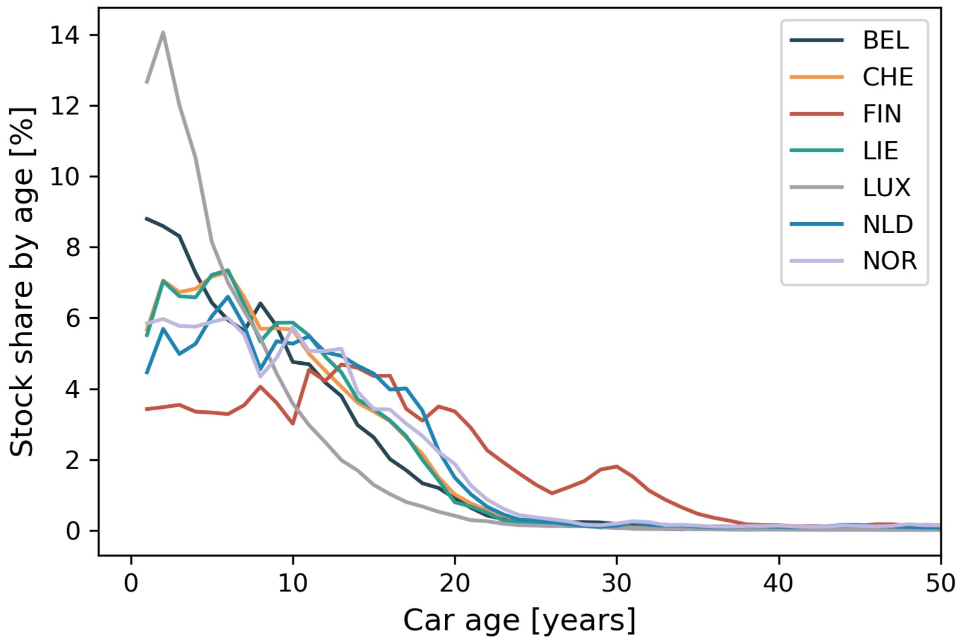

Figure S2 Raw stock data for selected countries with full data availability (continuous resolution) and a peak for younger cars. NatStat data sources are documented in Table S1.

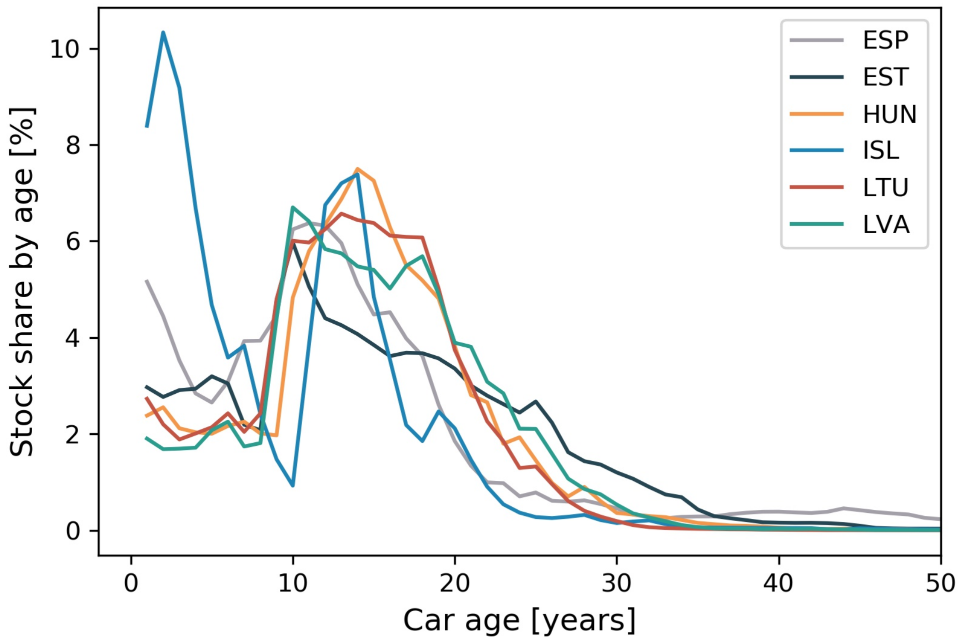

Figure S3 Raw stock data for selected countries with full data availability (continuous resolution) and a peak for older cars. NatStat data sources are documented in Table S1.

Stock data with continuous age distribution and overflow bin.
For all data sets with a continuous resolution up to a certain age and an overflow bin above (e.g. "older than 10 years"), we distribute the number of cars in the overflow age bin according to the value in the last fully resolved age bin. We sustain this value until the overflow bin is "depleted". *Example:* Assuming there are 50'000 ten-year-old cars in a country and the raw data reports "175'000 cars older than 10 years", we distribute the 175'000 cars as follows: 50'000 eleven-year-old cars, 50'000 twelve-year-old cars, 50'000 13-year-old cars and 25'000 14-year-old cars.

The effect of sustaining the overflow bins can be seen best for Poland in Figure S5. In reality, the distributions for higher car ages will rather follow the tails of the curves shown in Figure S2 and S3 – with a steady decrease instead of a sustained constant value and then an abrupt drop. However, since we do not have any knowledge about the nature of this distribution tail for the countries with an overflow bin, we stick as close to the raw data as possible and avoid any misleading interpretation of the missing data by sustaining the overflow bin constantly as described.

There is one anomaly: the overflow bin of the Czech Republic ("older than 70 years") is so large that the age bins 71 to 121 are not sufficient to distribute the number of cars in the overflow bin when sustaining the same number of cars as in the 70 yr.-old age bin. This is due to the low number of 70-year-old cars. To solve this issue, we sustain the average number of cars in the last 5 age bins (66-70) until the overflow bin is depleted. Overall, this has a negligible influence on the CSP due to the very low number of such old cars compared to the total stock.

Figures S4 and S5 show the age distribution for all countries in this data resolution category – separated into countries with a high number of young/old cars. We see the same divide as for the first resolution category: Northern and Central European countries feature a rather steady decrease with car age, while Eastern and Southern European countries have a pronounced peak at older ages.

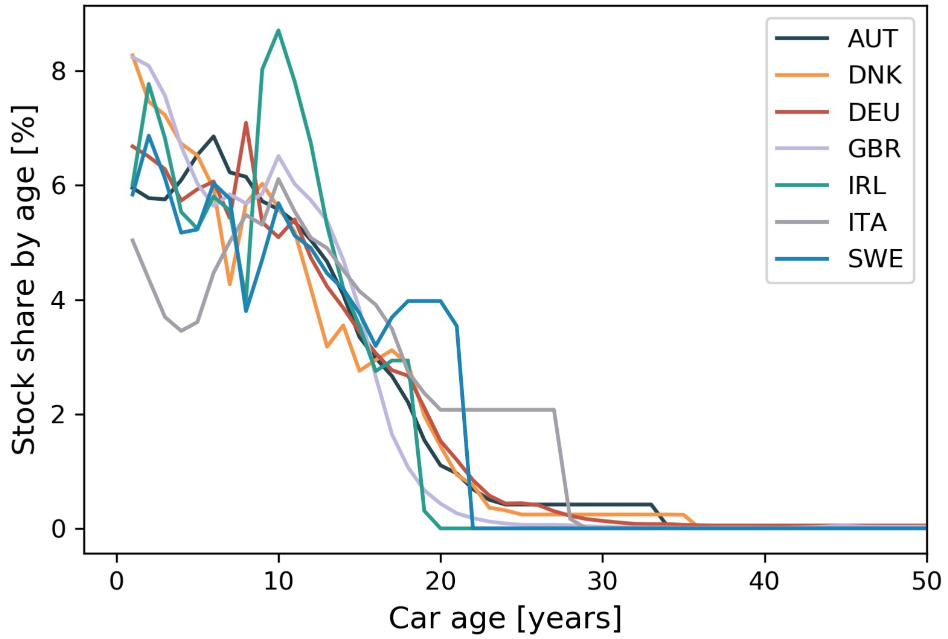

Figure S4 Raw stock data for selected countries with high data availability (continuous resolution and overflow bin) and a peak for younger cars. Data sources are documented in Table S1.

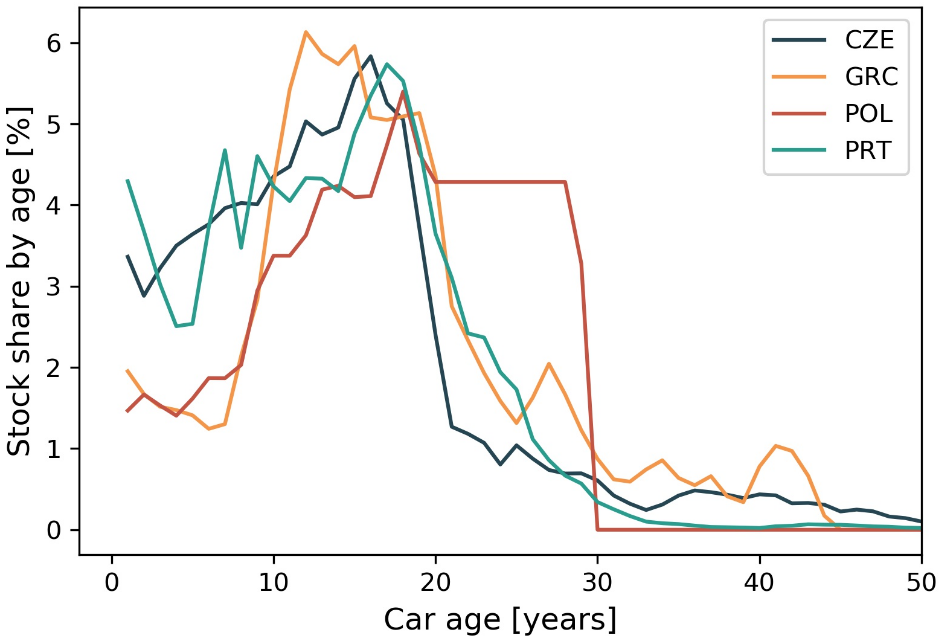

Figure S5 Raw stock data for selected countries with high data availability (continuous resolution and overflow bin) and a peak for older cars. Data sources are documented in Table S1.

Stock data with discontinuous age distribution and overflow bin.

For all data sets with a discontinuous resolution up to a certain age and an overflow bin above (e.g. "1-5, 6-9, 10-15, older than 15 years"), we distribute the number of cars in each age bin uniformly among all ages covered by the bin. The overflow bins are sustained as described earlier. Figure S6 shows the age distribution for all countries in this data resolution category. They are located in Eastern or Southern Europe. We observe a peak for older cars, as for the other categories.

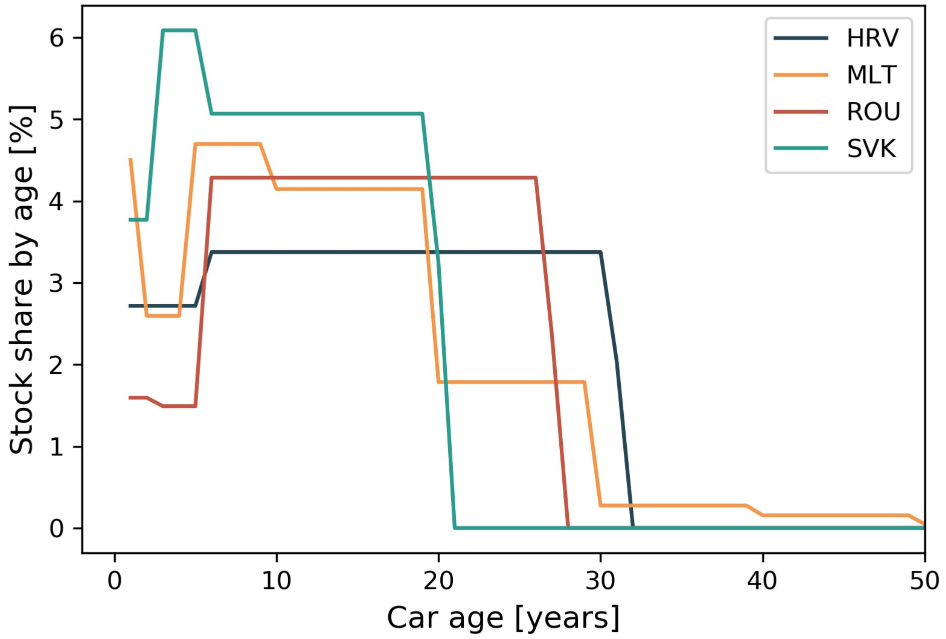

Figure S6 Raw stock data for selected countries with low data availability (discontinuous resolution and overflow bin) and a peak for older cars. Data sources are documented in Table S1.

Overview over stock data from all data sources available.

Figures S7-S9 show all car stock age distributions available – for all three data sources documented in Table S1. While ACEA and UNECE already provide a rich data set of many countries with a decent data resolution, the benefit of the compilation of national statistics lies in a higher data resolution for almost all countries. High-resolution stock data sets are the basis for an accurate determination of the survival probabilities of cars.

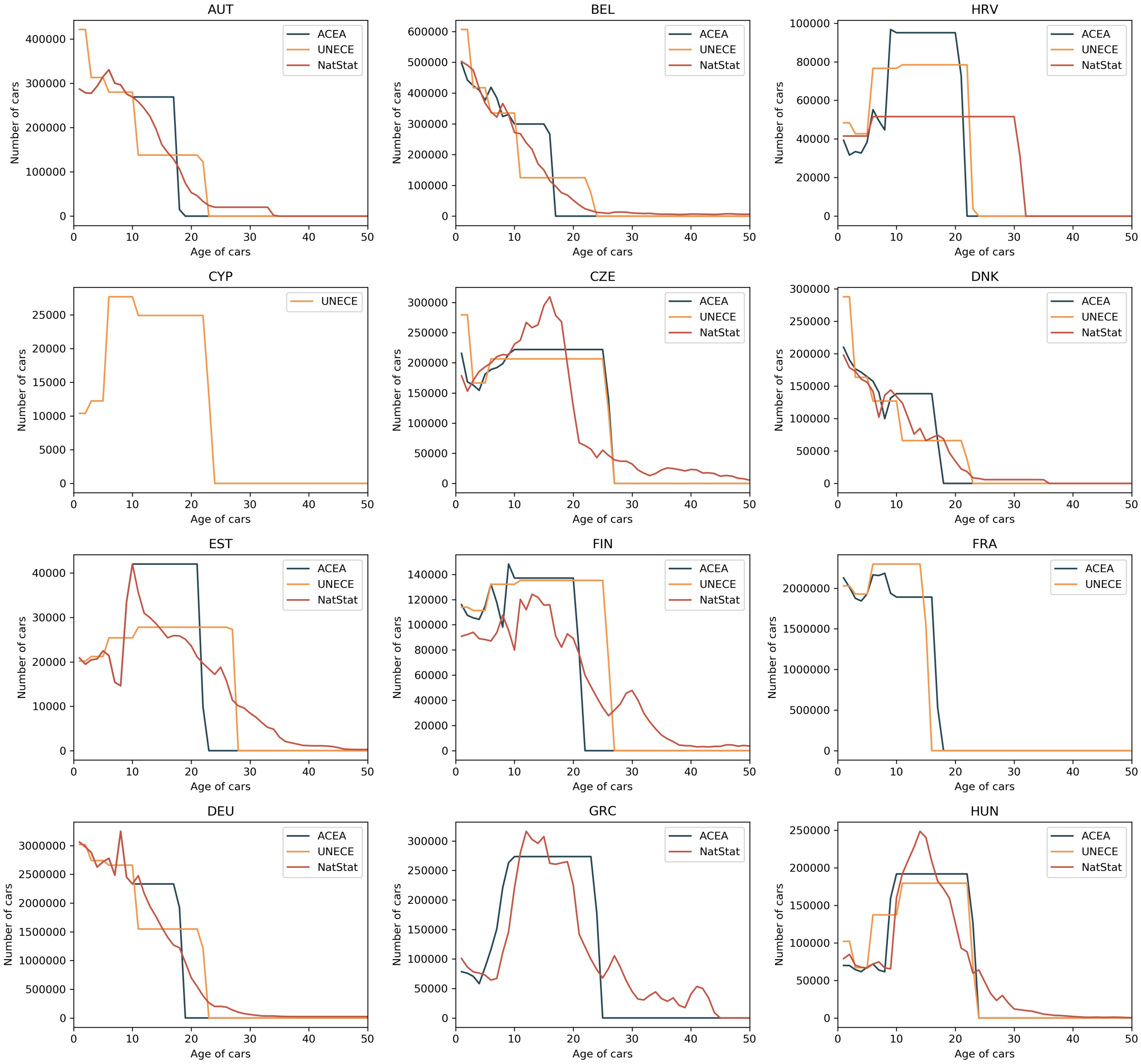

Figure S7 Raw stock data per country for all available data sources (1/3): ACEA [24], UNECE [25], and national statistics (NatStat). Data sources are documented in Table S1.

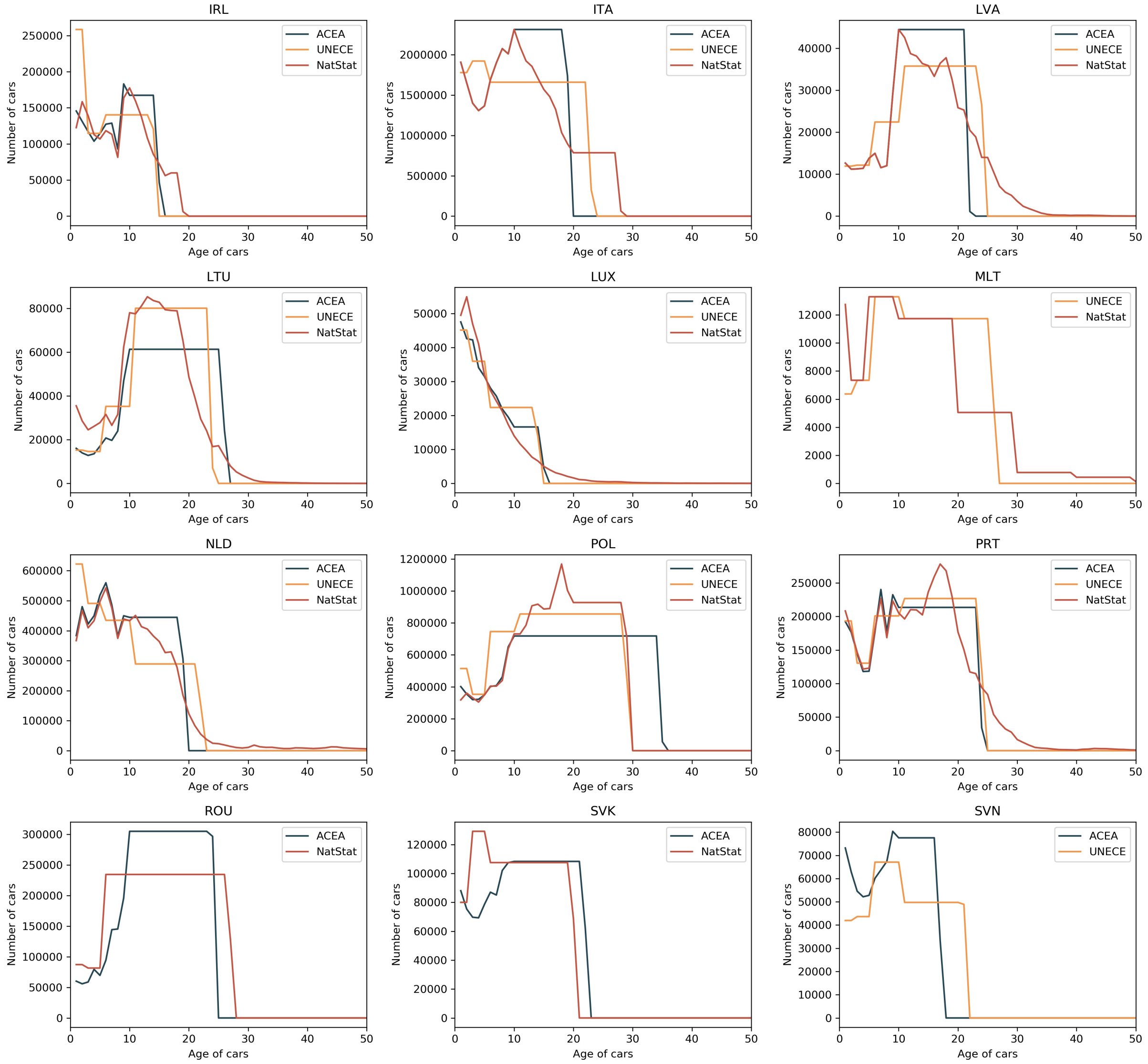

Figure S8 Raw stock data per country for all available data sources (2/3): ACEA [24], UNECE [25], and national statistics (NatStat). Data sources are documented in Table S1.

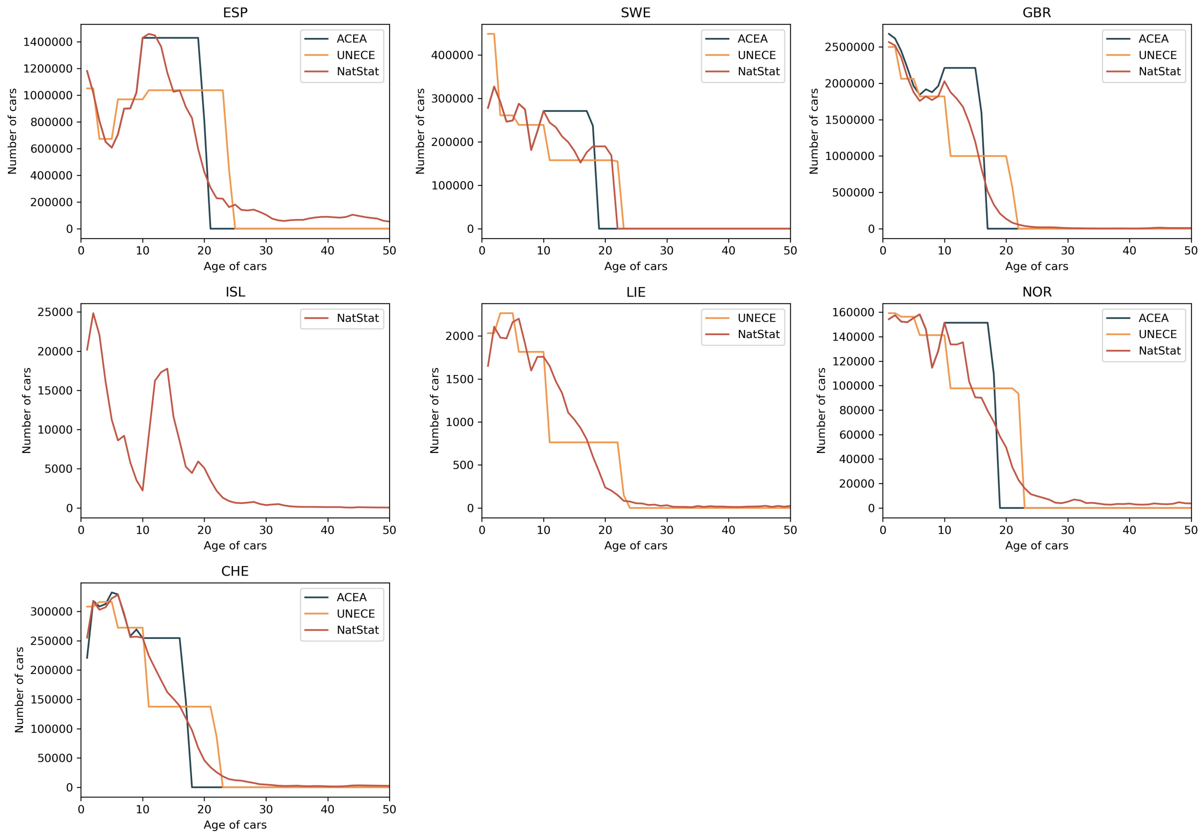

Figure S9 Raw stock data per country for all available data sources (3/3): ACEA [24], UNECE [25], and national statistics (NatStat). Data sources are documented in Table S1.

Appendix D: New registration data.

In addition to vehicle stock data, the computation of survival probabilities also requires knowledge on the number of new vehicle registrations per year. Four different data sources have been considered in this study: the European Automobile Manufacturers' Association, *ACEA*, the United Nations Economic Commission for Europe, *UNECE*, national statistics (*NatStat*), and the European Statistical Office (*Eurostat*). The overall data availability is summarized in Table S2. As is the case for the stock data, the NatStat data for new registrations provides a benefit in terms of longer historical time series.

While UNECE overall provides longer time series than ACEA, its data suffers from a considerable number of missing entries, which is not the case for ACEA. Eurostat and NatStat offer the longest time series for many countries. In addition, NatStat data rarely has any missing entries and therefore was most often chosen for further processing within this study, see orange circles in Table S2.

For many countries, the registration data reported by different data sources are not congruent. This is mainly due to the fact that some data sets sometimes contain both the new registrations of *new* cars and the new registrations of *used* imported cars (mainly UNECE and Eurostat), while others always provide data only for new registrations of *new* cars (mainly ACEA and NatStat). Furthermore, different original data sources[1] lead to additional (but usually small) deviations. In the following, we list all available information on the different data sets:

- ACEA:
  - "Data on new registrations only refers to newly registered vehicles, therefore it doesn't take into account used or second-hand vehicles." (private correspondence with Ms. Francesca Piazza, ACEA)
  - New registration data for Romania refers to car sales [55].
- UNECE:
  - "The data should refer to registration of new vehicles, so including the second hand imports. Unfortunately there is little harmonisation on this, both in the UNECE region and even within EU countries. [...] This lack of harmonisation (and lack of data on second hand imports in general) is a recognised problem and has been discussed. [...] So for now, unfortunately it's difficult to make comparisons across countries." (private correspondence with Mr. Alex Blackburn, UNECE)
  - GRC, HUN: Data "includes both new vehicles and used vehicles from abroad" [56].
  - LVA: Data "includes vehicles that have been manufactured in the indicated or previous year only" [56].
  - LTU: Data "includes new and re-registered vehicles" [56].
  - GBR: Data refers to Great Britain, not UK [56].
- NatStat:
  - Unless stated otherwise, these numbers are very consistent with ACEA data, which means that they are likely to represent registrations of new cars only (no imported cars). That is also stated explicitly for a number of countries, including HUN, LVA, POL, NOR, and DEU. For Germany, for example, the methodological documentation of their statistics says: "Eine Neuzulassung ist die erstmalige Registrierung eines fabrikneuen Fahrzeugs mit Kennzeichen in Deutschland. Fahrzeuge, die bereits im In- oder Ausland zugelassen waren, fallen nicht darunter" [57]. [Translation from Google Translate: "A new registration is the first registration of a brand new vehicle with license plate in Germany. Vehicles that have already been registered in Germany or abroad are not included."]
  - Exceptions: Data for ISL and ESP includes imported used cars.

---

[1]Note that UNECE, ACEA and Eurostat only collect data from national statistics.

– Data for Great Britain is available for 2001-2018, for the UK only for 2015-2018. Hence, we took the average ratio of UK/GB numbers and applied that factor to the GB-values for 2001-2014 in order to get the UK values. Note that the Northern Irish cars (difference between UK and GB) amount only to 2.2% of UK cars, wherefore the potential error due to this correction is negligible.

- Eurostat:
  – Data is similar to UNECE, but with an overall higher data availability.
  – Private correspondence with the Eurostat User Support revealed: "In general you cannot compare Eurostat data to other sources, as mostly different methodologies are used. Eurostat has either a directive or regulation setting up for the current 27 member states on how to deliver data. You can find this directive here: `https://eur-lex.europa.eu/legal-content/EN/TXT/PDF/?uri=CELEX:32012R0070&from=EN`."

We will discuss the impact of these different data sets later on.

Table S2 New registration data from ACEA [55], UNECE [56], national statistics (NatStat) and Eurostat [58]. Data from national statistics (NatStat) stem from national statistics offices, ministries of interior or transport, national automotive associations, national automotive companies, etc. For each country, orange circles indicate the data source that has been chosen for further analyses due to its highest resolution.
Abbreviations: ACEA = European Automobile Manufacturers' Association, UNECE = United Nations Economic Commission for Europe, NatStat = National Statistics.
Footnotes and further comments are documented in Table S3.

	ACEA [55]	UNECE (missing years [\xxxx] interpolated) [56]	NatStat	NatStat (ref.)	Eurostat (missing years [\xxxx] interpolated, 1971-1974 and 1976-1978 missing for all cty.) [58]
AUT	◑	◑ [\2005]	◑	[59]	◕ [\2005]
BEL	◑	◑ [\1997]	◑	[60]	● [a]
BGR	◔	◑ [\2013-2015]	○	—	◑
CHE	◑	◑	◕	[61]	◕
CYP	○	◑	◔	[62]	◑
CZE	◔	◔	◑	[63]	◔
DEU	◑	◑	● [b]	[64]	● [\1991]
DNK	◑	◑	◑	[65]	● [\2003-2005, 2009-2012]
ESP	◑	◑ [\2003-2005]	◑	[66]	● [\2003-2005]
EST	◔	◑ [\2005]	◑	[67]	◑ [\2005]
FIN	◑	◑ [\2005]	●	[68]	◕ [\2005]
FRA	◑	◑ [\2005]	◑	[69]	● [\2005]
GBR	◑	◑ [\2005, 2013, 2014]	◔ [c]	[70]	● [\2005-2007, 2012-2014]
GRC	◑	◑ [\2004-2008]	◕	[71]	●
HRV	◔	◑	◔	[72]	● [\1979, 1981-1984, 1986-1989]
HUN	◔	◑ [\2005]	◑	[73]	◑ [\1991, 2005, 2009]
IRL	◑	◑ [\2005, 2006, 2008]	●	[74]	◕ [\1981-1984, 1986-1989]
ISL	◑	◑ [\2006, 2007]	◕ [d]	[75]	◑
ITA	◑	◑ [\2003-2007, 2014, 2015]	●	[76]	● [\2014]
LIE	○	◔	◔	[77]	◔ [\2005]
LTU	◔	◑	◔	[e]	◑
LUX	◑	◑	● [f]	[78]	● [\1995-1997]
LVA	◔	◑	◑	[79]	◑
MLT	○	◑ [\2003, 2004]	◔	[80]	◑ [\1991-1994, 2003, 2004]
NLD	◑	◑ [\1995, 1996]	●	[81]	● [\1995, 1996]
NOR	◑	◑ [\2001-2003, 2005]	◔	[82]	◕ [\2001-2003, 2005]
POL	◔	◑	◔	[83]	◑
PRT	◑	◑ [\2003-2008, 2013]	●	[g]	● [\1987, 2003-2008, 2013]
ROU	◔	◑ [\2000, 2005, 2014, 2016]	◔	[84]	◑ [\2000-2003, 2014, 2016]
SVK	◔	◑ [\1994, 1995, 2005, 2006]	◔	[85]	◑ [\1994, 1995, 2005, 2006]
SVN	◔	◑	◑	[86]	◑
SWE	◑	◑	◑ [h]	[87]	◕

Legend (Definition of data availability-circles above):

●	◕	◑	◔	○
≤ 197x-2016	198x-2016	199x-2016	200x-2016	N/A

Table S3 Footnotes and comments for Table S2, *Abbreviations:* UK = United Kingdom, GB = Great Britain.

Footnotes:

Footnote	Description
a	1997 missing, erroneous value for 1991 manually corrected.
b	1960-2010 in 5-year steps linearly interpolated.
c	Ratio of UK-to-GB figures for 2001-2014 applied for years where only GB data was available.
d	Data includes imports of used cars.
e	- 2004-2011: sum of new and used car registrations from Statistics Lithuania [88], corrected by the average ratio between new and used cars from 2006-2008 from Vanherle & Vergeer [89].<br>- 2012-2016 from Statistics Lithuania [88].
f	1976, 1977 missing.
g	1969-2012 and 2014-2016 from Pordata [90], 2013 values for "ligeiros e pesados" from Statistics Portugal [91].
h	Imports assumed to be used cars.

Further comments on chosen data sets (for countries where two data sets have been merged):

Country	Years: Source
DEU	1960-1978: NatStat, 1979-2016: Eurostat
DNK	1970-1991: Eurostat, 1992-2016: NatStat
FRA	1970-1989: Eurostat, 1990-2016: NatStat
GBR	1970-1989: Eurostat, 1990-2016: ACEA

Figures S10-S12 show all time series of new car registrations available.

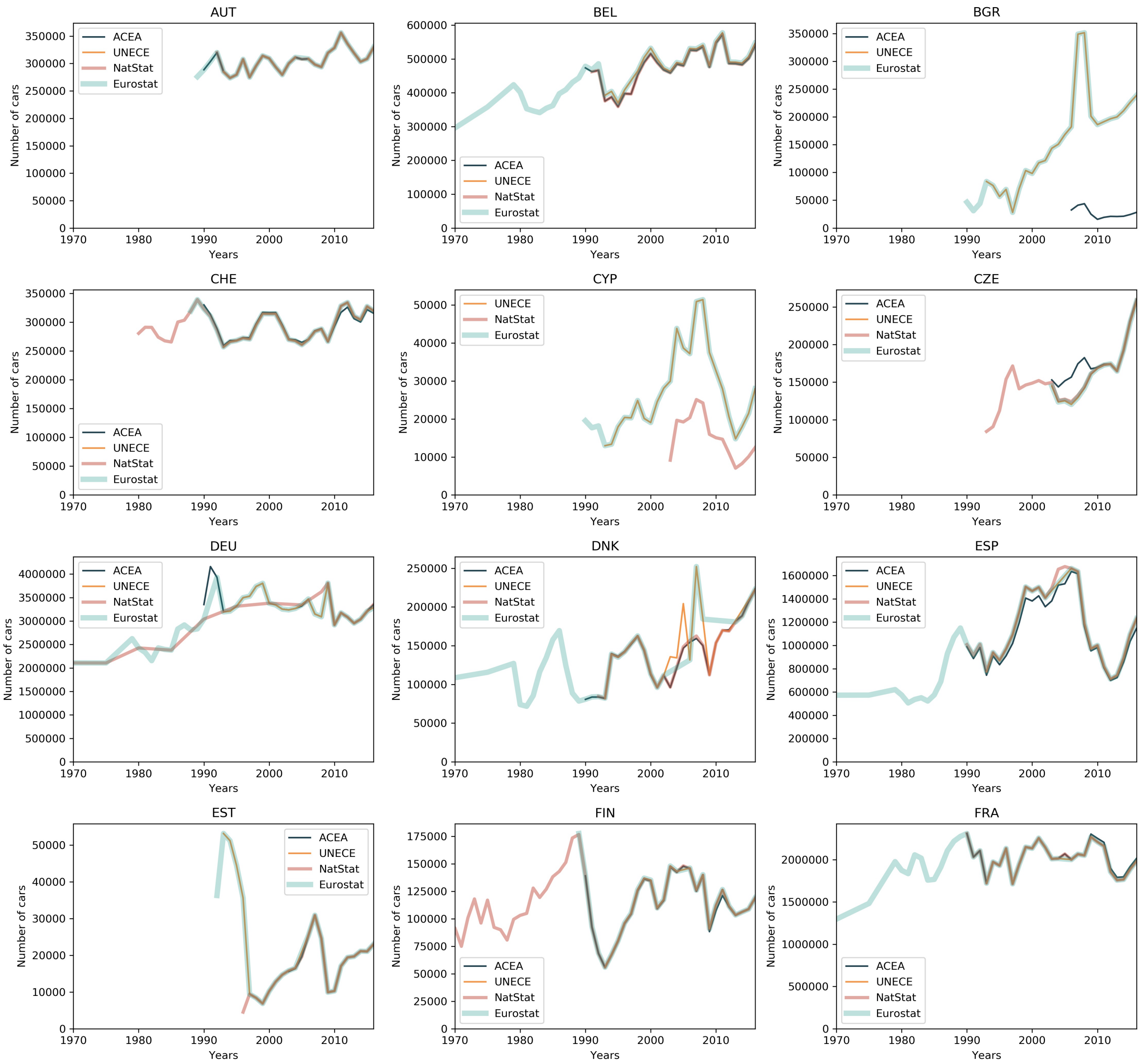

Figure S10 Raw data of new car registrations per country for all available data sources (1/3): ACEA [55], UNECE [56], national statistics (NatStat), and Eurostat [58]. Data sources are documented in Table S2.

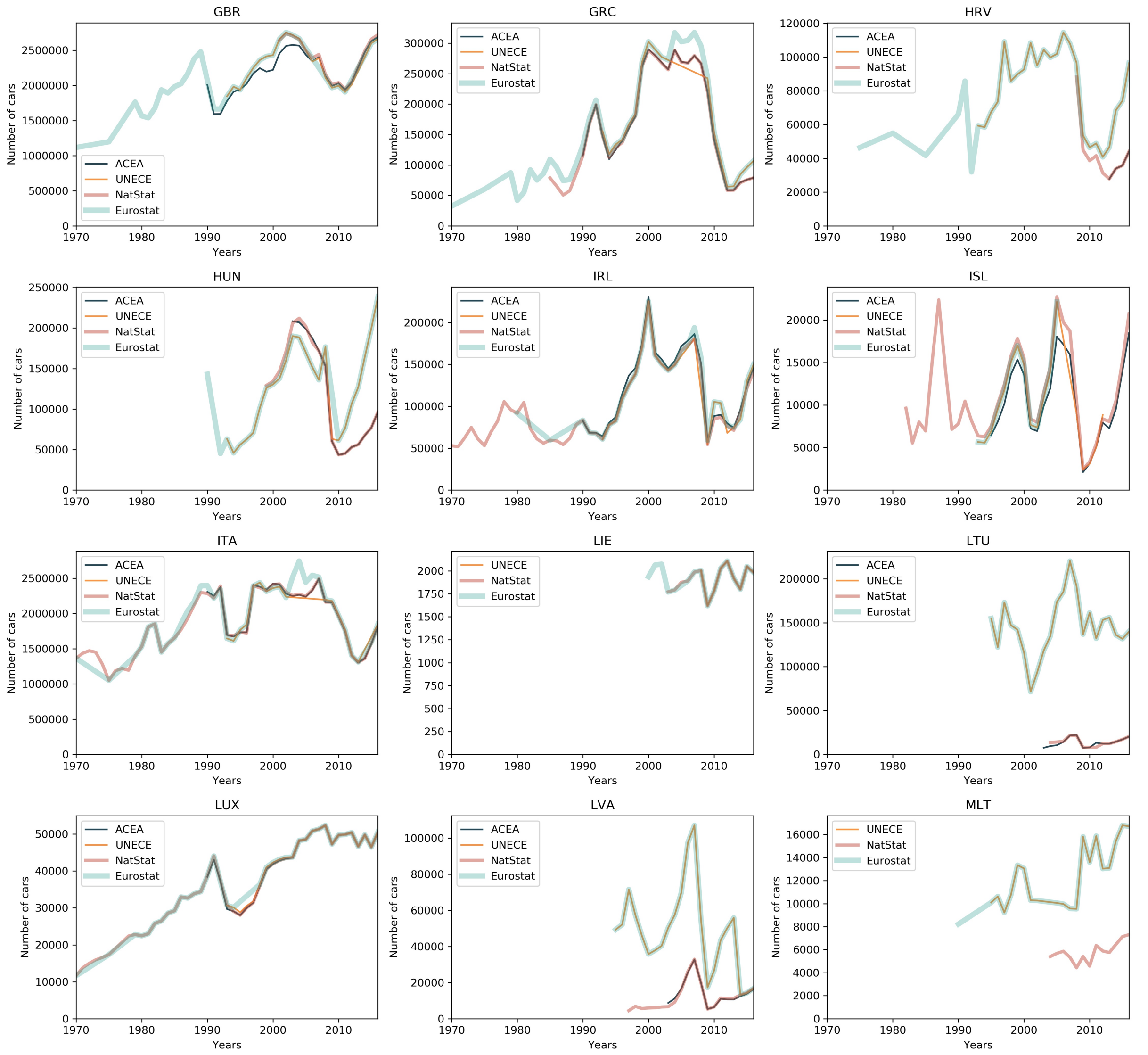

Figure S11 Raw data of new car registrations per country for all available data sources (2/3): ACEA [55], UNECE [56], national statistics (NatStat), and Eurostat [58]. Data sources are documented in Table S2.

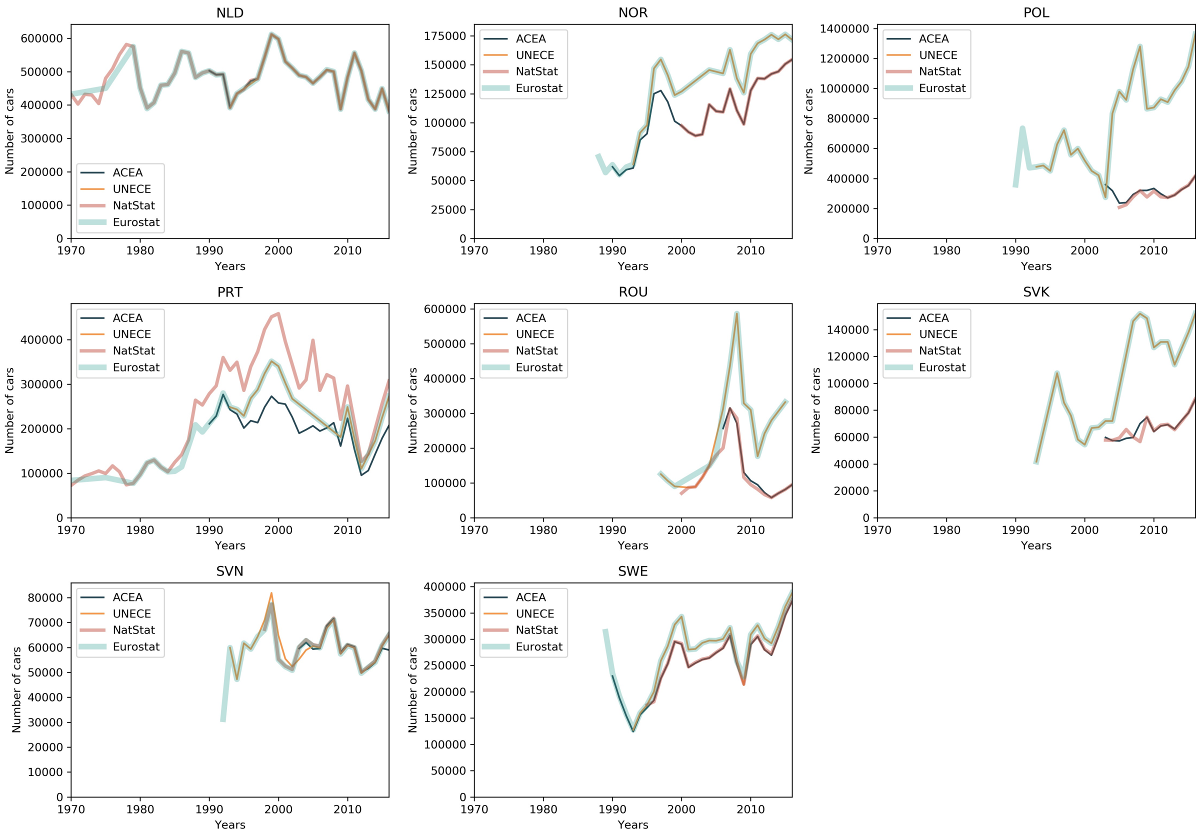

Figure S12 Raw data of new car registrations per country for all available data sources (3/3): ACEA [55], UNECE [56], national statistics (NatStat), and Eurostat [58]. Data sources are documented in Table S2.

As discussed earlier, different data sources show considerable deviations in the provided figures. In the following we analyse the potential origins of the data, in particular for which data sources used vehicles are included and for which they are not:

1 AUT, BEL, FIN, FRA, GRC, IRL, ITA, LUX, NLD, SVN, LIE, CHE: Since ACEA only reports new registrations of new cars (w/o imported used cars) and since all available data sources are very similar for these countries, either the number of imported used cars is (close to) zero, or the other data providers also report only new car registrations.

2 BGR, HRV, CYP, LVA, LVA, LTU, MLT, POL, ROU, SVK, SWE, NOR: While ACEA and NatStat report new car registrations only, the values reported by UNECE/Eurostat seem to include used car registrations. The UNECE data even provides footnotes for some countries for which this is the case.

3 ESP, ISL, PRT: NatStat data includes new and imported used cars. Note that for ISL, all data sources are a bit scattered. Hence, the deviations might also be statistical errors.

4 GBR: NatStat seems to include imported used cars as well. From private correspondence with the UK's Vehicle Statistics Office (`vehicles.stats@dft.gov.uk`), we got the following indication: "If vehicles are imported from other European countries then they may well appear as new registrations rather than imports."

5 CZE: ACEA values differ between 2003 and 2008. Private correspondence with ACEA revealed that "during this period, we were forced to change sources of information for the Czech Republic. The difference in figures between 2003 and 2008 is due to this change."

6 DEU: Differences within the ACEA and NatStat data set arise from different original data sources, as private correspondence with ACEA revealed: "ACEA source for German data is VDA (`https://www.vda.de/en.html`), not KBA", whereby our NatStat data is sourced from KBA (Kraftfahrtbundesamt) which is the German Federal Motor Transport Authority.

7 DNK, HUN: The outliers in the UNECE/Eurostat data for 2005 and 2007 cannot be explained currently but they seem to be errors in the data set. Since UNECE/Eurostat data includes both new and used (imported) cars, their numbers should always be higher than those of NatStat/ACEA, which is not the case.

8 EST: The high UNECE values are an error in the data set. According to Estonian statistics the UNECE values for 1993-1996 are exactly the same as the *stock* of cars, not the new registrations.[2]

Based on the analysis above, we chose one data set for each country for further processing in this study. The chosen data sets are highlighted in Table S2 with orange colour. Still, only short time series are available for several of the studied countries. For those, we compute the number of new registrations per capita using population data from end of 2016 [92]. Since this indicator is rather constant for most countries with saturated markets (with fluctuations around a mean due to fluctuating economic activity), we use the average new registrations per capita over the 'oldest' 10 years of the available time series to backcast missing data via population statistics (which offers a high availability of long historical time series). Figures S13-S15 show the number of new registrations per capita and the average over the whole time series, including the mean deviation from this constant average.

[2] The stock numbers are available via `http://andmebaas.stat.ee/?lang=en`, Table "TS322: First registrations of vehicles (months)". This erroneous data has been deleted.

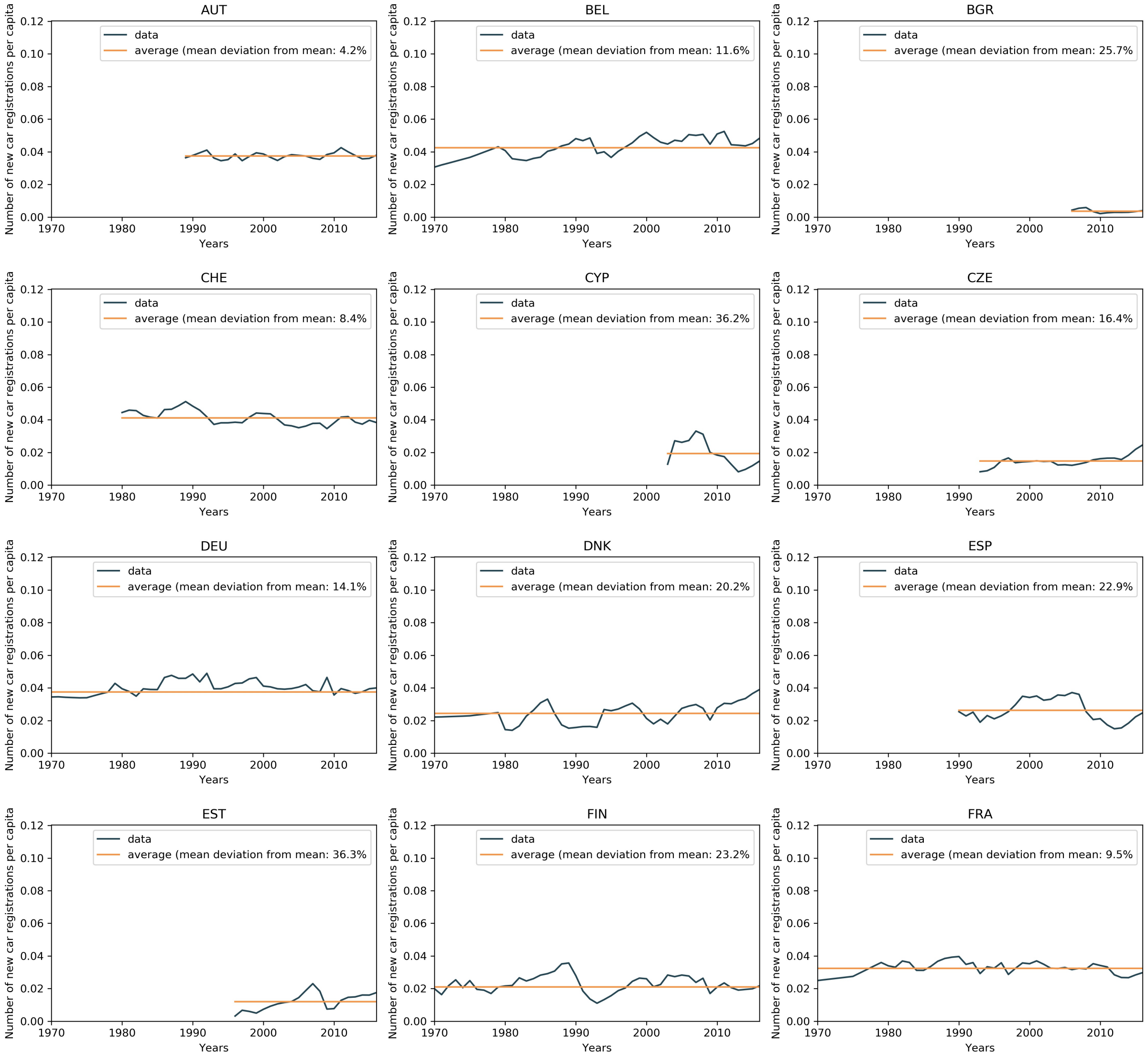

Figure S13 New car registrations per capita for all 32 countries for the data source with the highest resolution (1/3).

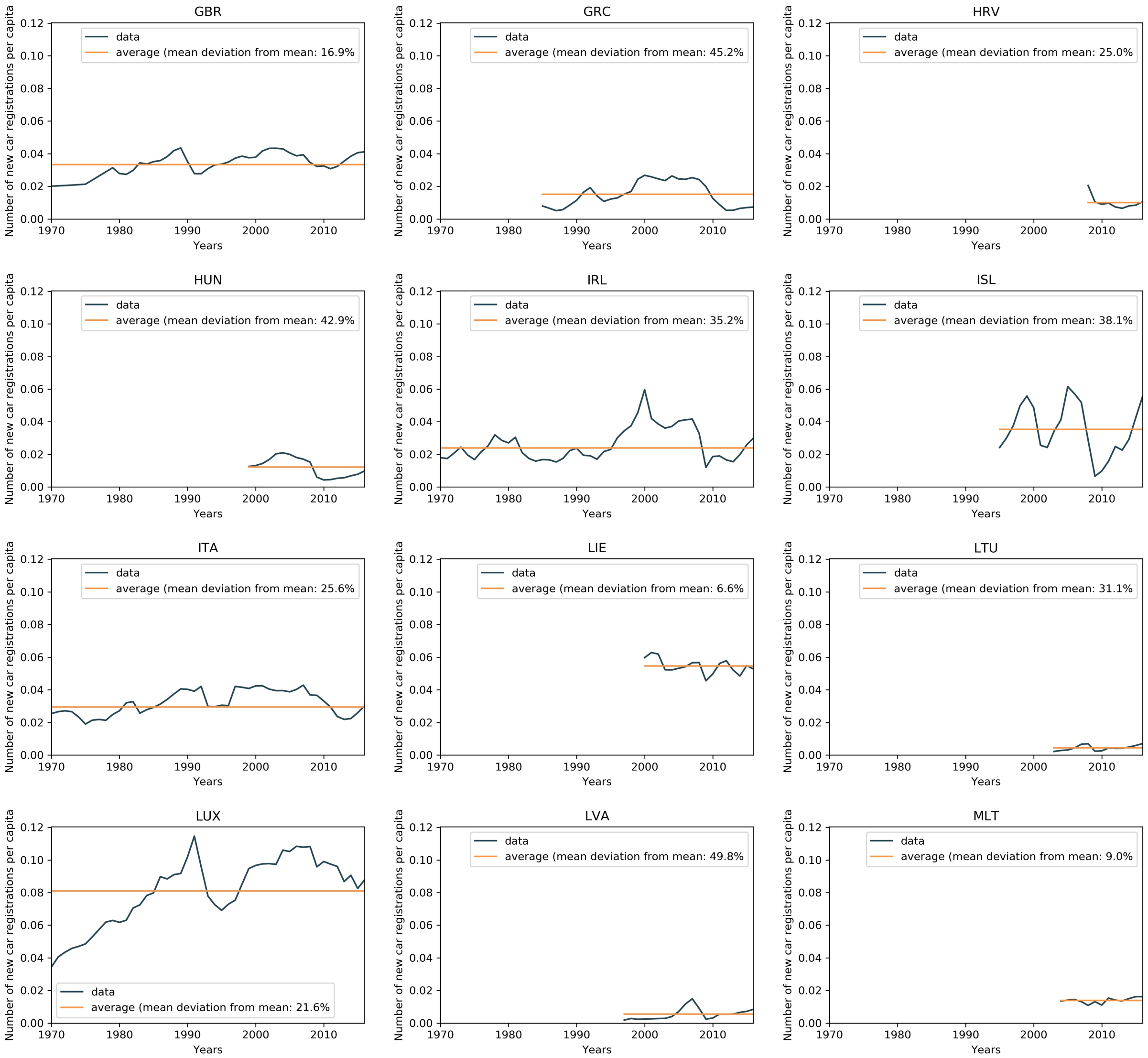

Figure S14 New car registrations per capita for all 32 countries for the data source with the highest resolution (2/3).

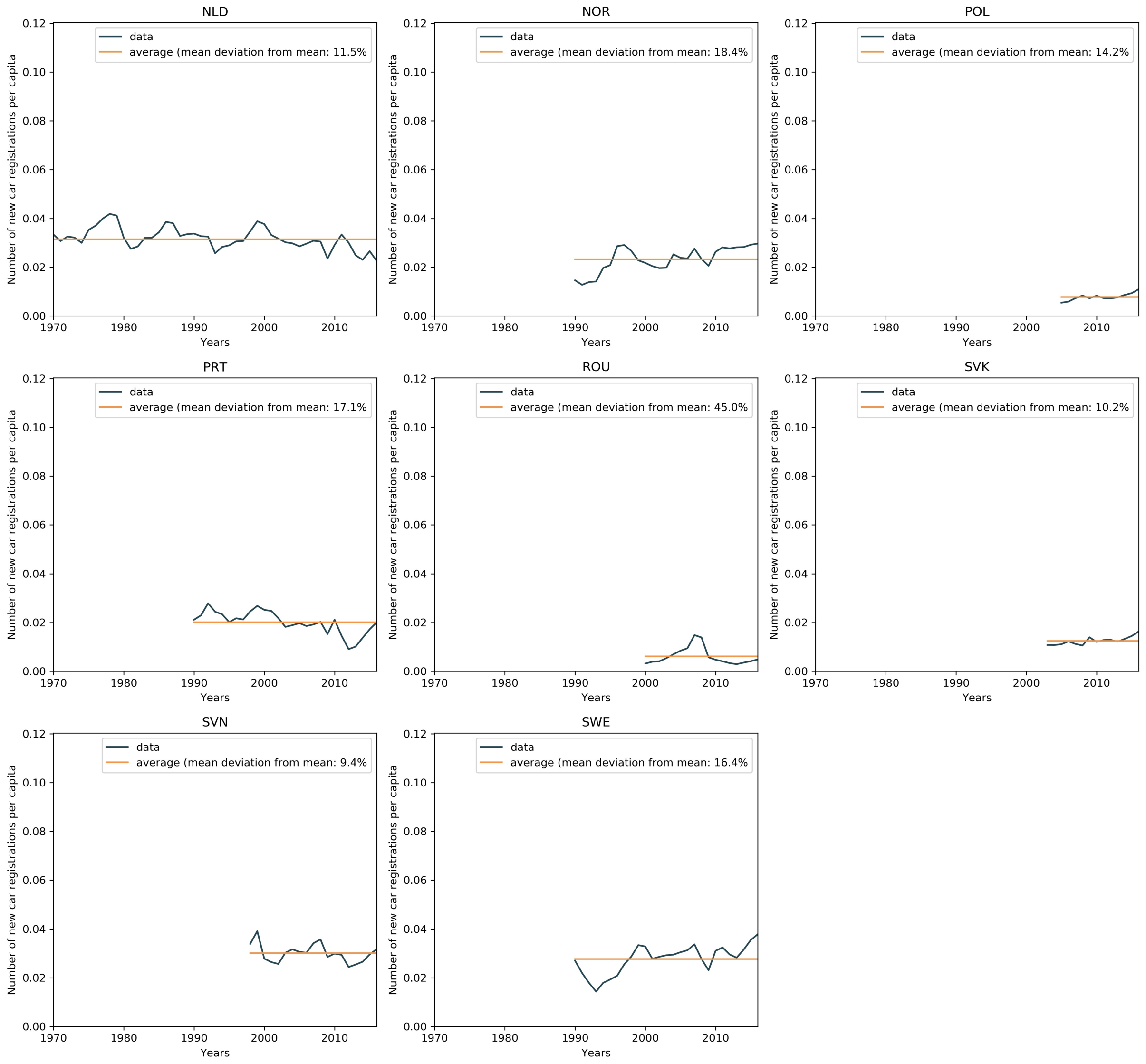

Figure S15 New car registrations per capita for all 32 countries for the data source with the highest resolution (3/3).

From the registration-per-capita-curves, we see that the registrations per capita did not change drastically over time but are rather fluctuating over a time-invariant mean (exception LUX). Therefore, our hypothesis, that we can augment missing data on new car registrations by backcasting via historic population numbers of the years before, seems legit.

Example: If a country has a full data availability from 1995 until 2016, we compute the average number of registrations per capita between 1995 and 2005. We multiply that number with historic population numbers from e.g. 1970 until 1994 to get the missing registration data during that time period.

Evidently, this only approximates reality rather coarsely, since the registrations per capita have been rising historically not only due to increasing population but also due to a higher motorisation rate. However, there are two reasons that suggest this approach is valid for our purposes:

1 From the countries where we have data back to 1970, there is no clear trend towards a rising number of registrations per capita (exception LUX). To operate within reliable boundaries, we limit our backcasting to 1970 and exclude previous years.

2 The goal of this project is to compare the age distribution of the car stock with the new car registrations in the corresponding years (e.g. 20 yr.-old cars with the new registrations in 1996). From this comparison, we can derive the cumulative survival probability of cars. The most important data (that shape the survival probability curves) is in the region of car ages between 0 and 20...30. For the countries for which we lack data back to 1970, we still have data back to 1995/6 (on average), corresponding to car ages of 20/21. Hence, the augmentation will on average be for car ages greater than 20, where less cars are still in-service in 2016 and the influence on the survival probability curves is becoming less important.

To check the validity of our approach, we do backcasting also for countries where we *have* data and compare our generated data with the actual data, see Figure S16.

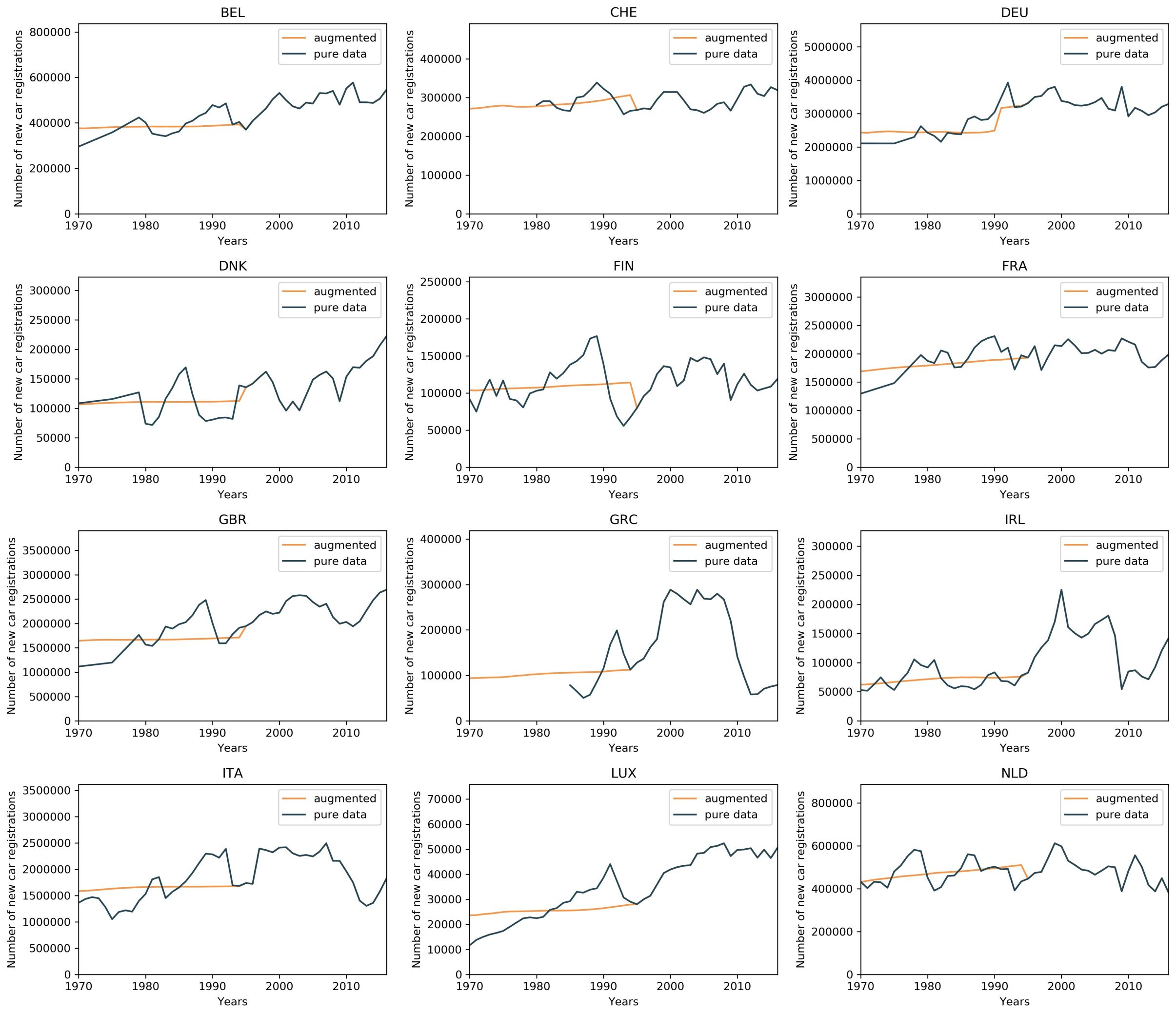

Figure S16 Validation of backcasting of new registrations: Comparison of backcasted and real data for countries with long historical time series.

The observations from the validation of our backcasting approach are:

- Economic fluctuations cannot be captured with this approach.
- For some countries, like ITA, LUX, or GBR, the backcasted data does not represent the increase in new registrations very well. This indicates that an increasing motorization rate is a second driver for the number of car registrations besides increase in population.
- However, for the majority of the observed countries (foremost DNK, FIN, DEU, IRL, NLD, and CHE) augmented data is well aligned with the real data.

We conclude that backcasting is a valid approach to augment the new car registrations time series. This is in particular justified by the diminishing importance of the "older data" for the determination of survival probabilities.

Appendix E: Imports and exports data.

Imports and exports data has been gathered from national statistics offices and other reports, see Table S5 for an overview on data availability, its resolution (whether it contains only totals, or a more detailed age resolution), and the corresponding sources. Figures S17 and S18 show the age distributions of the imports and exports of used cars for the countries where such data could be sourced from national statistics.

Table S4 Data on imported and exported cars from national statistics (1/2). If data sets are resolved by car age, the age resolution is stated. Otherwise, only totals are given.

	Imports: available years (age resolution)	Imports (ref)	Exports: available years (age resolution)	Exports (ref)
AUT	1992-2016 (totals)	[59]	-	-
BEL	1991-2016 (totals)	[60]	2006-2009 (totals)	[93]
BGR/ CHE	—	—	—	—
CYP	2003-2016 (totals)	[62]	—	—
CZE	2007-2016 (age resolution: 1, 2-3, 4-5, 6-10, 11-15, 16+)	[94]	2006-2016 (totals)	[94]
DEU	—	—	2005-2014 (totals)	[89, 93]
DNK	2004-2009 (totals)	[93]	2004-2009 (totals)	[93]
ESP	—	—	—	—
EST	- 2007, 2009, 2011-2013 (age resolution: 1-20)<br>- 2015 (age resolution: 1-100)<br>- 2006, 2008, 2010, 2014, 2016 (totals)	[95]	—	—
FIN	- 2010-2016 (totals)<br>- 2003-2009 (age resolution: 1-24, 25+)	[93, 96]		
FRA/ GBR	—	—	—	—
GRC	1985-2016 (totals)	[71]		
HRV	—	—	—	—
HUN	1999-2016 (totals)	[73]	—	—
IRL	- 2005-2016 (age resolution: 1-12, 13+)<br>- 1985-2004 (totals)	[97, 74]	—	—
ISL	—	—	—	—
ITA	2012-2016 (totals)	[98]	2012-2016 (totals)	[98]
LIE	—	—	—	—
LTU	2003-2016 (totals)	[a]	—	—
LUX	2005-2016 (totals)	[99]	2005-2016 (totals)	[99]
LVA	- 1997-2003 (age resolution: 1-19, 20+)<br>- 2004-2016 (age resolution: 1-66,67+)	[79]	—	—
MLT	- 1998-2014 (totals)<br>- 2015-2016 (age resolution: 1-2,35,6-10,11-20,21-30,41-50,51-60,61-70,71-80,81+)	[80]	—	—
NLD	- 2005-2008 (age resultion: 1-20, 21+)<br>- 2009-2012 (age resultion: 1-26, 27+)<br>- 2013-2016 (age resultion: 1-24, 25+)	[100]	2000-2016 (age resolution: 1-23, 24+)	[101]

For some countries, the age distributions of car imports are relatively stable (e.g. NOR, NLD, SWE), while for others, the average age of imported used cars has changed considerably over time (e.g. FIN, LVA). Note that the Netherlands have experienced an increased number of exports of relatively new cars (3-7 years) in the 2010s as a second peak in addition to the one at car ages around 10-15 years. More exports data would be needed to analyse whether exports of used cars might rather show a bimodal distribution than a Gaussian with one peak. This would have implications for using a Gaussian in the data-fitting approach to estimate CSPs (see Appendix B).

Table S5 Data on imported and exported cars from national statistics (2/2). If data sets are resolved by car age, the age resolution is stated. Otherwise, only totals are given.

	Imports: available years (age resolution)	Imports (ref)	Exports: available years (age resolution)	Exports (ref)
NOR	1990-1999 (totals)<br>- 2000-2016 (age resolution: 1-115)	[b]	—	—
POL	2002-2016 (age resolution: 1-4, 5-10, 11+)	[83]	—	—
PRT	—	—	—	—
ROU	2007-2016	[84]	—	—
SVK	- 2003-2004 (totals)<br>- 2005-2009 (age resolution: 1-26, 27+, "imported cars with unknown age" are distributed equally among all age bins)<br>- 2010-2016 (totals)	[c]	—	—
SVN	1998-2016 (totals)	[86]	—	—
SWE	- 1990-2008 (totals)<br>- 2009-2016 (age resolution: 1-20, 21+)	[d]	2009-2016 (age resolution: 1-20, 21+)	[102]

Footnote [a]): 2012-2016 from Statistics Lithuania [88]. For 2004-2011, the sum of new and used car registrations is taken from Statistics Lithuania [88]. For 2006-2011, the ratio between new and used cars is taken from Vanherle & Vergeer [89]. For 2004-2006, the average ratio between new and used cars from 2006-2008 from Vanherle & Vergeer [89] is applied to this time period. For 2003, the difference in new registrations data from Eurostat and ACEA is used.

Footnote [b]): 1990-1999 from difference in new registrations data from Eurostat and NatStat/ACEA. 2000-2016 from OFV (Information Council for Road Traffic) [82].

Footnote [c]): 2003-2004 from difference in new registrations data from Eurostat and NatStat/ACEA. 2005-2009 from Mehlhart et al. [93]. 2010-2016 from difference in new registrations data from Eurostat and NatStat/ACEA.

Footnote [d]): 1990-2008 from difference in new registrations data from Eurostat and NatStat/ACEA. 2009-2016 from Trafikanalys [102].

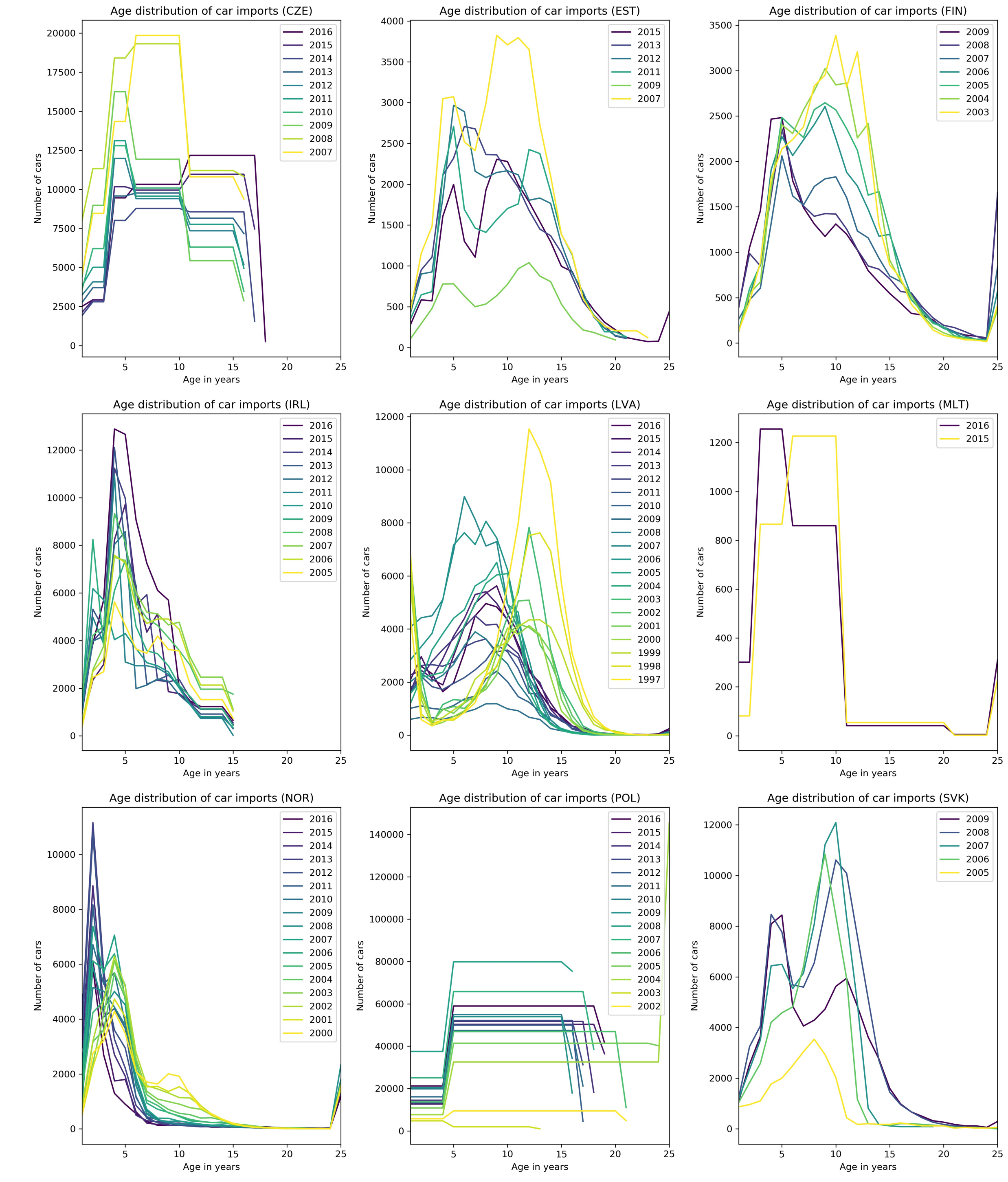

Figure S17 Age distribution of imports time series for all nine countries where such data could be sourced, see Table S5 for data sources.

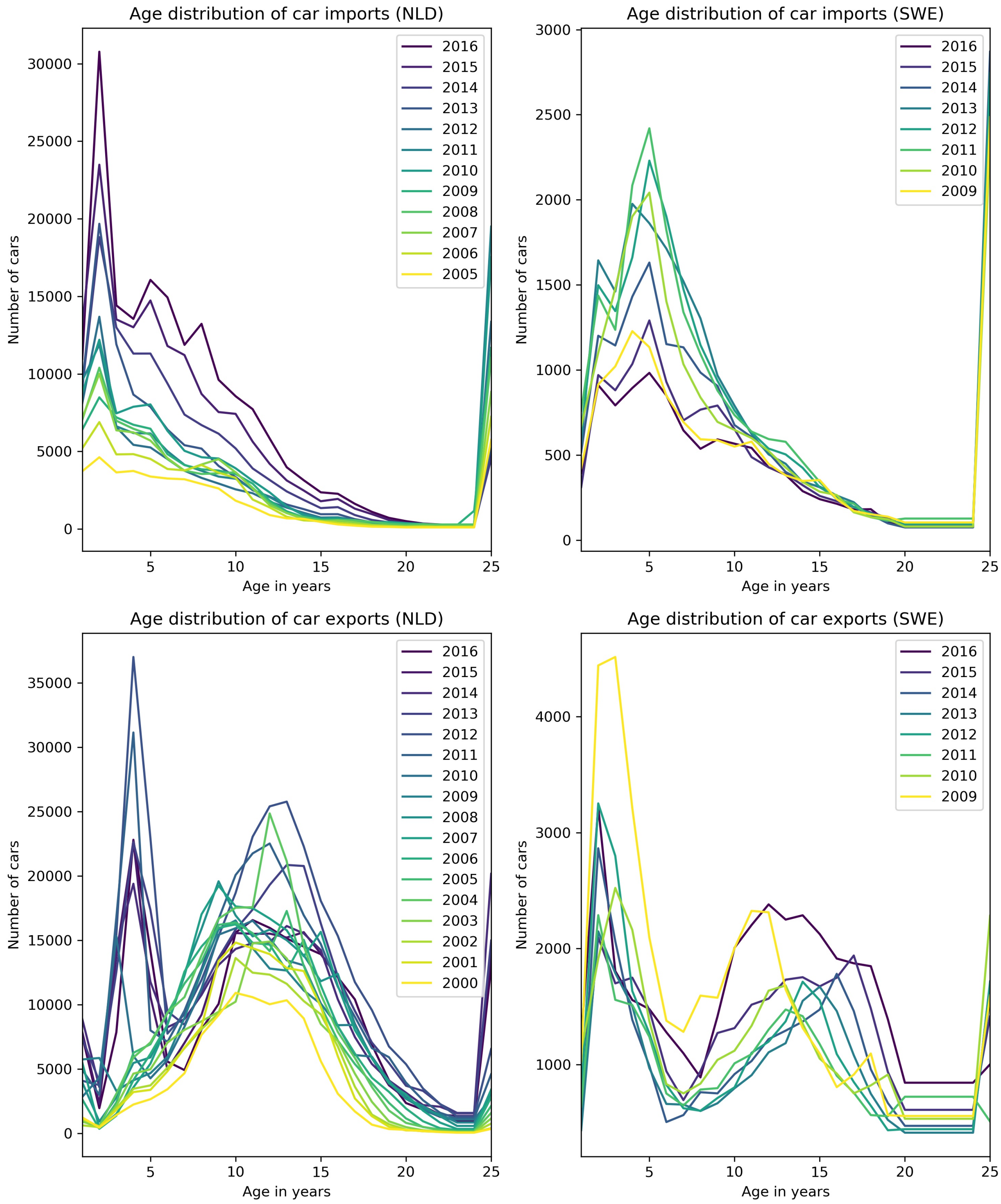

Figure S18 Age distribution of imports and exports time series for the Netherlands and Sweden for which such data could be sourced, see Table S5 for data sources.

We further augment the import totals data sets from national statistics by making use of our finding from Figures S10-S12 that some data sources for new car registrations contain imported used cars, while others do not. For some countries, some data sources definitely include imports of used cars, for others it is unknown so far whether higher registration numbers contain imports and if so, whether they contain all car ages (or e.g. only the imports of one/two yr.-old cars). In the following, we verify that the difference of new-cars-only vs. new-and-used-cars data sources amounts exactly to the total number of used car imports. The difference between these two data sources is depicted in Figure S19. Taking the difference in new registration totals between two data sources (one with new-cars-only and one with imports-included) as a proxy for the imports of used cars is a stopgap to augment the imports data that is subject to quite high uncertainty. However, the comparison in Figure S19 shows that for recent years the two different sources match quite well, and over the whole time series they show on average little differences.

In further considerations, we use the imports data from national statistics where available and fill gaps with the new-registrations-derived imports data where no national statistics are available, see Table S5. Hence, for LTU, SVK, SWE, and NOR, import totals could be augmented with the new-registrations-derived imports data, see Figure S19.

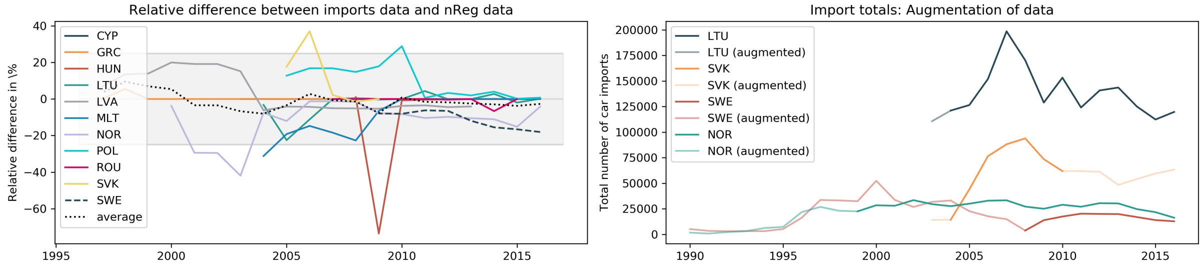

Figure S19 *Left:* Difference between total number of imports as reported by national statistics offices (and others) and the new-registrations-derived imports data, i.e. the difference in new registrations data between different data sources – mainly Eurostat vs. ACEA/NatStat, see Figures S10-S12.
Right: Augmentation of national statistics data with new-registrations-derived imports data.

Appendix F: Cumulative survival probabilities.

We compute cumulative survival probabilities (CSPs) via Eq. 1 using age-resolved stock data and historical time series of new car registrations. For the latter, we use the raw data that has been augmented by the backcasting approach (see Appendix D). Figures S20-S22 show the CSPs for all 31 countries where stock and new registration data is available.

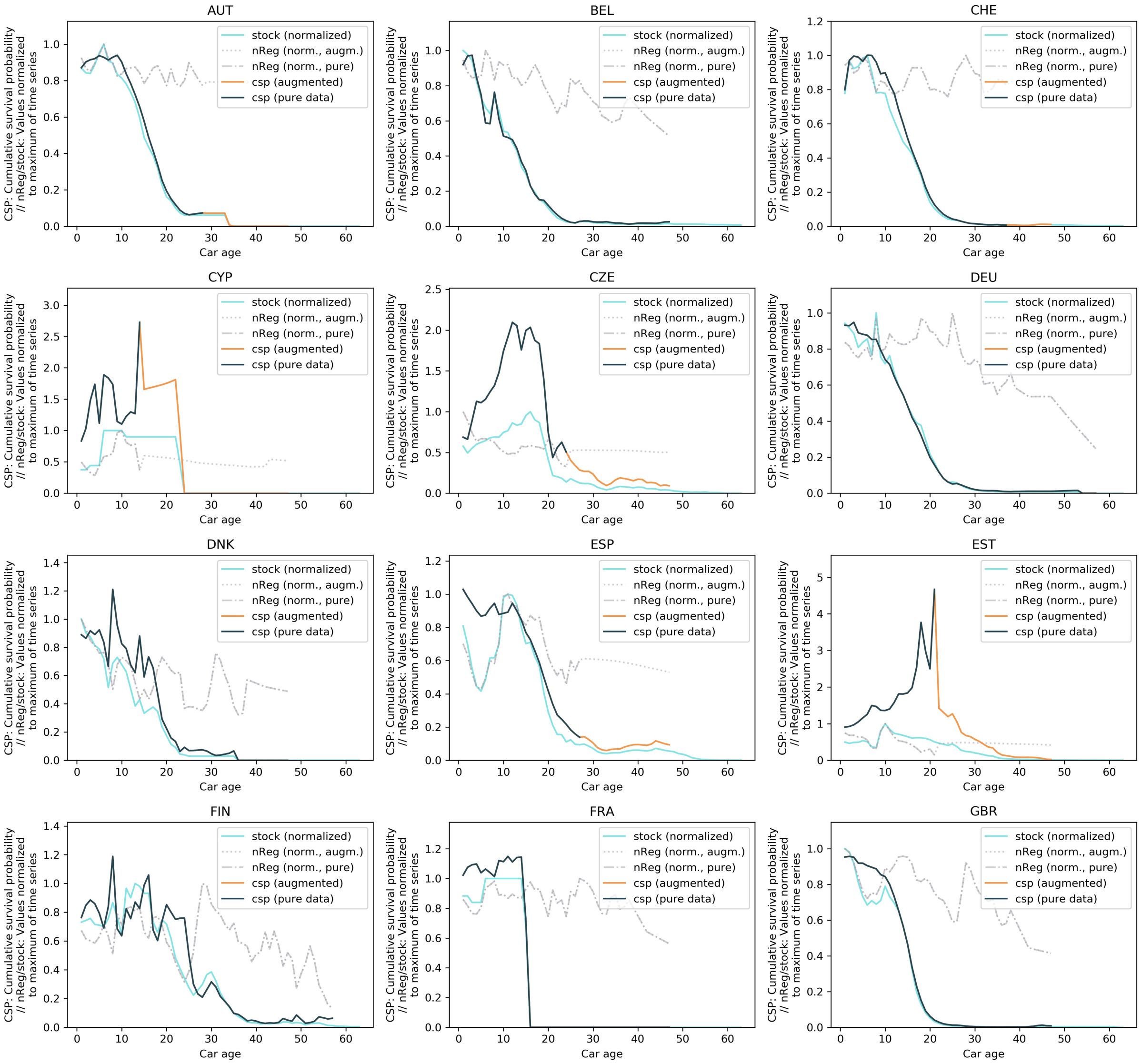

Figure S20 Cumulative survival probabilities (CSPs) for all 31 countries with available data (1/3). Underlying car stock data and data of new registrations (nReg) is shown normalized to the maximum of the time series (maximum = 1). For new registrations, both pure data and (via backcasting) augmented data is shown. Accordingly, also the CSP curves are partially relying on augmented data.

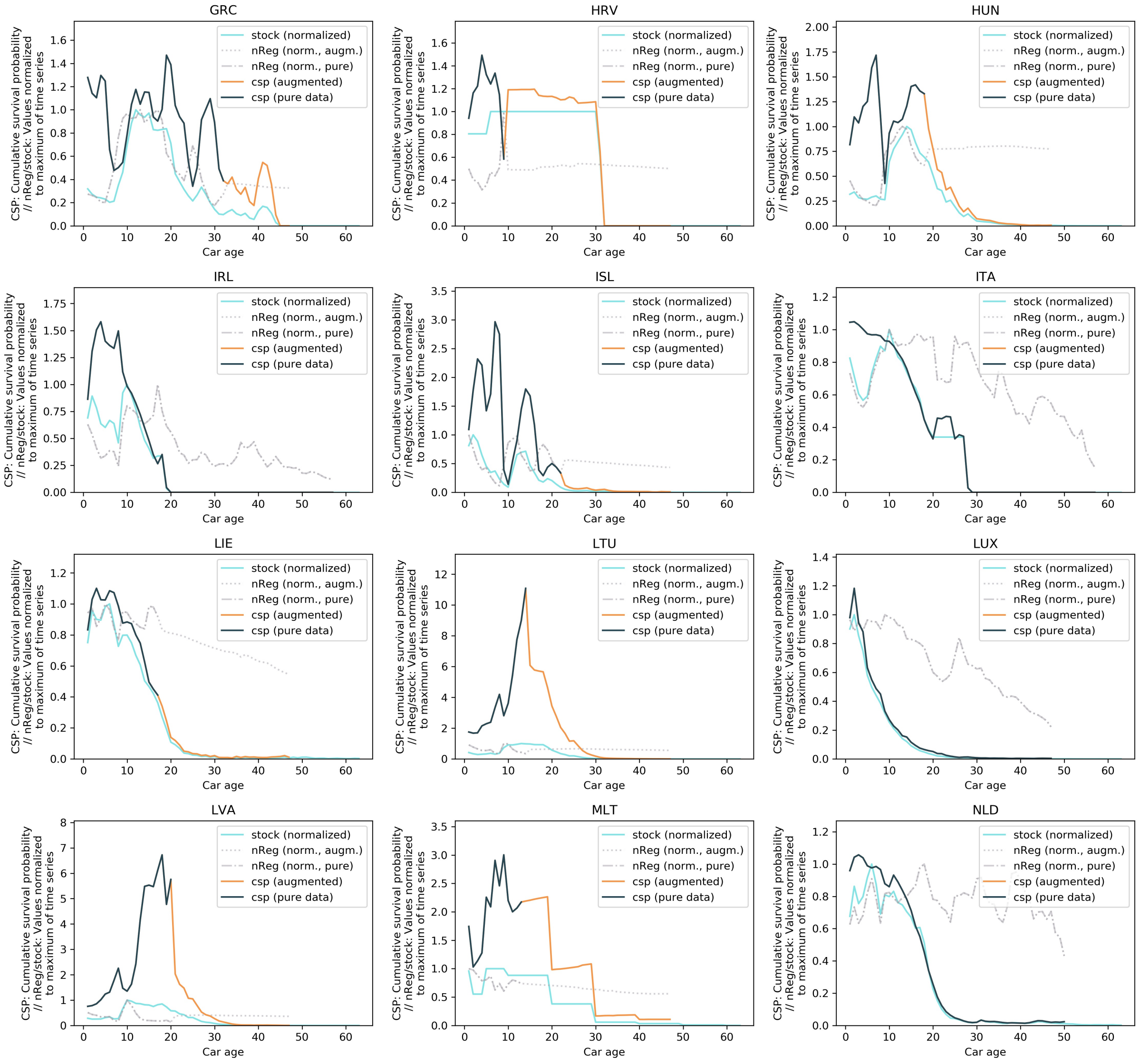

Figure S21 Cumulative survival probabilities (CSPs) for all 31 countries with available data (2/3). Underlying car stock data and data of new registrations (nReg) is shown normalized to the maximum of the time series (maximum = 1). For new registrations, both pure data and augmented data (via backcasting) is shown. Accordingly, also the CSP curves are partially relying on augmented data.

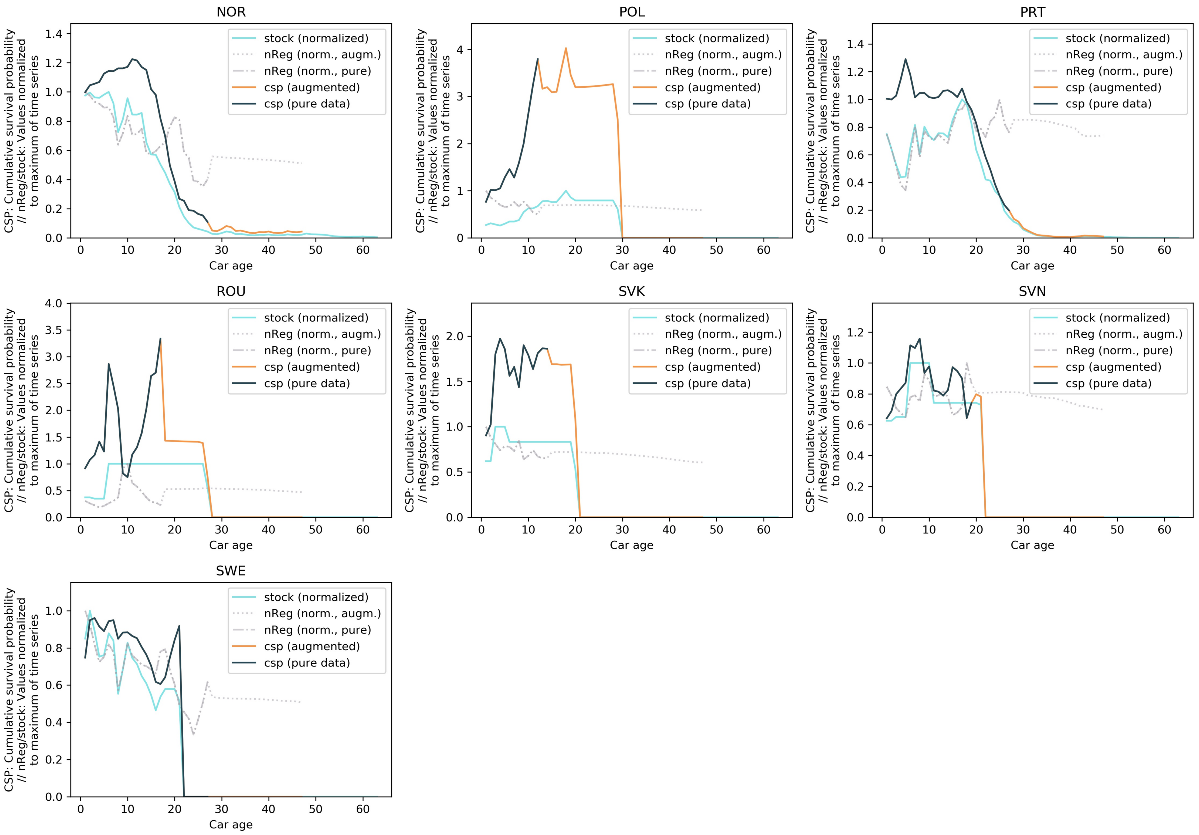

Figure S22 Cumulative survival probabilities (CSPs) for all 31 countries with available data (3/3). Underlying car stock data and data of new registrations (nReg) is shown normalized to the maximum of the time series (maximum = 1). For new registrations, both pure data and (via backcasting) augmented data is shown. Accordingly, also the CSP curves are partially relying on augmented data.

The standard fitting approach in existing literature uses a Weibull curve, e.g. see Oguchi & Fuse [54]. However, some countries do not show a continuous decline in the CSP with increasing car age. In contrast, the CSP rises from a starting value of 1 up to values of 7 for higher car ages. This is due to imports of used cars that are present in the car stock data, but are not considered in the new registrations data. Hence, e.g. more cars of age 10 are in the stock than what we would have expected from only the corresponding new registrations ten years ago.

Therefore, the sum of a Weibull and a Gaussian curve is fitted to the raw CSP data (see Appendix B for an explanation why a Gaussian curve is suited to account for the imports of used cars). The fitting procedure is sequential. First, a Weibull curve starting at a value of 1 is fitted to the raw data, maximizing the R^2. Then, a Gaussian curve is fitted "on top", defining the parameters of a Gaussian curve that is added to the fixed Weibull curve from the previous step, again maximizing the R^2.

In order to avoid over-fitting, the following constraints have been imposed onto the fitting parameters:

- Weibull average lifetime $\gamma \in$ [5,40]. This is done to enable faster fitting. The boundaries are not hit.
- Weibull shape parameter $\beta \in$ [2,6]. This parameter describes the steepness of the Weibull curve around the inflection point. While the lower limit is not hit, the upper one is. This is due to the fact that overflow bins of the stock data are pre-processed as sustained constant values with an abrupt decrease to 0 vehicles after the overflow bin is depleted (see Appendix C). Since this is an artefact, we limit the steepness to a maximum shape parameter of 6. The upper limit of 6 has been found empirically by analysing countries where high age resolution was available from national statistics, see e.g. cumulative survival probability curves for AUT, BEL, CHE, DEU, ESP, FIN, GBR, ITA, LUX, NLD, and NOR.
- Gaussian stretch $k = \delta \cdot \sqrt{2\pi}\sigma \in$ [2,10]. While the upper limit is not hit by any country, the lower boundary is. It is set to avoid over-fitting. Otherwise, very small Gaussian contributions are detected that may not stem from imports of used cars but from scattered data. This limit is found empirically from all 31 data sets.
- Gaussian mean $\mu \in$ [5,30]. Here, only the lower limit is hit by two countries. It is set in order to avoid peaks at very low car ages which are assumed to stem from data outliers or statistical errors. The limit has been tuned manually to reflect the situation of these two countries.
- Gaussian standard deviation $\sigma \in$ [5,30]. Here, only the lower limit is hit by a few countries. Low standard deviations only replicate data outliers. The lower limit is found empirically from all 31 data sets.

The resulting R^2 values of using only a Weibull curve vs. using the sum of a Weibull and a Gaussian, are illustrated in Figure S23.

Figure S24 shows the CSP fits for countries with a Gaussian curve fitted to the raw data. These are predominantly Eastern European countries with a high share of imported used cars compared to the market of new cars. They show a pronounced Gaussian. ISL, IRL, NOR, and MLT are exceptions that only show a small Gaussian contribution to the CSP fit, with low Gaussian means.

Figure S25 shows the CSP fits for countries without a Gaussian curve but a pure Weibull fit. These are predominantly Central and South European countries. Only the Eastern European countries GRC and SVN are also in this second group of purely Weibull-fitted countries. However, both have rather long average lifetimes of cars – an attribute that is characteristic for Eastern European countries.

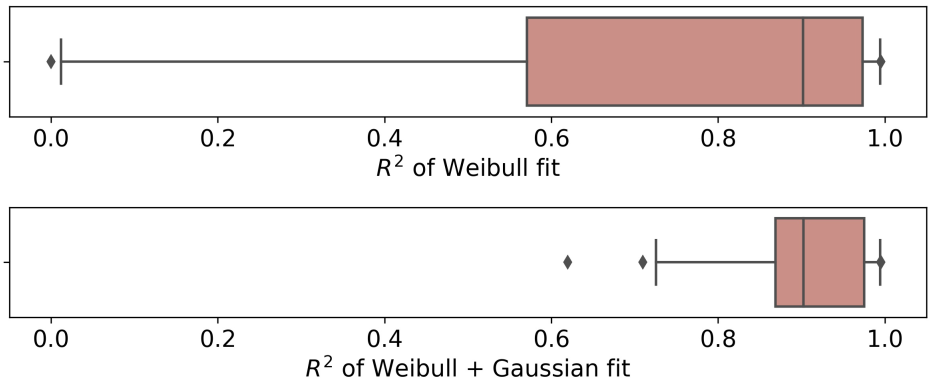

Figure S23 Boxplots of the R^2 values from pure Weibull fits and from the sum of a Weibull and a Gaussian curve. The box depicts the median and the interquartile range. The whiskers cover the 95% confidence interval.

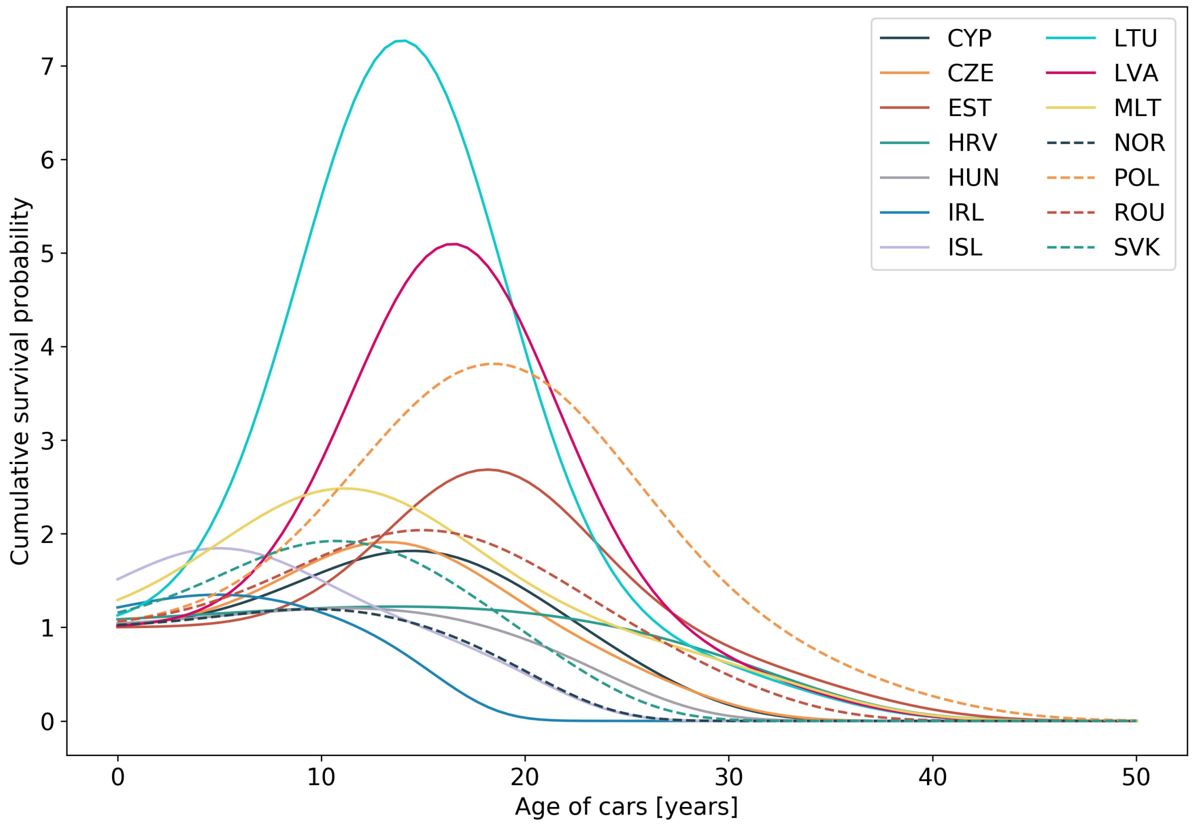

Figure S24 Cumulative survival probability (CSP) fits for all countries with a non-zero Gaussian fitted to the raw data in addition to the Weibull curve.

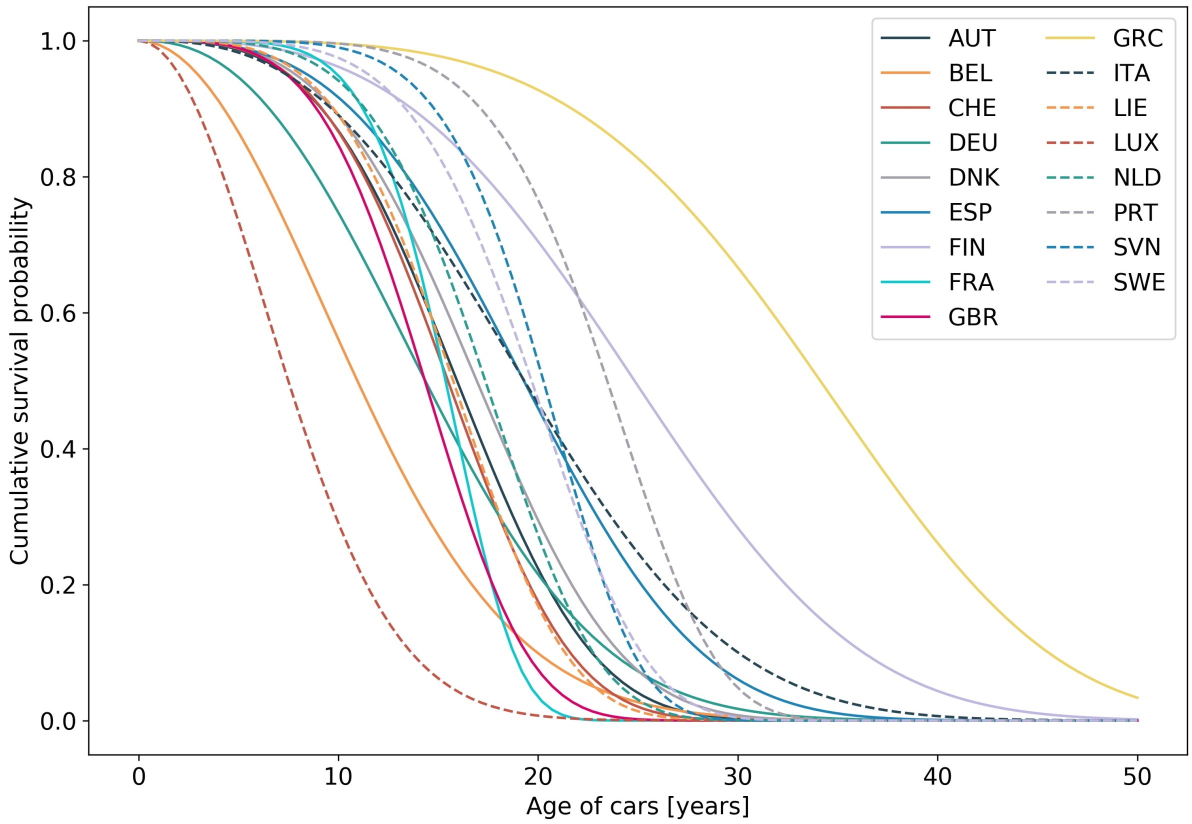

Figure S25 Cumulative survival probability (CSP) fits for all countries that feature only a Weibull curve fitted to the raw data, with no Gaussian contribution.

Finally, we compare our fitting parameters with values from Oguchi & Fuse [54], see Figure S26. Their analysis is based on data from 2008, compared to our study being based on data from 2016.

The average lifespan parameter is higher for all observed countries. The largest jump in average lifespan is observed for Italy. Interestingly, higher GDP per capita did not result in lower average lifespans as the inter-country comparison of the simplified CSP estimation method would suggest (see Figure 6 in the main manuscript). In particular due to the economic crisis of 2009, GDP alone seems not to be an accurate predictor for the average lifespan of a country's car fleet, see also Appendix I where the CSP of one country is calculated for 20 consecutive years. This is the reason why other correlations are prioritized for the simplified CSP estimation method in Figure 6. A normalization to the EU's or even the global GDP might, however, provide a more reliable result.

Appendix G: Proof of concept of new CSP estimation method.

Based on Eq. 6, the number of imported used cars is added on top of the new registrations of new cars. The exported cars are subtracted. Since historical time series of age-resolved imports and exports data are scarce, we impute missing data – mainly by extending time series back to 1990, sustaining the average age distribution of the last three years where data is available. If age-resolved imports data is available e.g. for the years 1995-2016, we assign the average values per age bin over the years 1995, 1996, and 1997 to the years 1990-1994. Since even the best data sets do not reach very far into the past, we limit this backcasting of data to the manufacturing years 1990-2016. If recent data is missing, we do analogous forecasting.

Figure S27 exemplifies the imports and exports correction for the case of the Netherlands. Imports and exports data is usually provided as the number of cars by age per calendar year. For our purposes, we transform this data from a calendar year basis to a manufacturing year basis (more precisely the year of first registrations which might differ from a vehicle's manufacturing year). Given e.g. the 2-year old cars in the stock of 2016, we require the number of cars that have been registered in 2014. This number is composed of the new registrations of new cars in 2014 and the (0-)1-yr. old cars imported in 2014, the (1-)2-yr. old cars imported in 2015, and the (2-)3-yr. old cars imported in 2016. These three numbers are summed up to base the data on the year of first registration. We do the same for exports. The result is exemplarily depicted for the Netherlands in the first panel of Figure S27. The second panel shows the correction of the new registration data by imports and/or exports data. The third panel of Figure S27 shows the resulting CSP curves for each correction step. Correcting for imports subtracts a Gaussian, while correcting for exports adds a Gaussian. Note that both imports and exports numbers are not very pronounced for the case of the Netherlands. Therefore, the deviation from the uncorrected CSP (baseline) is not too high.

Figure S28 shows the imports and exports correction of the countries that have not been shown in the main manuscript. The concept of the imports-/exports-Gaussian can also be validated here. Even the pronounced Gaussian contribution for Latvia (LVA) can be fully corrected such that only a Weibull curve remains. However, data scarcity and low data resolution often impede a fine analysis of the actual CSPs (see notes in Figure S28). Note that Sweden only has a very low number of both imports and exports. Therefore, all CSP curves look very similar.

There are a few caveats regarding the application of this new CSP estimation method. The following limitations shall be addressed in future studies:

- *Low data availability & resolution for imports and exports data (see Table S5):* Long time series of used car imports and exports with a high age resolution would be beneficial for an accurate estimation of the real CSP of a country.
- *Unharmonized data sources:* Data sets are potentially subject to errors and statistical inconsistencies. Combining data from different sources can add additional noise or cancel out existing noise in stock

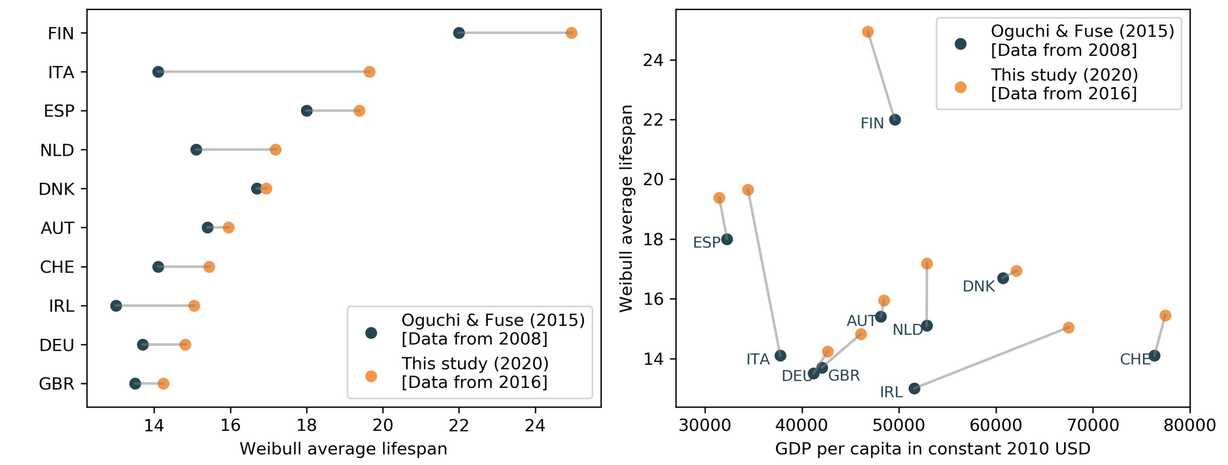

Figure S26 *Left:* Comparison of Weibull average lifespans of Oguchi & Fuse [54] and this study (see Table 1). *Right:* Weibull average lifespans vs. GDP per capita.

and new registration data. Therefore, correcting for imports and/or exports might have advantageous or adverse effects. There is a high demand for comprehensive, harmonized data sets of all required inputs for a CSP curve.

- *Gauss-curve only an approximation (see Appendix B)*: As already indicated, further analyses should validate whether a Gaussian curve is able to fit the effect of imports on the CSP of other countries as well as it does for the countries in this study. This is even more important for the exports correction, for which fewer data was available at the time of writing.

Often, the exact reason for data outliers cannot be named. This can e.g. be seen for the case of LVA, where the goodness of fit decreases with the imports correction, which can be due to noise (statistical errors) in all combined data sets, due to different data sources of stock, registration, and imports data, due to the fact that the exports correction is missing, or due to other reasons.

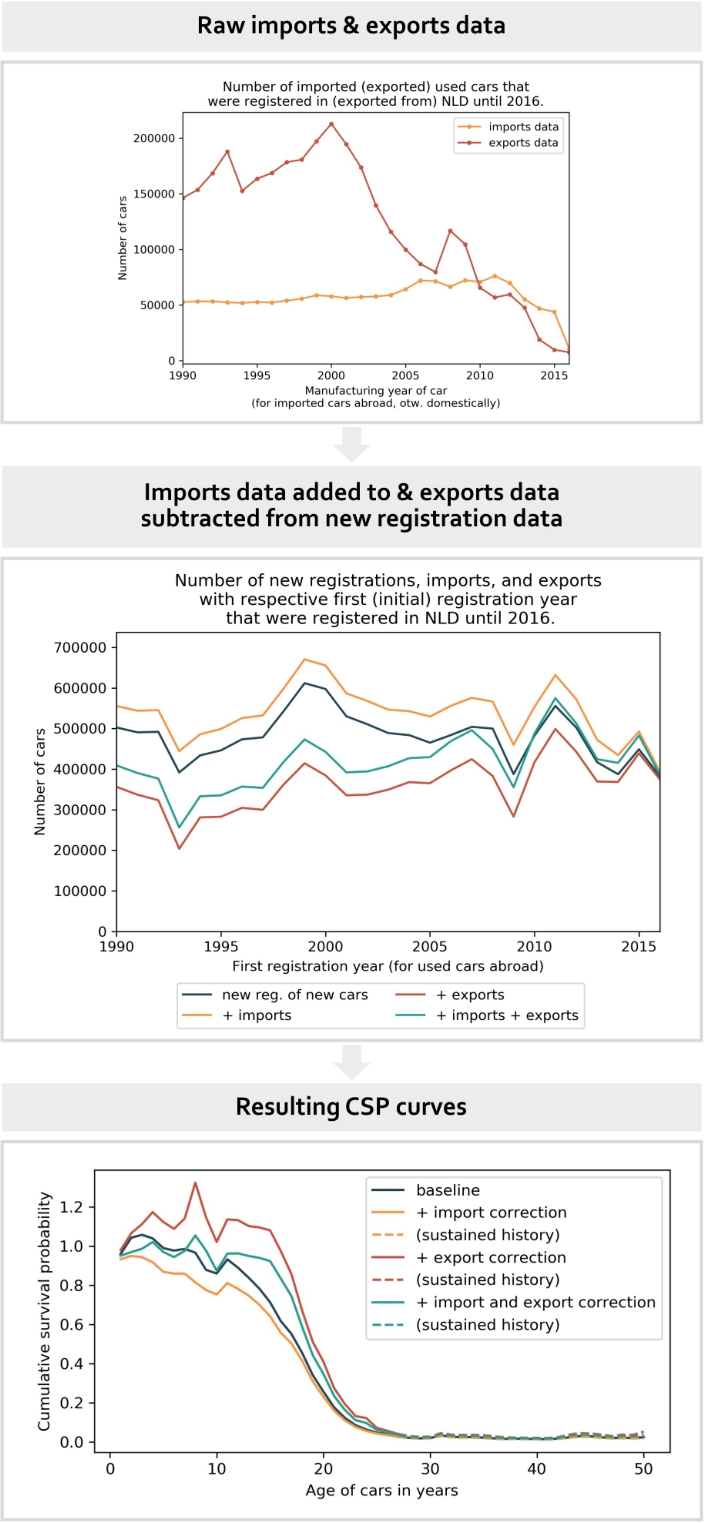

Figure S27 Imports and exports correction of raw CSP data: proof of concept for new CSP estimation method, exemplified for the case of the Netherlands (NLD). Curves that are based on augmented imports/exports data are marked as "sustained history".

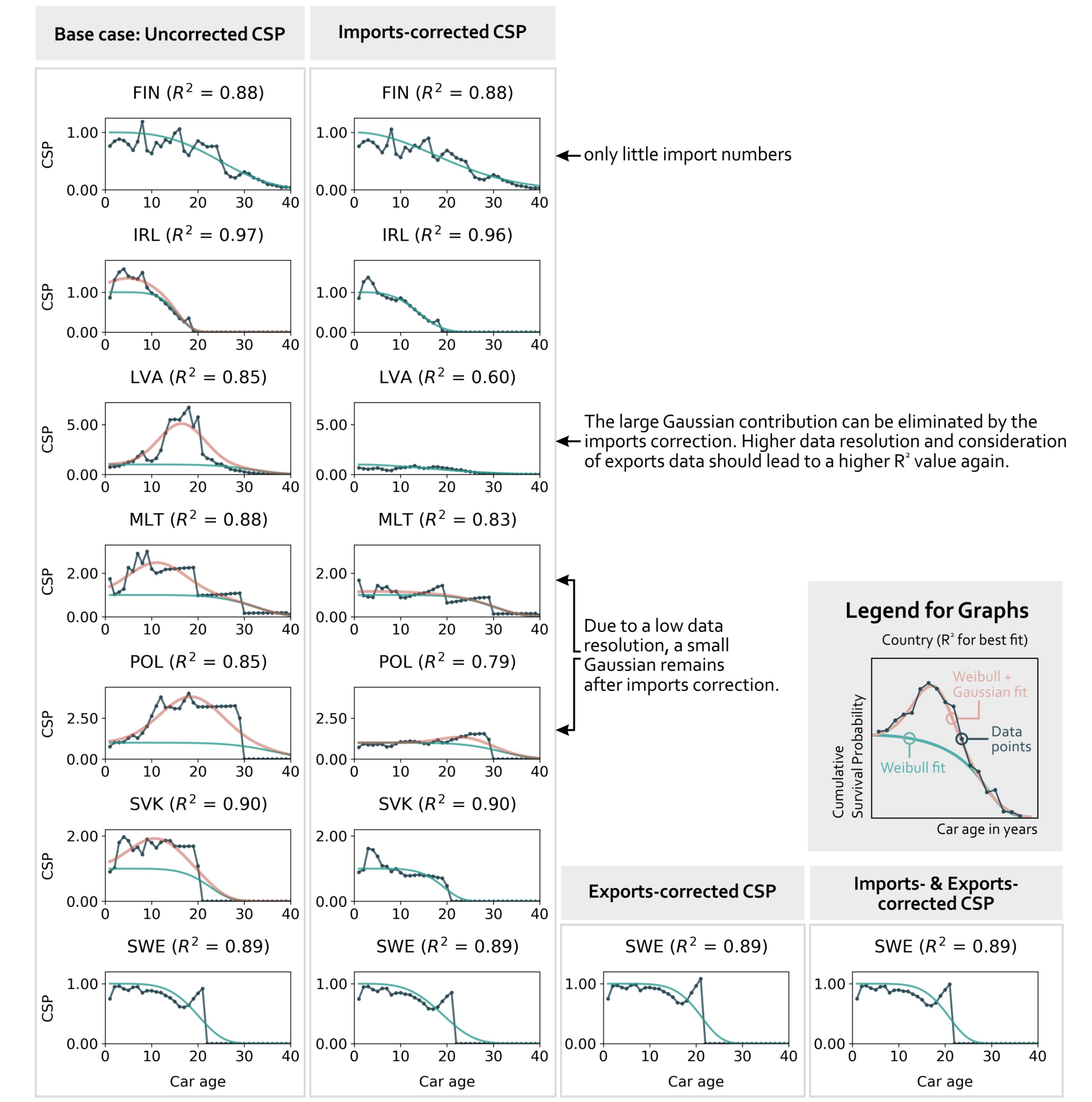

Figure S28 Proof of concept for new CSP estimation method. The uncorrected CSP curve (left column) is corrected by imported (second column) and exported cars (third column), and by both (right column) using Eq. 6. Selection of six from nine countries with available age-resolved imports data, and one from two countries with both age-resolved imports and exports data, to show the proof of concept. The analysis for the other countries can be found in the main manuscript.

Appendix H: The simplified CSP estimation method.

This subsection is divided into two parts: The first part is verifying the simplified CSP estimation method of Oguchi & Fuse [54]. In the second part, a new simplified CSP estimation method is discussed to also cover countries with high imports.

Applying the simplified estimation method of Oguchi & Fuse [54] to all 31 observed countries of this study reveals that it only works well for the countries w./o. a considerable number of imports (mainly Western European countries). Taking the country sets of Western and Eastern European countries as defined in Figure 4, we see that the median average lifespan over all Western European countries calculated using the simplified estimation method of Oguchi & Fuse [54] (with only the *total* stock number as an input) is as close as 0.5 years to the value calculated using *age-resolved* stock data, see Table 1. The median deviation for Eastern European countries is 11.2 years.

The new proposed simplified CSP estimation method is based on correlations among fitting parameters and between fitting parameters and other aggregate indicators. While the correlations for γ, δ, and μ have already been illustrated in the main manuscript, an analysis of the impact of setting β and σ to constant values follows here.

Weibull shape parameter β.

Based on an analysis of 17 countries, Oguchi & Fuse [54] suggest that the Weibull shape parameter can be set to a constant value of 3.6 without major impacts on the average lifespan and the R^2 of the CSP fits. We choose the 20 countries with highest stock data resolution to verify this finding. For AUT, BEL, CZE, DNK, EST, FIN, DEU, GRC, HUN, LVA, LTU, LUX, NLD, PRT, ESP, GBR, ISL, LIE, NOR, and CHE, we have stock data resolved by age for at least the first 25 age bins and for most cases for 1-100 year-old cars. The average Weibull shape parameter for this high-resolution data set is 3.5 (std dev: 1.1, min: 2.0, max: 6.0), very close to the value of Oguchi & Fuse [54].

We validate the sensitivity of a variation of the Weibull shape parameter β in two ways: first, parametrically by varying β from 2.0 to 6.0; second, by using a constant β of 3.5 versus the optimal parameters for each country (see Table 1). We measure the impact of these two sensitivity analyses on the Weibull average lifespan γ and the R^2 of the Weibull fit for the full data set of 31 countries.

1 *Parametric variation:* γ is 1.3 years higher (std dev: 1.9) for $\beta = 2.0$ compared to $\beta = 3.5$, and 0.8 years lower (std dev: 1.5) for $\beta = 6.0$ compared to $\beta = 3.5$. The effects on the R^2 of the Weibull fit are below 0.05 in both directions ($\beta = 2.0$ and $\beta = 6.0$).

2 *Comparison with the optimal β:* Compared to the optimal β values for each country, setting β to 3.5 yields Weibull average lifespans that are on average 0.8 years higher (std dev: 1.5), and R^2 values that are on average 0.02 lower (std dev: 0.03).

All variables observed are only slightly affected by a change in the Weibull shape parameter. Note also that outlier β's are mostly caused by low stock data resolution. Hence, we can confirm the finding from Oguchi & Fuse [54] that β can be set constant – in our case to a value of 3.5.

Gaussian standard deviation σ.

The mean Gaussian standard deviation σ of the set of 7 high-resolution countries is 5.2 (std dev: 0.6).[3] Taking all 31 countries into account (except for HRV, which is an outlier due to a low stock data resolution) yields an average σ value of 5.6 (std dev: 0.7). Therefore, we suggest that the Gaussian standard deviation can be approximated with a most likely range of 5.0-6.0. As for beta, we proceed with the value from the countries with highest data availability, i.e. 5.2. Due to the low standard deviations, no sensitivity analysis is needed.

[3]Note that from the 20 high-resolution countries, only 7 have a non-zero Gaussian contribution.

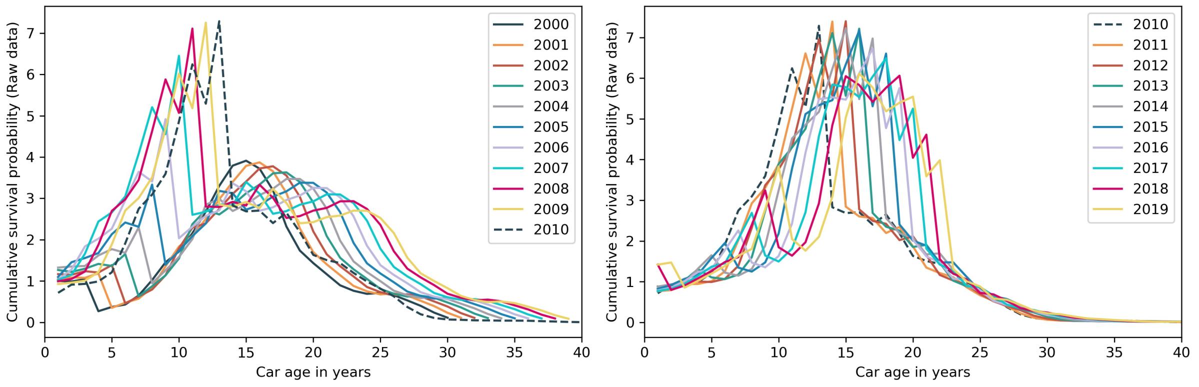

Figure S29 Cumulative survival probabilities (raw data) for Latvia based on stock data from 2000-2019.

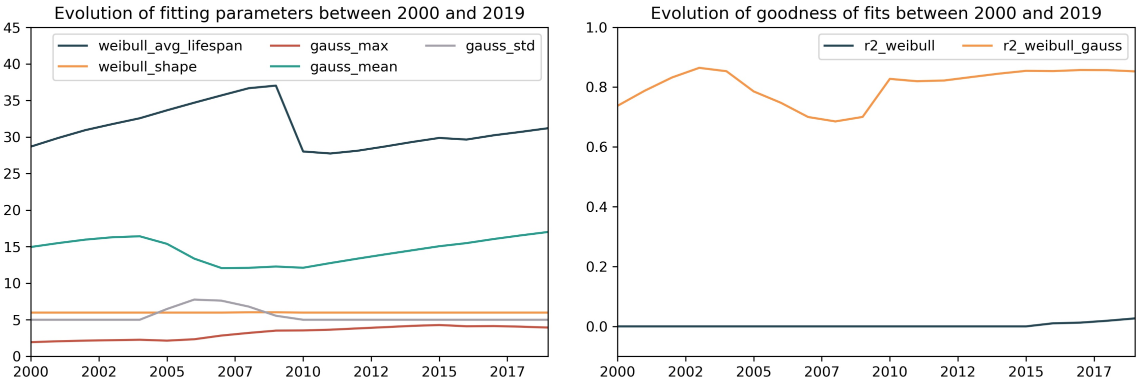

Figure S30 Fitting parameters to the raw CSP data for Latvia based on stock data from 2000-2019.

Appendix I: Time variability of CSP fitting parameters: Case study for Latvia.

CSP curves change over time. To exemplify the need for further research on *how* CSP curves evolve over time, we analyse the CSPs (Figure S29) and corresponding Weibull + Gauss fitting parameters (Figure S30) for Latvia for 20 consecutive years (2000-2019). Note that the availability of age-resolved stock data for such a long period of time is very rare.

We see that the Weibull average lifespan shows a spread of almost 10 years in the given time period. The drop in the average lifespan from 2009 to 2010 is highlighted with a dashed line in both panels of Figure S29. During the financial crisis of 2009, a lot of old cars in the fleet have been scrapped or deregistered – maybe to avoid operational costs, incl. insurance, etc., which might be an important consideration for corporate car fleets. This causes a drop in the average lifespan, even if there was no scrappage premium in Latvia at that time. The Gaussian mean is also affected considerably – which can be seen from the shifting peak of the CSP data in Figure S29.

Appendix J: The influence of data resolution.

A high resolution of the stock data set – at best continuous age resolution – is crucial for the accurate determination of CSPs and the corresponding fits to the CSP data.[4] We showcase this for four selected countries (AUT, CZE, DEU, and ESP) in Figure S31. For all of them, the stock data from national statistics (NatStat) presents the highest resolution. Accordingly, also the R^2 values are highest for this stock data source. For these select countries, CSP curves are also computed from data sources with a lower resolution, i.e. ACEA and UNECE values. This causes a deviation from the most accurate Weibull average lifetime (based on NatStat data) of up to four years. The case of CZE shows that also the Gaussian mean can deviate by more than four years if a lower resolution of the stock data is underlying for the computation of the CSP data. This exemplifies the high value of this paper in gathering high-resolution data from various national statistics offices.

For the countries where we have a full age resolution of the stock data from national statistics, we compare the fitting parameters of the CSP fits with those based on stock data from ACEA and UNECE, see Figure S32. Overall, high stock data resolution yields higher R^2 values, and the CSP fitting parameters are concentrated in the mid range, only rarely going to the limits (like for ACEA and UNECE based CSP fits).

[4] Equal importance has the length of the historical time series of new registrations. Here, however, data availability is higher. Additionally, the backcasting approach described in Appendix D allows to impute missing data with reasonable accuracy.

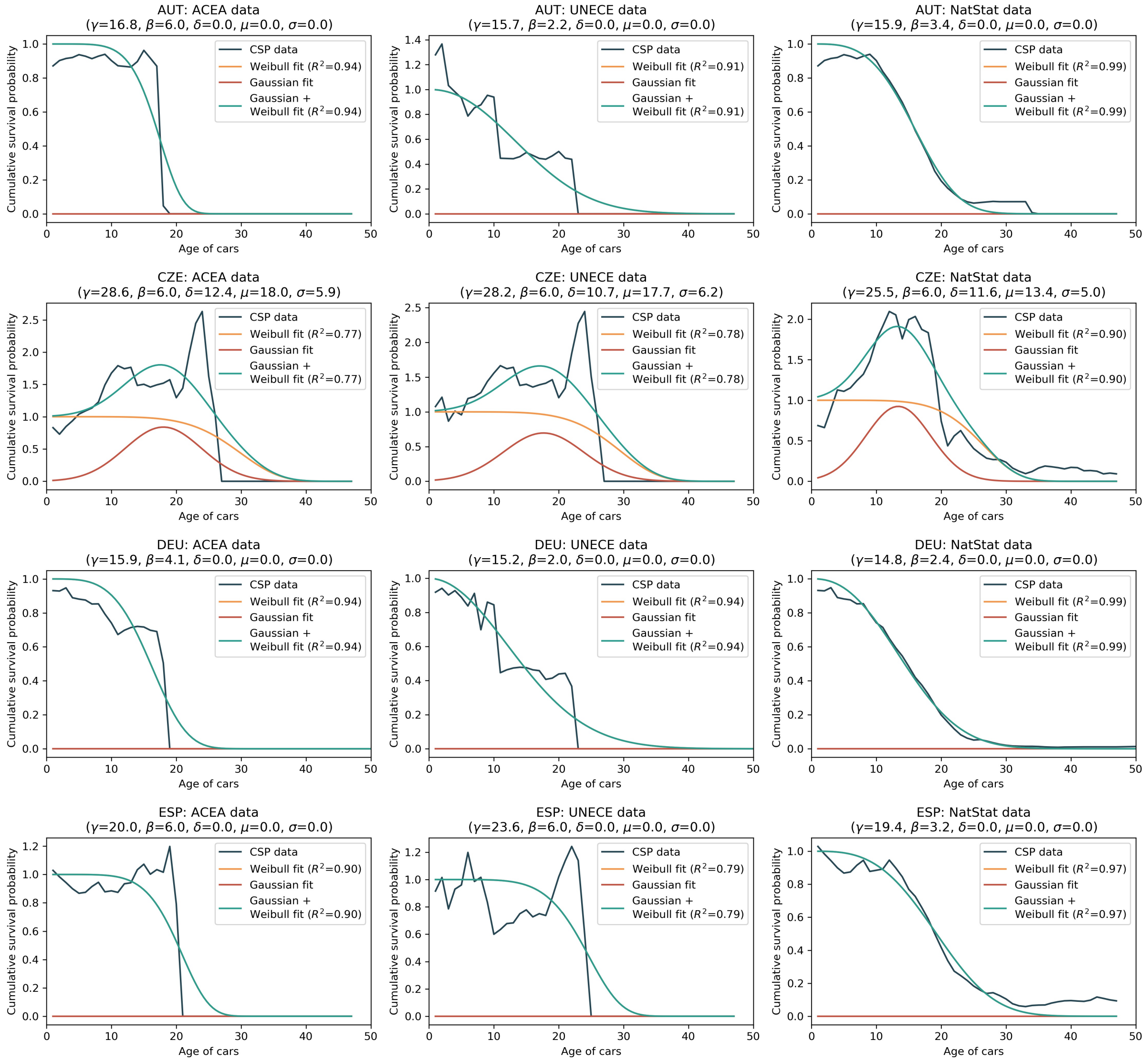

Figure S31 Fits to cumulative survival probability (CSP) data for selected countries and different stock data sources: ACEA [24], UNECE [25], and national statistics (NatStat, sources see Table S1). Fitting parameters are the Weibull average lifetime, γ, the Weibull shape parameter, β, the Gaussian maximum, δ, the Gaussian mean, μ, and the Gaussian standard deviation, σ.

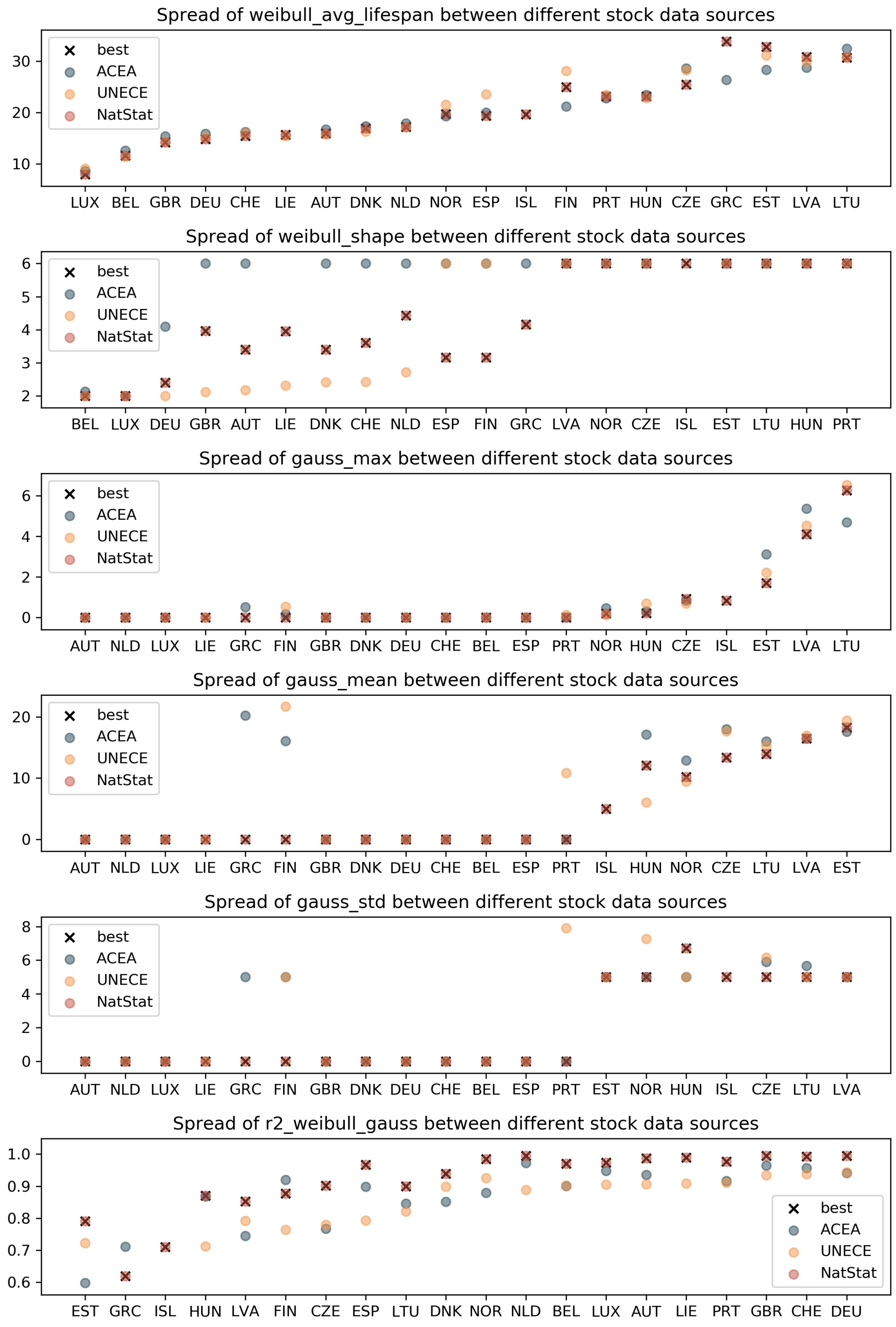

Figure S32 Fitting parameters for cumulative survival probability (CSP) data for countries with a high stock data resolution from national statistics (NatStat) – see Table S1 – in comparison to fitting parameters for CSP data based on stock data from ACEA [24] and UNECE [25]. The best data source (highest data resolution) is marked with an 'x'. Fitting parameters are the Weibull average lifetime, γ, the Weibull shape parameter, β, the Gaussian maximum, δ, the Gaussian mean, μ, and the Gaussian standard deviation, σ.

Appendix K: Attached Excel sheets.

The accompanying Excel file contains the following data for 31 European countries:

- Final survival rates, computed using Eq. 6.
- Age-resolved stock data.
- New registration data.
- Data on total imports and exports of used cars.
- Age-resolved data on imports and exports of used cars.

References

1. Van Wee, B., De Jong, G., Nijland, H.: Accelerating Car Scrappage: A Review of Research into the Environmental Impacts. Transport Reviews **31**(5), 549–569 (2011). doi:10.1080/01441647.2011.564331
2. Bolla, M., Held, M., Küng, L., Georges, G., Boulouchos, K.: Vehicle motion patterns for energy research: Comparison of annual mileage using vehicle and person-based data. Swiss Transport Research Conference **May 16-18** (2018). doi:10.3929/ethz-b-000315035
3. Laborda, J., Moral, M.J.: Scrappage by age: Cash for Clunkers matters! Transportation Research Part A: Policy and Practice **124**, 488–504 (2019). doi:10.1016/j.tra.2019.04.014
4. Fridstrøm, L., Østli, V., Johansen, K.W.: A stock-flow cohort model of the national car fleet. European Transport Research Review **8**(3), 22 (2016). doi:10.1007/s12544-016-0210-z
5. Spitzley, D.V., Grande, D.E., Keoleian, G.A., Kim, H.C.: Life cycle optimization of ownership costs and emissions reduction in US vehicle retirement decisions. Transportation Research Part D: Transport and Environment **10**(2), 161–175 (2005). doi:10.1016/j.trd.2004.12.003
6. Van Wee, B., Moll, H.C., Dirks, J.: Environmental impact of scrapping old cars. Transportation Research Part D: Transport and Environment **5**(2), 137–143 (2000). doi:10.1016/S1361-9209(99)00030-9
7. Zorpas, A.A., Inglezakis, V.J.: Automotive industry challenges in meeting EU 2015 environmental standard. Technology in Society **34**(1), 55–83 (2012). doi:10.1016/j.techsoc.2011.12.006
8. Karagoz, S., Aydin, N., Simic, V.: End-of-life vehicle management: a comprehensive review. Journal of Material Cycles and Waste Management **22**(2), 416–442 (2020). doi:10.1007/s10163-019-00945-y
9. Mian, A., Sufi, A.: The Effects of Fiscal Stimulus: Evidence from the 2009 Cash for Clunkers Program. The Quarterly Journal of Economics **127**(3), 1107–1142 (2012). doi:10.1093/qje/qjs024
10. Aldred, R., Tepe, D.: Framing scrappage in Germany and the UK: from climate discourse to recession talk? Journal of Transport Geography **19**(6), 1563–1569 (2011). doi:10.1016/j.jtrangeo.2011.04.008
11. Kaul, A., Pfeifer, G., Witte, S.: The incidence of Cash for Clunkers: Evidence from the 2009 car scrappage scheme in Germany. International Tax and Public Finance **23**(6), 1093–1125 (2016). doi:10.1007/s10797-016-9396-1
12. Maleček, P., Melcher, O.: Cross-Border Effects of Car Scrapping Schemes: The Case of the German Car Scrapping Programme and its Effects on the Czech Economy. Prague Economic Papers **25**(5), 560–576 (2016). doi:10.18267/j.pep.567
13. Li, S., Linn, J., Spiller, E.: Evaluating "Cash-for-Clunkers": Program effects on auto sales and the environment. Journal of Environmental Economics and Management **65**(2), 175–193 (2013). doi:10.1016/j.jeem.2012.07.004
14. Kagawa, S., Nansai, K., Kondo, Y., Hubacek, K., Suh, S., Minx, J., Kudoh, Y., Tasaki, T., Nakamura, S.: Role of Motor Vehicle Lifetime Extension in Climate Change Policy. Environmental Science & Technology **45**(4), 1184–1191 (2011). doi:10.1021/es1034552
15. Kagawa, S., Hubacek, K., Nansai, K., Kataoka, M., Managi, S., Suh, S., Kudoh, Y.: Better cars or older cars?: Assessing CO2 emission reduction potential of passenger vehicle replacement programs. Global Environmental Change **23**(6), 1807–1818 (2013). doi:10.1016/j.gloenvcha.2013.07.023
16. Luoma, J., Sivak, M.: Interactions of environmental and safety measures for sustainable road transportation. European Transport Research Review **4**(4), 189–199 (2012). doi:10.1007/s12544-012-0078-5
17. Hahn, R.W.: An Economic Analysis of Scrappage. The RAND Journal of Economics **26**(2), 222 (1995). doi:10.2307/2555914
18. Marin, G., Zoboli, R.: Effectiveness of car scrappage schemes: Counterfactual-based evidence on the Italian experience. Economics of Transportation **21**, 100150 (2020). doi:10.1016/j.ecotra.2019.100150
19. Brand, C., Anable, J., Tran, M.: Accelerating the transformation to a low carbon passenger transport system: The role of car purchase taxes, feebates, road taxes and scrappage incentives in the UK. Transportation Research Part A: Policy and Practice **49**, 132–148 (2013). doi:10.1016/j.tra.2013.01.010
20. Poliscanova, J.: What coronavirus means for the EU car industry and what we can learn from the 2009 crisis (Transport & Environment) (2020). `https://www.transportenvironment.org/newsroom/blog/what-coronavirus-means-eu-car-industry-and-what-we-can-learn-2009-crisis` Accessed 2020-04-19
21. Lachapelle, U.: Using an accelerated vehicle retirement program (AVRP) to support a mode shift: Car purchase and modal intentions following program participation. Journal of Transport and Land Use **8**(2), 107–123 (2015). doi:10.5198/jtlu.2015.659
22. IPCC: Summary for Policymakers: Global Warming of 1.5°C. An IPCC Special Report on the Impacts of Global Warming of 1.5°C Above Pre-industrial Levels and Related Global Greenhouse Gas Emission Pathways., (2018)
23. United Nations Framework Convention on Climate Change: Paris Agreement. Technical report, Unite Nations Framework Convention on Climate Change, Paris (2015). `https://unfccc.int/sites/default/files/english_paris_agreement.pdf`
24. ACEA (European Automobile Manufacturers Association): ACEA Report: Vehicles in use: Europe 2018. Technical report, European Automobile Manufacturers Association, Brussels (2018)
25. UNECE: Road Vehicle Fleet at 31 December 2016 by Age Group, Type of Vehicle, Country and Year [Statistical Database of the United Nations Economic Commission for Europe > Transport > Road Vehicle Fleet] (2016). `https://w3.unece.org/PXWeb2015/pxweb/en/STAT/STAT__40-TRTRANS__03-TRRoadFleet/02_en_TRRoadtypVeh_r.px/` Accessed 2020-02-13
26. Eurostat: Passenger cars, by age: Road transport > Road transport equipment - stock of vehicles (road_eqs) > Passenger cars, by age (road_eqs_carage) (2019). `https://ec.europa.eu/eurostat/en/web/products-datasets/-/ROAD_EQS_CARAGE` Accessed 2020-02-20
27. Statistics Austria: Stock of motor vehicles and trailers 2016 [Statistics > Transport > Road] (2016). `http://www.statistik.at/web_de/statistiken/energie_umwelt_innovation_mobilitaet/verkehr/strasse/kraftfahrzeuge_-_bestand/index.html` Accessed 2020-02-13

28. Fédération Belge et Luxembourgeoise de l'automobile et du cycle: Parc des voitures par année de première immatriculation (au 31/12/2018) [Fleet per year of first registration (at 2018-12-31)] (2018). https://www.febiac.be/public/statistics.aspx?FID=23&lang=NL Accessed 2020-02-13
29. Swiss Federal Roads Office (FEDRO): Vehicle and Vehicle Owner Registry 2016 - Data as of 2016-12-31 [Fahrzeug- und Fahrzeughalterregister (MOFIS-Register)] (2016). https://www.astra.admin.ch/astra/en/home.html Accessed 2020-02-14
30. Automotive Industry Association of the Czech Republic (Autosap.cz): Private correspondence with Mr. Pavel Ešner: Age distribution of passenger cars in use in the Czech Republic as of 2014-12-31 (2019)
31. Kraftfahrt-Bundesamt: Bestand an Kraftfahrzeugen und Kraftfahrzeuganhängern in Deutschland nach Fahrzeugalter, 1. Januar 2017 (FZ 15) [Statistik > Fahrzeuge > Bestand > Fahrzeugalter] (2019). https://www.kba.de/DE/Statistik/Fahrzeuge/Bestand/Fahrzeugalter/fahrzeugalter_node.html Accessed 2020-02-15
32. Statistics Denmark: Age distribution of passenger cars in use in Denmark as of 2016-12-31 [StatBank Denmark > Business Sectors > Transport > Transport equipment > Table BIL8] (2020). https://www.statbank.dk/
33. Spanish Dirección General de Tráfico: Parque de vehículos - Anuario 2016 [Seguridad vial > Estadísticas e indicadores > Parque de vehículos > Tablas estadísticas] (2016). http://www.dgt.es/es/seguridad-vial/estadisticas-e-indicadores/parque-vehiculos/tablas-estadisticas/ Accessed 2020-02-14
34. Road Administration of the Republic of Estonia: Passenger cars registered in the Traffic Register as of 2016-12-31 [Sõidukitega tehtud toimingute statistika > Table Arvel olevad sõiduautod seisuga 31.12.2016 (mootori maht ja võimsus)] (2016). https://www.mnt.ee/et/ametist/statistika/soidukid/soidukitega-tehtud-toimingute-statistika#tab-4 Accessed 2020-02-14
35. Statistics Finland: Vehicle stock by year of first registration as of 2018-12-31 [StatFin > Transport and Tourism > Motor vehicle stock > 11ti – Vehicle stock by year of first registration, 2018] (2018). http://pxnet2.stat.fi/PXWeb/pxweb/en/StatFin/StatFin__lii__mkan/ Accessed 2020-02-15
36. Department for Transport Statistics: Vehicle Licensing Statistics: Licensed cars at the end of the year by year of first registration, Great Britain from 1994; also United Kingdom from 2014 [Vehicle ownership, approval and standards > Driver and vehicle record > Table VEH0211a] (2016). https://www.gov.uk/government/collections/vehicles-statistics Accessed 2020-02-14
37. Hellenic Statistical Authority (ELSTAT, P.c.w.M.H.K.: Age distribution of passenger cars in use in Greece as of 2018-12-31 (2019). https://www.statistics.gr/en/home
38. Croatian Chamber of Economy (Private correspondence with Mr. Hrvoje Paver): Age distribution of passenger cars in use in Croatia as of 2017-12-31 (2020)
39. Datahouse Kft. (carinfo.hu): Private correspondence with Mr. Robert Gábor: Age distribution of passenger cars in use in Hungary as of 2017-12-31 (2019)
40. Central Statistics Office Ireland: Vehicle Population by Type of Vehicle, County of Ownership, Fuel Type, Year of Registration and Year, as of 2016-12-31 [StatBank > Road Traffic Volumes > THA17] (2019). https://statbank.cso.ie/px/pxeirestat/Statire/SelectVarVal/Define.asp?maintable=THA17&PLanguage=0 Accessed 2020-02-13
41. Icelandic Transport Authority (Samgöngustofa): Private correspondence with Mr. Friðrik Hjörleifsson: Age distribution of passenger cars in use in Iceland as of 2018-12-31 (2019)
42. Automobile Club d'Italia: Annuario statistico 2017 [Capitolo 3 - Consistenza parco veicoli > TABIII34] (2017). http://www.aci.it/laci/studi-e-ricerche/dati-e-statistiche/annuario-statistico/annuario-statistico-2017.html Accessed 2020-02-21
43. Regitra: Atviri iregistruotu keliu transporto priemoniu parko duomenys (2020-04-03) [State Enterprise "Regitra" > Register of Vehicles of the Republic of Lithuania > Open Data > Open data of the fleet of registered road vehicles (03/04/2020)] (2020). https://www.regitra.lt/lt/paslaugos/duomenu-teikimas/atviri-duomenys-1 Accessed 2020-04-28
44. Luxembourgish data platform (data.public.lu): Parc Automobile du Luxembourg 2017-12-31 [Portail Open Data] (2017). https://data.public.lu/fr/datasets/parc-automobile-du-luxembourg Accessed 2020-02-13
45. CSDD (Ceļu Satiksmes Drošības Direkcija): Private correspondence with Mr. Aldis Lāma (Data source: Latvian Vehicle Register): Number of registered passenger cars by age in Latvia as of 2017-01-01 (2020). https://www.csdd.lv/transportlidzekli/registreto-transportlidzeklu-skait
46. National Statistics Office Malta: Transport Statistics 2017 (reference year 2016) (2017). https://nso.gov.mt/en/publicatons/Pages/Publications-by-Date.aspx Accessed 2020-02-24
47. CBS Statline (Statistics Netherlands): Age distribution of passenger cars in use in the Netherlands as of 2016-12-31 [Data Portal > Personenauto's; voertuigkenmerken, regio's, 1 januari, as of 2017-01-01] (2020). https://opendata.cbs.nl/statline/#/CBS/nl/dataset/71405NED/table?fromstatweb Accessed 2020-02-13
48. Opplysningsrådet for Veitrafikken (Private correspondence with Mr. Pål Bruhn: Age distribution of passenger cars in use in Norway as of 2016-12-31 (2020)
49. Polish Automotive Industry Association (Private correspondence with Mr. Marek Wolfigiel): Age distribution of passenger cars in use in Poland as of 2016-12-31 (2019)
50. Instiuto Nacional de Estatística (Private correspondence with Statistics Portugal (INE)): Age distribution of passenger cars in use in Portugal as of 2016-12-31 (2020)
51. National Institute for Statistics Romania: Registered road vehicles (end of the year), by groups of age, as of 2016 [Economic Statistics > Transports > Road transport > Table TRN103C] (2020). http://statistici.insse.ro:8077/tempo-online/#/pages/tables/insse-table Accessed 2020-02-15
52. Ministry of Interior of the Slovak Republic (Private correspondence with Mr. Boris Križánek: Age distribution of passenger cars in use in the Slovak Republic as of 2016-12-31 (2020). http://www.minv.sk/?ministry-of-interior
53. Transport Analysis: Swedish vehicle statistics 2018 [Trafa > Road Traffic > Vehicle statistics] (2016). https://www.trafa.se/en/road-traffic/vehicle-statistics/ Accessed 2020-02-14
54. Oguchi, M., Fuse, M.: Regional and Longitudinal Estimation of Product Lifespan Distribution: A Case Study for Automobiles and a Simplified Estimation Method. Environmental Science & Technology **49**(3), 1738–1743 (2015). doi:10.1021/es505245q
55. ACEA (European Automobile Manufacturers Association): Historical series 1990-2018: new passenger car registrations by country [Consolidated Registrations - By Country] (2020). https://www.acea.be/statistics/tag/category/by-country-registrations Accessed 2020-02-21
56. UNECE: New road vehicle registrations by vehicle category and fuel type (1993-2018) [Statistical Database of the United Nations Economic Commission for Europe > Transport > Road Vehicle Fleet] (2019).

https://w3.unece.org/PXWeb2015/pxweb/en/STAT/STAT__40-TRTRANS__03-TRRoadFleet/08_en_TRRoadNewVehF_r.px/ Accessed 2020-02-20
57. Kraftfahrt-Bundesamt: Kraftfahrt-Bundesamt: Verkehr in Kilometern (VK) - Zeitreihe: Jahre 2014-2019 (2020). https://www.kba.de/DE/Statistik/Produktkatalog/produkte/Kraftverkehr/vk_uebersicht.html?nn=1146130 Accessed 2020-07-29
58. Eurostat: New registrations of passenger cars 1970-2017 [Road transport equipment - new registration of vehicles (road_eqr) > New registrations of passenger cars by type of motor energy and engine size (road_eqr_carmot)] (2019). https://ec.europa.eu/eurostat/web/transport/data/database Accessed 2020-03-11
59. Statistics Austria: Passenger cars, lorries and two wheelers - registrations of new and used motor vehicles 1992 to 2018 [Statistics > Transport > Road] (2018). https://www.statistik.at/web_en/statistics/EnergyEnvironmentInnovationMobility/transport/road/registration_of_new_vehicles/index.html Accessed 2020-02-20
60. Fédération Belge et Luxembourgeoise de l'automobile et du cycle: Evolution des immatriculations de voitures neuves et d'occasion (Evolution of new car and used car registrations) (2019). http://www.febiac.be/public/statistics.aspx?FID=23 Accessed 2020-02-20
61. Swiss Federal Statistics Office: Road vehicles - New registrations [Neue Inverkehrsetzungen von Strassenfahrzeugen nach Kantonen und technischen Merkmalen, Data from Swiss Federal Roads Office] (2018). https://www.bfs.admin.ch/bfs/en/home/statistics/mobility-transport/transport-infrastructure-vehicles/vehicles/road-new-registrations.html Accessed 2020-02-21
62. Statistical Service of the Republic of Cyprus: Registration of passenger saloon cars (2003-2020) by month and registration type (new and used cars) [Services > Transport and Communications > Key figures] (2020). https://www.mof.gov.cy/mof/cystat/statistics.nsf/services_72main_en/services_72main_en?OpenForm&sub=2&sel=2 Accessed 2020-02-21
63. Car Importers Association of the Czech Republic (SDA): Registration of new passenger cars in the Czech Republic 1993-2016 [Statistics > Registrations of new passenger cars] (2020). http://portal.sda-cia.cz/stat.php Accessed 2020-02-20
64. Kraftfahrt-Bundesamt: Neuzulassungen in den Jahren 1960 bis 2018 nach Fahrzeugklassen [Statistik > Fahrzeuge > Neuzulassungen > Fahrzeugklassen und Aufbauarten] (2020). https://www.kba.de/DE/Statistik/Fahrzeuge/Neuzulassungen/FahrzeugklassenAufbauarten/fz_n_fzkl_aufb_archiv/2018/2018_n_fzkl_zeitreihe.html Accessed 2020-02-21
65. Statistics Denmark: New registration of motor vehicles by type of vehicle (1992-2016) [StatBank Denmark > Prices and consumption > Selection from table BIL5] (2020). https://www.statbank.dk/ Accessed 2020-02-21
66. Spanish Dirección General de Tráfico: Series históricas: Matriculaciones por tipos de vehículos [Seguridad vial > Estadísticas e indicadores > Matriculaciones definitivas > Series históricas] (2020). http://www.dgt.es/es/seguridad-vial/estadisticas-e-indicadores/matriculaciones-definitivas/series-historicas/ Accessed 2020-02-21
67. Statistics Estonia: First registrations of vehicles 1996-2016: Registered new passenger cars [Transport > Registered motor vehicles > Table TS322] (2020). http://andmebaas.stat.ee/?lang=en Accessed 2020-02-21
68. Statistics Finland: First registrations of motor vehicles by Vehicle class, Year and Information 1956-2016 [StatFin >> Transport and Tourism >> First registrations of motor vehicles >> 11cb] (2019). http://pxnet2.stat.fi/PXWeb/pxweb/en/StatFin/StatFin__lii__merek/statfin_merek_pxt_11cb.px/table/tableViewLayout1/ Accessed 2020-02-20
69. République Française - Données et études statistiques: New vehicle registration data in France 1990-2018 (New and used cars) [Tables "0.I.F.1 Immatriculations neuves par genre de véhicule" and "0.I.F.2 Immatriculations d'occasion par genre de véhicule"] (2020). https://www.statistiques.developpement-durable.gouv.fr/donnees-sur-les-immatriculations-des-vehicules Accessed 2020-02-20
70. Department for Transport Statistics: Cars registered for the first time by propulsion and fuel type: Great Britain (2001-2018) and United Kingdom (2015-2018) [Transport > Driving and road transport > Vehicle ownership, approval and standards > Driver and vehicle record > Table VEH0253] (2020). https://www.gov.uk/government/statistical-data-sets/veh02-licensed-cars Accessed 2020-04-25
71. Hellenic Statistical Authority (ELSTAT): Motor Vehicles, both new and used, put into circulation for the first time in Greece (1985-2018) [Vehicle Licensing] (2019). https://www.statistics.gr/en/statistics/-/publication/SME24/2018-M03 Accessed 2020-02-20
72. Croatian Chamber of Economy (Private correspondence with Mr. Hrvoje Paver): Sales of new and used vehicles, 2008-2016 (2020)
73. Datahouse Kft. (carinfo.hu): Private correspondence with Mr. Robert Gábor: Number of new registrations of new and used passenger cars in Hungary (1999-2018) (2019). https://www.ksh.hu/stadat_annual_4_6
74. Irish Central Statistics Office: Vehicles Licensed for the First Time (1954-2018) by Type of Vehicle Registration and Year [TEA10: Vehicles Licensed for the First Time (1954 - 2018) by Type of Vehicle Registration and Year] (2019). https://statbank.cso.ie/px/pxeirestat/Statire/SelectVarVal/Define.asp?maintable=TEA10 Accessed 2020-02-20
75. Icelandic Transport Authority (Samgöngustofa): Private correspondence with Mr. Friðrik Hjörleifsson: New registrations of passenger cars (1982-2016) (2020)
76. ANFIA: Statistical yearbook 2019 [Associazone Nazionale Filiera Industria Automobilistica > Studi e Statistiche > Automobile in cifre - Annuario statistico > Immatricolazioni] (2020). https://www.anfia.it/it/automobile-in-cifre/statistiche-italia/immatricolazioni Accessed 2020-02-20
77. Statistics Office of the Principality of Liechtenstein: New registrations of passenger cars 2003-2016 [Fahrzeugstatistik > Erstzulassungen] (2020). https://www.llv.li/inhalt/12632/amtsstellen/fahrzeugstatistik-erstzulassungen Accessed 2020-02-21
78. STATEC (Statistics Portal Grand Duchy of Luxembourg): New registrations of new motor vehicles by vehicle type 1965-2019 [Statistics > Enterprises > Transport > Road Transport > Vehicles] (2020). https://statistiques.public.lu/fr/index.html Accessed 2020-02-21
79. CSDD (Ceļu Satiksmes Drošības Direkcija): Private correspondence with Mr. Aldis Lāma (Data source: Latvian Vehicle Register): New registrations of passenger cars by age (1997-2019) (2020)
80. National Statistics Office Malta: Transport Statistics 2001-2019 (data from 19 annual statistics reports), Lascaris (2019). https://nso.gov.mt/en/publicatons/Pages/Publications-by-Date.aspx Accessed 2020-02-24
81. Stichting BOVAG-RAI Mobiliteit: Mobiliteit in Cijfers: Auto's 2018-2019. Technical report (2020). www.bovagrai.info
82. Opplysningsrådet for Veitrafikken (Private correspondence with Mr. Pål Bruhn): Age distribution of new registrations of (new and used) passenger cars in Norway (2000-2019) (2020)
83. Polish Automotive Industry Association (Private correspondence with Mr. Marek Wolfigiel): New registrations of passenger cars (2005-2016) (2020)
84. National Institute for Statistics Romania: New registrations of road vehicles for passenger transport, by macroregions, development regions and counties (new passenger cars: 2000-2016, second-hand imported passenger cars: 2007-2016) [Transports > Road transport > Table TRN102A] (2020). http://statistici.insse.ro:8077/tempo-online/#/pages/tables/insse-table Accessed 2020-02-21
85. Ministry of Interior of the Slovak Republic: Number of new registrations of passenger cars in Slovakia (2003-2016) [Polícia > Prezídium Policajného

zboru > Doklady a evidencie > Štatistiky > Evidencia vozidiel > Počet novoevidovaných vozidiel] (2020). http://www.minv.sk/?pocet-novoevidovanych-vozidiel

86. Statistical Office of the Republic of Slevenia (SiStat): Road vehicles and first registrations of road vehicles by year, type of vehicle and measure (1998-2016) [Economy > Transport > Registered road vehicles > Road vehicles and first registrations of road vehicles by type of vehicle, Slovenia, annually] (2020). https://pxweb.stat.si/SiStatDb/pxweb/en/20_Ekonomsko/20_Ekonomsko__22_transport__08_22221_reg_cestna_vozila/2222102S.px/table/tableViewLayout2/ Accessed 2020-02-21
87. Statistics Sweden (Private correspondence with Mr. Magnus Nyström): New registrations of passenger cars in Sweden and direct imports thereof (1995-2016) (2020)
88. Statistics Lithuania (Official Statistics Portal of the Republic of Lithuania): Road vehicle registered in the country for the first time: new and used passenger cars [Transport and communications > Road transport indicators > Indicators of road vehicles] (2020). https://osp.stat.gov.lt/statistiniu-rodikliu-analize?indicator=S5R058#/ Accessed 2020-02-20
89. Vanherle, K., Vergeer, R.: Data gathering and analysis to improve the understanding of 2nd hand car and LDV markets and implications for the cost effectiveness and social equity of LDV CO2 regulations - Final Report for: DG Climate Action, Brussels. Technical report (2016). http://www.tmleuven.be
90. PORDATA: Vehicles registered: total and by type of vehicle [Pordata > Transport > Road > Cars registered by type] (2019). https://www.pordata.pt/
91. Statistics Portugal: Transport and communication statistics (2012-2016), Transport statistics (2001-2011) (2020). https://ine.pt/ Accessed 2020-04-25
92. Eurostat: Population on 1 January (online data code: TPS00001): Demography and migration > Population (2020). https://ec.europa.eu/eurostat/databrowser/view/tps00001/default/table?lang=en Accessed 2020-02-15
93. Mehlhart, G., Merz, C., Akkermans, L., Jordal-Jorgensen, J.: European second-hand car market analysis: Final Report for DG Climate Action at the European Commission (Contract No. 07.0307/2009/549021/SER/C5, Öko-Institut e.V., Transport & Mobility Leuven, COWI). Technical report (2011). https://www.oeko.de/oekodoc/1114/2011-005-en.pdf
94. Car Importers Association of the Czech Republic (SDA): Registration of used passenger cars 2007-2016 (resolved by age) and exports of passenger cars 2006-2016 (totals) in the Czech Republic [Statistics > Registrations of used cars] (2020). http://portal.sda-cia.cz/stat.php?o#rok=2019&mesic=12&kat=M1&vyb=oje&upr=&obd=r&jine=false&lang=EN&str=ojehttps://www.sda-cia.cz/repository-volnedostupna?lang=EN
95. Estonian Road Administration: Registrations of used vehicles in Estonia, 2006-2016 [Statistics > Vehicles > Statistics on vehicle operations] (2020). https://www.mnt.ee/et Accessed 2020-03-02
96. Autoalan Tiedotuskeskus: Imported used cars (2020). http://www.aut.fi/en/statistics/long-term_statistics/imported_used_cars Accessed 2020-04-25
97. Irish Central Statistics Office: Profile of Used (Imported) Cars Registered by Age, Type of Registration and Year (2005-2016) (2020). https://statbank.cso.ie/ Accessed 2020-04-18
98. ANFIA: Export-Import: Customs Data (Table "Automotive industry": Number of used imports and exports 2012-2016) [Associazone Nazionale Filiera Industria Automobilistica > Studi e Statistiche > Automobile in cifre - Annuario statistico > Trade automotive] (2019). https://www.anfia.it/it/automobile-in-cifre/statistiche-italia/trade-automotive Accessed 2020-04-26
99. STATEC (Statistics Portal Grand Duchy of Luxembourg): Private correspondence with Ms. Nadine Urhausen: Imports to Luxembourg and exports from Luxembourg of used cars during the period 2005 to 2018 (2019)
100. Stichting BOVAG-RAI Mobiliteit: Mobiliteit in Cijfers: Auto's 2010/2011, 2013/2014, 2018/2019. Technical report (2020). www.bovagrai.info
101. CBS Statline (Statistics Netherlands): Motor vehicles; demolition, export and other failure, age of the car (2000-2018) [Data Portal > Motorvoertuigen; sloop, export en overige uitval, leeftijd auto] (2020). https://opendata.cbs.nl/statline/#/CBS/nl/dataset/80357ned/table?fromstatweb Accessed 2020-04-18
102. Trafikanalys (Private correspondence with Ms. Anette Myhr): Imports and exports of passenger cars in Sweden (2009-2016) (2019)
